# Supplementary material for: Historical Selection, Adaptation Signatures, and Ambiguity of Introgressions in Wheat
Source: Int J Mol Sci. 2023 May 7;24(9):8390. doi: 10.3390/ijms24098390 (PMC10179502; doi:10.3390/ijms24098390)
Supplement: Supplementary file 1 [file ijms-24-08390-s001.zip › ijms-2362800-supplementary.pdf]

International Journal of
Molecular Sciences

MDPI

Article

Historical Selection, Adaptation Signatures, and Ambiguity of Introgressions in Wheat

Demissew Sertse[1*]; Frank You[2]; Valentyna Klymiuk[3]; Jemanesh K. Haile[3]; Amidou N'Diaye[3]; Curtis J. Pozniak [3]; Sylvie Cloutier[2], Sateesh Kagale[1*]

1 Aquatic and Crop Resource Development, National Research Council Canada, Saskatoon, Saskatchewan, Canada
2 Ottawa Research and Development Centre, Agriculture and Agri-Food Canada, Ottawa, Ontario, Canada; frank.you@agr.gc.ca (F.M.Y.); sylviej.cloutier@agr.gc.ca (S.C.)
3 Crop Development Centre, University of Saskatchewan, Saskatoon, Saskatchewan, Canada; valentyna.klymiuk@usask.ca (V.K.); jemanesh.haile@usask.ca (J.K.H.); amidou.ndiaye@usask.ca (A.N.); curtis.pozniak@usask.ca (C.J.P.)
* Correspondence: dmsertse@gmail.com (D.S.); Sateesh.Kagale@nrc-cnrc.gc.ca (S.K.)

Table S1. The list of accessions in main (n=921 of the exome data set and ancestral coefficient of each accession based on ancestral population defined at K=8.

Accessions	Q1	Q2	Q3	Q4	Q5	Q6	Q7	Q8	Subpop
AUS_DAS5-000894	0.9999	0.0000	0.0000	0.0000	0.0000	0.0000	0.0000	0.0000	AUS
AUS_DAS5-000826	0.9500	0.0000	0.0000	0.0000	0.0000	0.0500	0.0000	0.0000	AUS
AUS_DAS5-000719	0.9492	0.0000	0.0000	0.0000	0.0000	0.0508	0.0000	0.0000	AUS
AUS_DAS5-000588	0.9310	0.0090	0.0000	0.0000	0.0000	0.0600	0.0000	0.0000	AUS
AUS_DAS5-000820	0.9040	0.0000	0.0000	0.0000	0.0000	0.0960	0.0000	0.0000	AUS
AUS_DAS5-000668	0.8886	0.0125	0.0000	0.0000	0.0000	0.0989	0.0000	0.0000	AUS
AUS_DAS5-000929	0.8855	0.0000	0.0000	0.0000	0.0042	0.1103	0.0000	0.0000	AUS
AUS_DAS5-000574	0.8630	0.0000	0.0000	0.0709	0.0000	0.0661	0.0000	0.0000	AUS
AUS_DAS5-000713	0.8398	0.0000	0.0000	0.0000	0.0000	0.1601	0.0000	0.0000	AUS
AUS_DAS5-000816	0.8226	0.0000	0.0000	0.0892	0.0000	0.0882	0.0000	0.0000	AUS
AUS_DAS5-000916	0.8095	0.0276	0.0000	0.0000	0.0000	0.0553	0.1076	0.0000	AUS
AUS_DAS5-000824	0.8067	0.0000	0.0000	0.0000	0.0000	0.1932	0.0000	0.0000	AUS
AUS_DAS5-000821	0.8053	0.0000	0.0000	0.0000	0.0000	0.1946	0.0000	0.0000	AUS
AUS_DAS5-000734	0.7892	0.0000	0.0000	0.0000	0.0000	0.2107	0.0000	0.0000	AUS
AUS_DAS5-000642	0.7860	0.0000	0.0000	0.0000	0.0000	0.2140	0.0000	0.0000	AUS
AUS_DAS5-000793	0.7413	0.0000	0.0000	0.0000	0.0000	0.1518	0.0872	0.0196	AUS
AUS_DAS5-000759	0.7304	0.0793	0.0000	0.0000	0.0000	0.1850	0.0000	0.0053	AUS
AUS_DAS5-000875	0.7127	0.0267	0.0000	0.0000	0.0000	0.2606	0.0000	0.0000	AUS
AUS_DAS5-000667	0.7049	0.0000	0.0000	0.0000	0.0000	0.2950	0.0000	0.0000	AUS
AUS_DAS5-000996	0.6931	0.0170	0.0000	0.0745	0.0000	0.1452	0.0000	0.0702	AUS
AUS_DAS5-000887	0.6832	0.0000	0.0000	0.0000	0.0000	0.3167	0.0000	0.0000	AUS
AUS_DAS5-SUNCO	0.6794	0.0000	0.0000	0.1782	0.1424	0.0000	0.0000	0.0000	AUS
AUS_DAS5-000643	0.6787	0.0139	0.0000	0.0000	0.0000	0.3074	0.0000	0.0000	AUS
AUS_DAS5-000146	0.6695	0.0000	0.0000	0.1014	0.0082	0.1928	0.0211	0.0070	AUS
AUS_DAS5-000545	0.6574	0.0000	0.0000	0.0255	0.0622	0.2548	0.0000	0.0000	AUS

Accessions	Q1	Q2	Q3	Q4	Q5	Q6	Q7	Q8	Subpop
AUS_DAS5-000232	0.6534	0.0000	0.0329	0.0968	0.0000	0.2168	0.0000	0.0000	AUS
AUS_DAS5-000504	0.6422	0.0216	0.0000	0.0000	0.0000	0.3361	0.0000	0.0000	AUS
AUS_DAS5-000192	0.6415	0.0206	0.0000	0.0000	0.0000	0.3379	0.0000	0.0000	AUS
AUS_DAS5-000710	0.6383	0.0453	0.0000	0.1141	0.0088	0.1584	0.0000	0.0351	AUS
AUS_DAS5-000140	0.6257	0.0352	0.0000	0.0000	0.0000	0.3391	0.0000	0.0000	AUS
AUS_DAS5-000518	0.6195	0.0000	0.0000	0.0000	0.0000	0.3804	0.0000	0.0000	AUS
AUS_DAS5-000553	0.6124	0.0000	0.0000	0.0000	0.0000	0.3875	0.0000	0.0000	AUS
AUS_DAS5-LANG	0.6053	0.0000	0.0000	0.2538	0.1409	0.0000	0.0000	0.0000	AUS
AUS_DAS5-000058	0.6033	0.0000	0.0000	0.0000	0.0000	0.3966	0.0000	0.0000	AUS
AUS_DAS5-000205	0.5967	0.0000	0.0000	0.1465	0.0000	0.2568	0.0000	0.0000	AUS
AUS_DAS5-000156	0.5947	0.0469	0.0000	0.0000	0.0000	0.3584	0.0000	0.0000	AUS
AUS_DAS5-SUNGUARD	0.5889	0.0000	0.0000	0.2830	0.1281	0.0000	0.0000	0.0000	AUS
AUS_DAS5-000191	0.5842	0.0000	0.0000	0.0000	0.0000	0.4157	0.0000	0.0000	AUS
AUS_DAS5-000117	0.5738	0.0516	0.0000	0.0000	0.0000	0.3746	0.0000	0.0000	AUS
AUS_DAS5-000154	0.5700	0.0000	0.0000	0.0000	0.0000	0.4300	0.0000	0.0000	AUS
AUS_DAS5-000148	0.5649	0.0253	0.0000	0.1609	0.0000	0.2056	0.0433	0.0000	AUS
AUS_DAS5-000489	0.5629	0.0000	0.0099	0.0000	0.0000	0.4149	0.0000	0.0123	AUS
AUS_DAS5-002230	0.5620	0.0000	0.0000	0.4380	0.0000	0.0000	0.0000	0.0000	AUS
AUS_DAS5-000183	0.5568	0.0006	0.0000	0.0000	0.0000	0.4425	0.0000	0.0000	AUS
BRA_DAS5-001730	0.5482	0.0000	0.0000	0.4518	0.0000	0.0000	0.0000	0.0000	AUS
AUS_DAS5-000222	0.5437	0.0000	0.0000	0.1369	0.0694	0.2290	0.0015	0.0195	AUS
AUS_DAS5-DERRIMUT	0.5412	0.0000	0.0000	0.3133	0.1454	0.0000	0.0000	0.0000	AUS
AUS_DAS5-000007	0.5378	0.0346	0.0000	0.0000	0.0046	0.3982	0.0248	0.0000	AUS
AUS_DAS5-000533	0.5360	0.0306	0.0212	0.0567	0.0186	0.3369	0.0000	0.0000	AUS
AUS_DAS5-000102	0.5314	0.0000	0.0000	0.1496	0.0591	0.0425	0.1271	0.0903	AUS
AUS_DAS5-000638	0.5253	0.0000	0.0427	0.1414	0.0000	0.2906	0.0000	0.0000	AUS
AUS_DAS5-EGA-HUME	0.5242	0.0000	0.0000	0.4168	0.0589	0.0000	0.0000	0.0000	AUS
AUS_DAS5-000215	0.5173	0.0000	0.0000	0.0000	0.0000	0.4827	0.0000	0.0000	AUS
AUS_DAS5-000221	0.5150	0.0000	0.0000	0.1636	0.1024	0.1976	0.0213	0.0000	AUS
AUS_DAS5-000496	0.5072	0.0000	0.0000	0.0000	0.0000	0.4927	0.0000	0.0000	AUS
AUS_DAS5-000847	0.5021	0.0106	0.0000	0.3076	0.0000	0.1797	0.0000	0.0000	AUS
AUS_DAS5-000563	0.4981	0.0168	0.0000	0.0000	0.0000	0.4851	0.0000	0.0000	AUS
AUS_DAS5-000135	0.4934	0.0000	0.0000	0.0000	0.0000	0.4828	0.0000	0.0238	AUS
AUS_DAS5-STRZELECKI	0.4865	0.0000	0.0000	0.4498	0.0448	0.0000	0.0189	0.0000	AUS
AUS_DAS5-000472	0.4798	0.0054	0.0223	0.0720	0.0000	0.4005	0.0077	0.0122	AUS
AUS_DAS5-003328	0.4692	0.0000	0.0000	0.3067	0.0000	0.0000	0.0484	0.1756	AUS
AUS_DAS5-003329	0.4608	0.0000	0.0000	0.3535	0.0000	0.0000	0.0000	0.1856	AUS
AUS_DAS5-GLADIUS	0.4571	0.0005	0.0000	0.4228	0.0869	0.0000	0.0002	0.0324	AUS
AUS_DAS5-000145	0.4541	0.0326	0.0000	0.1614	0.0690	0.2548	0.0281	0.0000	AUS
AUS_DAS5-000127	0.4388	0.0000	0.0000	0.0788	0.2588	0.2236	0.0000	0.0000	AUS
AUS_DAS5-000123	0.4212	0.0077	0.0000	0.1164	0.1261	0.3286	0.0000	0.0000	AUS

Accessions	Q1	Q2	Q3	Q4	Q5	Q6	Q7	Q8	Subpop
AUS_DAS5-000478	0.3932	0.0588	0.0000	0.0321	0.0238	0.3212	0.0000	0.1708	AUS
AUS_DAS5-000225	0.3555	0.0216	0.0000	0.2776	0.0731	0.2722	0.0000	0.0000	AUS
AUS_DAS5-000402	0.3542	0.0000	0.0000	0.3436	0.0000	0.3021	0.0000	0.0000	AUS
MEX_WSC-1-8	0.0000	0.0896	0.0000	0.0000	0.0000	0.9103	0.0000	0.0000	AUSb
AUS_DAS5-000443	0.0000	0.0000	0.0000	0.1658	0.0000	0.8342	0.0000	0.0000	AUSb
MEX_DAS5-002249	0.0000	0.1725	0.0000	0.0000	0.0000	0.8274	0.0000	0.0000	AUSb
AUS_DAS5-000053	0.2351	0.0000	0.0000	0.0000	0.0000	0.7649	0.0000	0.0000	AUSb
AUS_DAS5-000551	0.0000	0.0000	0.0000	0.2489	0.0000	0.7510	0.0000	0.0000	AUSb
AUS_DAS5-000411	0.0000	0.0000	0.0000	0.2490	0.0000	0.7509	0.0000	0.0000	AUSb
AUS_DAS5-000041	0.0000	0.0384	0.0000	0.2232	0.0000	0.7384	0.0000	0.0000	AUSb
AUS_DAS5-000074	0.2581	0.0037	0.0000	0.0000	0.0000	0.7381	0.0000	0.0000	AUSb
AUS_DAS5-000310	0.0000	0.0160	0.0000	0.2538	0.0000	0.7301	0.0000	0.0000	AUSb
AUS_DAS5-000368	0.0000	0.0000	0.0000	0.2392	0.0234	0.7179	0.0001	0.0193	AUSb
AUS_DAS5-000071	0.2920	0.0000	0.0000	0.0000	0.0000	0.7079	0.0000	0.0000	AUSb
AUS_DAS5-000353	0.0000	0.0000	0.0133	0.3085	0.0000	0.6781	0.0000	0.0000	AUSb
AUS_DAS5-000260	0.0000	0.0015	0.0000	0.2599	0.0033	0.6704	0.0649	0.0000	AUSb
AUS_DAS5-000358	0.0000	0.0000	0.0000	0.3403	0.0000	0.6597	0.0000	0.0000	AUSb
AUS_DAS5-000373	0.0000	0.0000	0.0000	0.3437	0.0000	0.6563	0.0000	0.0000	AUSb
AUS_DAS5-000178	0.3510	0.0000	0.0000	0.0000	0.0000	0.6490	0.0000	0.0000	AUSb
AUS_DAS5-000442	0.0000	0.0000	0.0000	0.3811	0.0000	0.6188	0.0000	0.0000	AUSb
AUS_DAS5-000160	0.3371	0.0606	0.0000	0.0000	0.0000	0.6022	0.0000	0.0000	AUSb
AUS_DAS5-000424	0.0000	0.0000	0.0000	0.3290	0.0709	0.5931	0.0000	0.0070	AUSb
AUS_DAS5-000244	0.0905	0.0939	0.0000	0.2235	0.0000	0.5920	0.0000	0.0000	AUSb
AUS_DAS5-000462	0.3034	0.1056	0.0000	0.0000	0.0000	0.5910	0.0000	0.0000	AUSb
AUS_DAS5-000029	0.0000	0.0000	0.0427	0.3244	0.0000	0.5753	0.0499	0.0077	AUSb
AUS_DAS5-000055	0.3000	0.0000	0.0000	0.1275	0.0000	0.5724	0.0000	0.0000	AUSb
AUS_DAS5-000413	0.0000	0.0000	0.0000	0.4293	0.0000	0.5707	0.0000	0.0000	AUSb
AUS_DAS5-000524	0.4305	0.0000	0.0000	0.0000	0.0000	0.5695	0.0000	0.0000	AUSb
AUS_DAS5-000396	0.0000	0.0000	0.0000	0.4342	0.0000	0.5555	0.0103	0.0000	AUSb
AUS_DAS5-000562	0.4304	0.0186	0.0000	0.0000	0.0000	0.5510	0.0000	0.0000	AUSb
AUS_DAS5-000166	0.4593	0.0000	0.0000	0.0000	0.0000	0.5407	0.0000	0.0000	AUSb
AUS_DAS5-000430	0.0000	0.0000	0.0000	0.4692	0.0000	0.5308	0.0000	0.0000	AUSb
AUS_DAS5-000194	0.4719	0.0000	0.0000	0.0000	0.0000	0.5280	0.0000	0.0000	AUSb
AUS_DAS5-000503	0.4557	0.0163	0.0000	0.0000	0.0000	0.5280	0.0000	0.0000	AUSb
AUS_DAS5-000035	0.0000	0.0066	0.0000	0.4336	0.0319	0.5253	0.0025	0.0000	AUSb
AUS_DAS5-000186	0.4283	0.0569	0.0000	0.0000	0.0000	0.5148	0.0000	0.0000	AUSb
AUS_DAS5-000491	0.4864	0.0000	0.0000	0.0000	0.0000	0.5136	0.0000	0.0000	AUSb
AUS_DAS5-000301	0.0328	0.0000	0.0000	0.4579	0.0000	0.5093	0.0000	0.0000	AUSb
AUS_DAS5-000004	0.4909	0.0000	0.0000	0.0000	0.0000	0.5090	0.0000	0.0000	AUSb
AUS_DAS5-000432	0.0000	0.0000	0.0252	0.4665	0.0000	0.5083	0.0000	0.0000	AUSb
AUS_DAS5-000118	0.4192	0.0761	0.0000	0.0000	0.0000	0.5047	0.0000	0.0000	AUSb

Accessions	Q1	Q2	Q3	Q4	Q5	Q6	Q7	Q8	Subpop
AUS_DAS5-000079	0.4961	0.0000	0.0000	0.0000	0.0000	0.5038	0.0000	0.0000	AUSb
AUS_DAS5-000094	0.4765	0.0307	0.0000	0.0000	0.0000	0.4927	0.0000	0.0000	AUSb
AUS_DAS5-000452	0.2367	0.0018	0.0000	0.2256	0.0295	0.4899	0.0000	0.0165	AUSb
AUS_DAS5-000406	0.0000	0.0495	0.0000	0.4426	0.0027	0.4568	0.0001	0.0483	AUSb
AUS_DAS5-000474	0.3473	0.0115	0.0000	0.1021	0.0319	0.4302	0.0000	0.0771	AUSb
AUS_DAS5-000121	0.3324	0.0000	0.0000	0.0469	0.1240	0.4237	0.0730	0.0000	AUSb
AUS_DAS5-000330	0.2616	0.0000	0.0000	0.3120	0.0000	0.4179	0.0085	0.0000	AUSb
ROM_DAS5-002281	0.0000	0.3349	0.0000	0.0350	0.0183	0.3521	0.2597	0.0000	AUSb
FIN_DAS5-002283	0.0000	0.0000	0.0000	0.0000	0.9999	0.0000	0.0000	0.0000	EEU
FSV_DAS5-002663	0.0000	0.0000	0.0000	0.0000	0.9999	0.0000	0.0000	0.0000	EEU
FSV_DAS5-003116	0.0000	0.0000	0.0000	0.0000	0.9999	0.0000	0.0000	0.0000	EEU
FSV_DAS5-003140	0.0000	0.0000	0.0000	0.0000	0.9999	0.0000	0.0000	0.0000	EEU
FSV_DAS5-003174	0.0000	0.0000	0.0000	0.0000	0.9999	0.0000	0.0000	0.0000	EEU
FSV_DAS5-003208	0.0000	0.0000	0.0000	0.0000	0.9999	0.0000	0.0000	0.0000	EEU
SPA_WATKINS-007454	0.0000	0.0000	0.0000	0.0000	0.9999	0.0000	0.0000	0.0000	EEU
FSV_DAS5-003191	0.0000	0.0000	0.0000	0.0000	0.9999	0.0000	0.0000	0.0000	EEU
ROM_DAS5-002943	0.0000	0.0000	0.0000	0.0000	0.9999	0.0000	0.0000	0.0000	EEU
FSV_DAS5-003195	0.0000	0.0000	0.0000	0.0000	0.9999	0.0000	0.0000	0.0000	EEU
FSV_DAS5-003183	0.0000	0.0000	0.0000	0.0000	0.9980	0.0000	0.0019	0.0000	EEU
FSV_DAS5-003164	0.0000	0.0000	0.0000	0.0000	0.9921	0.0000	0.0078	0.0000	EEU
UNK_WATKINS-007096	0.0000	0.0000	0.0000	0.0000	0.9910	0.0000	0.0090	0.0000	EEU
FSV_DAS5-003166	0.0000	0.0000	0.0000	0.0000	0.9879	0.0000	0.0121	0.0000	EEU
IND_WATKINS-007604	0.0000	0.0000	0.0000	0.0138	0.9861	0.0000	0.0000	0.0000	EEU
FSV_DAS5-003175	0.0000	0.0000	0.0000	0.0000	0.9822	0.0000	0.0178	0.0000	EEU
BRA_WATKINS-007179	0.0000	0.0000	0.0000	0.0000	0.9787	0.0000	0.0213	0.0000	EEU
TUR_WATKINS-007033	0.0000	0.0000	0.0000	0.0000	0.9786	0.0000	0.0213	0.0000	EEU
FSV_DAS5-002642	0.0000	0.0000	0.0000	0.0000	0.9772	0.0000	0.0228	0.0000	EEU
FSV_DAS5-003163	0.0000	0.0077	0.0150	0.0000	0.9754	0.0018	0.0000	0.0000	EEU
FSV_DAS5-003075	0.0000	0.0000	0.0036	0.0000	0.9740	0.0000	0.0000	0.0224	EEU
FSV_DAS5-002832	0.0000	0.0094	0.0218	0.0000	0.9688	0.0000	0.0000	0.0000	EEU
KAZ_DAS5-004324	0.0000	0.0000	0.0062	0.0000	0.9602	0.0000	0.0335	0.0000	EEU
BLR_DAS5-004326	0.0000	0.0000	0.0000	0.0000	0.9585	0.0327	0.0088	0.0000	EEU
IRQ_WATKINS-007697	0.0000	0.0000	0.0000	0.0000	0.9488	0.0000	0.0511	0.0000	EEU
CHN_WATKINS-007274	0.0000	0.0000	0.0000	0.0549	0.9451	0.0000	0.0000	0.0000	EEU
AZE_WATKINS-007170	0.0000	0.0000	0.0000	0.0000	0.9413	0.0000	0.0586	0.0000	EEU
RUS_DAS5-003961	0.0000	0.0000	0.0000	0.0602	0.9397	0.0000	0.0000	0.0000	EEU
BUL_WATKINS-007565	0.0000	0.0000	0.0057	0.0000	0.9374	0.0000	0.0000	0.0569	EEU
ROM_DAS5-002946	0.0000	0.0000	0.0245	0.0021	0.9368	0.0000	0.0000	0.0366	EEU
FSV_DAS5-002899	0.0000	0.0000	0.0000	0.0000	0.9309	0.0000	0.0690	0.0000	EEU
CHN_PI-70613	0.0000	0.0000	0.0000	0.0726	0.9274	0.0000	0.0000	0.0000	EEU
UNK_WATKINS-007099	0.0000	0.0000	0.0000	0.0000	0.9272	0.0000	0.0728	0.0000	EEU

Accessions	Q1	Q2	Q3	Q4	Q5	Q6	Q7	Q8	Subpop
FSV_DAS5-002431	0.0000	0.0000	0.0000	0.0742	0.9257	0.0000	0.0000	0.0000	EEU
FSV_DAS5-003162	0.0000	0.0000	0.0000	0.0000	0.9253	0.0000	0.0746	0.0000	EEU
IND_WATKINS-007588	0.0005	0.0000	0.0000	0.0263	0.9103	0.0000	0.0629	0.0000	EEU
FSV_DAS5-002263	0.0000	0.0000	0.0327	0.0000	0.9026	0.0000	0.0277	0.0369	EEU
BUL_WATKINS-007763	0.0000	0.0000	0.0447	0.0000	0.8888	0.0000	0.0302	0.0362	EEU
POL_DAS5-002877	0.0000	0.0000	0.0000	0.0000	0.8861	0.0000	0.1139	0.0000	EEU
FSV_DAS5-002888	0.0000	0.0000	0.0000	0.0000	0.8737	0.0000	0.1262	0.0000	EEU
FSV_DAS5-002227	0.0000	0.0002	0.0093	0.0838	0.8645	0.0422	0.0000	0.0000	EEU
POL_DAS5-002868	0.0000	0.0000	0.0000	0.0203	0.8603	0.0000	0.0000	0.1193	EEU
FSV_DAS5-002099	0.0000	0.0231	0.0153	0.0000	0.8559	0.0000	0.1057	0.0000	EEU
YUG_WATKINS-007292	0.0000	0.0000	0.0000	0.0000	0.8508	0.0000	0.1179	0.0313	EEU
NOR_DAS5-003891	0.0028	0.0000	0.0000	0.0000	0.8410	0.0000	0.0000	0.1562	EEU
POL_DAS5-002869	0.0000	0.0000	0.0000	0.1325	0.8402	0.0000	0.0000	0.0272	EEU
POL_DAS5-002862	0.0000	0.0000	0.0000	0.0000	0.8342	0.0000	0.1657	0.0000	EEU
UKR_DAS5-004138	0.0000	0.0000	0.0000	0.0000	0.8270	0.0000	0.1729	0.0000	EEU
HUN_DAS5-002090	0.0027	0.0066	0.1165	0.0505	0.8236	0.0000	0.0000	0.0000	EEU
FSV_DAS5-002416	0.0000	0.0000	0.1158	0.0000	0.8115	0.0000	0.0727	0.0000	EEU
EUR_DAS5-004508	0.0129	0.0000	0.0000	0.0000	0.8063	0.0000	0.1611	0.0197	EEU
FSV_DAS5-002640	0.0000	0.0572	0.0990	0.0300	0.8039	0.0000	0.0000	0.0098	EEU
POL_DAS5-002878	0.0000	0.0000	0.0000	0.0000	0.8011	0.0000	0.1988	0.0000	EEU
EGY_WATKINS-007325	0.0067	0.0000	0.0106	0.0000	0.7930	0.0000	0.1896	0.0000	EEU
FSV_DAS5-003248	0.0000	0.0000	0.0530	0.0000	0.7926	0.0000	0.0000	0.1544	EEU
BUL_WATKINS-007563	0.0000	0.0000	0.0109	0.0000	0.7900	0.0000	0.1620	0.0370	EEU
UKR_DAS5-004471	0.0015	0.0144	0.0564	0.0000	0.7865	0.0000	0.0592	0.0820	EEU
ROM_DAS5-002945	0.0092	0.0069	0.0656	0.0000	0.7855	0.0158	0.0991	0.0179	EEU
FSV_WATKINS-007536	0.0000	0.0295	0.0512	0.0000	0.7743	0.0000	0.1450	0.0000	EEU
FSV_DAS5-002144	0.0000	0.0000	0.0000	0.0000	0.7717	0.0000	0.0400	0.1882	EEU
YUG_WATKINS-007290	0.0000	0.0000	0.0000	0.0000	0.7715	0.0000	0.2285	0.0000	EEU
FSV_DAS5-002968	0.0122	0.0000	0.0284	0.0000	0.7619	0.0000	0.0000	0.1974	EEU
AUS_WATKINS-007166	0.0000	0.0000	0.1083	0.1380	0.7536	0.0000	0.0000	0.0000	EEU
FSV_DAS5-002409	0.0076	0.0263	0.0056	0.0000	0.7522	0.0371	0.0000	0.1712	EEU
FSV_DAS5-002605	0.0000	0.0000	0.0100	0.0000	0.7518	0.0000	0.0232	0.2149	EEU
AUT_DAS5-003343	0.0000	0.0036	0.0319	0.0000	0.7499	0.0031	0.0000	0.2115	EEU
FSV_DAS5-002494	0.0028	0.0112	0.0693	0.0000	0.7431	0.0000	0.0000	0.1737	EEU
UKR_DAS5-002261	0.0182	0.0000	0.0468	0.0000	0.7413	0.0000	0.1937	0.0000	EEU
RUS_DAS5-002610	0.0267	0.0000	0.0000	0.0000	0.7321	0.0000	0.2411	0.0000	EEU
FSV_DAS5-003222	0.0092	0.0063	0.1575	0.0000	0.7258	0.0000	0.0000	0.1011	EEU
FIN_DAS5-002861	0.0000	0.0000	0.0105	0.0000	0.7225	0.0000	0.2669	0.0000	EEU
FSV_DAS5-002752	0.0000	0.0122	0.0000	0.0000	0.7209	0.0000	0.2668	0.0000	EEU
JAP_DAS5-004581	0.0000	0.0000	0.0000	0.0000	0.7126	0.0000	0.2655	0.0218	EEU
USA_DAS5-003109	0.0212	0.0000	0.1225	0.1136	0.7090	0.0000	0.0035	0.0302	EEU

Accessions	Q1	Q2	Q3	Q4	Q5	Q6	Q7	Q8	Subpop
DEN_DAS5-003204	0.0084	0.0071	0.0028	0.0000	0.7069	0.0000	0.2749	0.0000	EEU
MON_DAS5-004267	0.0285	0.0067	0.0692	0.0000	0.6984	0.0000	0.0478	0.1494	EEU
BUL_DAS5-003205	0.0000	0.0000	0.0068	0.0000	0.6981	0.0000	0.0288	0.2662	EEU
ARM_DAS5-001950	0.0054	0.0000	0.2665	0.0000	0.6930	0.0000	0.0076	0.0274	EEU
FSV_DAS5-002142	0.0000	0.0000	0.1540	0.0000	0.6915	0.0000	0.0000	0.1545	EEU
POL_DAS5-002876	0.0000	0.0000	0.0000	0.0000	0.6800	0.0000	0.3200	0.0000	EEU
FSV_DAS5-003019	0.0000	0.0141	0.0815	0.0000	0.6732	0.0584	0.0993	0.0734	EEU
MOZ_DAS5-003855	0.0000	0.0000	0.0000	0.3275	0.6724	0.0000	0.0000	0.0000	EEU
AUT_DAS5-003342	0.0000	0.0024	0.0999	0.0000	0.6706	0.0000	0.0000	0.2271	EEU
FSV_DAS5-003047	0.0000	0.0056	0.2241	0.0000	0.6638	0.0000	0.0000	0.1064	EEU
GRE_WATKINS-007738	0.0000	0.0000	0.0484	0.0000	0.6633	0.0000	0.1083	0.1799	EEU
FSV_DAS5-002121	0.0000	0.0135	0.0867	0.2183	0.6619	0.0000	0.0000	0.0195	EEU
USA_DAS5-004692	0.0000	0.0000	0.1319	0.0657	0.6603	0.0000	0.0259	0.1161	EEU
FSV_DAS5-003050	0.0004	0.0000	0.0000	0.0597	0.6581	0.0000	0.1927	0.0892	EEU
POL_DAS5-002330	0.0000	0.0000	0.0000	0.0000	0.6533	0.0000	0.3467	0.0000	EEU
ARG_DAS5-002553	0.0047	0.0065	0.0021	0.1629	0.6516	0.0000	0.0224	0.1498	EEU
FSV_DAS5-002114	0.0000	0.0000	0.0000	0.0000	0.6504	0.0000	0.0608	0.2888	EEU
YUG_WATKINS-007174	0.0000	0.0000	0.0141	0.0000	0.6491	0.0000	0.0852	0.2515	EEU
FSV_DAS5-002687	0.0000	0.0000	0.0869	0.2692	0.6439	0.0000	0.0000	0.0000	EEU
NET_DAS5-001708	0.0000	0.0000	0.0000	0.0818	0.6415	0.0000	0.2767	0.0000	EEU
BOL_WSC-7-9	0.0000	0.0000	0.0000	0.3532	0.6412	0.0000	0.0000	0.0056	EEU
SWE_DAS5-001544	0.0000	0.0000	0.0000	0.0908	0.6391	0.0000	0.2700	0.0000	EEU
FSV_DAS5-002073	0.0381	0.0000	0.0000	0.0000	0.6327	0.0000	0.3291	0.0000	EEU
POL_DAS5-002863	0.0000	0.0000	0.0000	0.0000	0.6206	0.0000	0.3793	0.0000	EEU
FSV_DAS5-002359	0.0000	0.0000	0.0000	0.0000	0.6205	0.0000	0.3795	0.0000	EEU
FSV_DAS5-002860	0.0000	0.0000	0.0000	0.0000	0.6197	0.0000	0.3802	0.0000	EEU
FSV_DAS5-002965	0.0000	0.0000	0.0000	0.0033	0.6101	0.0000	0.0871	0.2995	EEU
FSV_DAS5-003172	0.0000	0.0000	0.1407	0.0000	0.6057	0.0000	0.0000	0.2535	EEU
AUS_WATKINS-007155	0.0000	0.0000	0.0000	0.2345	0.6022	0.0000	0.1633	0.0000	EEU
ARG_DAS5-004349	0.0000	0.0398	0.0231	0.2708	0.6017	0.0000	0.0000	0.0646	EEU
FSV_DAS5-002833	0.0222	0.0242	0.0326	0.0000	0.6015	0.0000	0.0000	0.3195	EEU
FIN_DAS5-001553	0.0000	0.0019	0.1769	0.2166	0.6008	0.0037	0.0000	0.0000	EEU
FSV_DAS5-003244	0.0000	0.0000	0.2773	0.0000	0.5867	0.0000	0.0000	0.1360	EEU
CRO_Cltr-11223	0.0000	0.0000	0.2541	0.0000	0.5841	0.0000	0.0000	0.1618	EEU
NET_DAS5-001374	0.0000	0.0000	0.0000	0.1652	0.5827	0.0000	0.1501	0.1020	EEU
SWE_DAS5-001790	0.0000	0.0000	0.2527	0.0419	0.5815	0.0000	0.0000	0.1239	EEU
RUS_DAS5-002308	0.0064	0.0000	0.0000	0.0067	0.5809	0.0000	0.4059	0.0000	EEU
ARG_DAS5-002550	0.0003	0.0333	0.0000	0.1659	0.5806	0.0000	0.1332	0.0866	EEU
YUG_WATKINS-007803	0.1276	0.0000	0.0067	0.2259	0.5768	0.0000	0.0629	0.0000	EEU
GRE_WATKINS-007523	0.0056	0.0051	0.0590	0.0000	0.5764	0.0000	0.0293	0.3247	EEU
FSV_DAS5-004476	0.0380	0.0346	0.1371	0.0000	0.5761	0.0320	0.0365	0.1457	EEU

Accessions	Q1	Q2	Q3	Q4	Q5	Q6	Q7	Q8	Subpop
DEN_DAS5-003490	0.0252	0.0229	0.0256	0.0000	0.5697	0.0000	0.2073	0.1493	EEU
USA_DAS5-004431	0.0382	0.0399	0.1364	0.0000	0.5696	0.0233	0.0486	0.1439	EEU
ARG_DAS5-002153	0.0406	0.0000	0.0507	0.0747	0.5691	0.0025	0.0196	0.2429	EEU
CZE_DAS5-003484	0.0000	0.0000	0.0000	0.0000	0.5637	0.0000	0.4362	0.0000	EEU
ARG_DAS5-002546	0.0000	0.0363	0.0000	0.2217	0.5580	0.0000	0.0000	0.1839	EEU
UNK_DAS5-004200	0.0000	0.0000	0.0976	0.2883	0.5527	0.0000	0.0039	0.0575	EEU
YUG_WATKINS-007061	0.0000	0.0000	0.0000	0.0000	0.5526	0.0000	0.1881	0.2593	EEU
FSV_DAS5-002111	0.0000	0.0010	0.0039	0.0636	0.5515	0.0000	0.2384	0.1415	EEU
BUL_WATKINS-007769	0.0000	0.0000	0.0000	0.0000	0.5511	0.0000	0.4488	0.0000	EEU
SWI_DAS5-004053	0.0734	0.0447	0.0138	0.2181	0.5430	0.0000	0.0160	0.0909	EEU
SWI_DAS5-004047	0.0000	0.0196	0.0122	0.0846	0.5400	0.0218	0.3043	0.0175	EEU
USA_DAS5-LEWJAIN	0.0000	0.0000	0.2261	0.1397	0.5395	0.0000	0.0795	0.0151	EEU
KAZ_DAS5-001436	0.0548	0.0327	0.1622	0.0072	0.5391	0.0125	0.0357	0.1559	EEU
AUS_WATKINS-007777	0.0000	0.0061	0.3619	0.0000	0.5367	0.0126	0.0000	0.0827	EEU
UKR_DAS5-003257	0.0464	0.0000	0.0000	0.2564	0.5345	0.0000	0.1627	0.0000	EEU
GRE_WATKINS-007524	0.0000	0.0033	0.0600	0.0000	0.5257	0.0000	0.2009	0.2100	EEU
CAN_DAS5-001453	0.0049	0.0000	0.3201	0.1385	0.5247	0.0000	0.0117	0.0000	EEU
FSV_DAS5-003553	0.0000	0.0000	0.0000	0.1579	0.5207	0.0000	0.1000	0.2213	EEU
ROM_DAS5-002385	0.0277	0.0000	0.0158	0.3319	0.5204	0.0000	0.1041	0.0000	EEU
FSV_DAS5-002269	0.0000	0.0000	0.0000	0.0000	0.5103	0.0000	0.4897	0.0000	EEU
MOR_WATKINS-007435	0.0000	0.0940	0.0132	0.0000	0.4968	0.0932	0.1362	0.1666	EEU
USA_DAS5-004469	0.0279	0.0040	0.0524	0.0065	0.4946	0.0000	0.4145	0.0000	EEU
AUS_WATKINS-007162	0.0000	0.0000	0.1369	0.3686	0.4944	0.0000	0.0000	0.0000	EEU
SWI_DAS5-004055	0.0340	0.0000	0.1228	0.1178	0.4902	0.0000	0.1245	0.1107	EEU
GEO_PI-572692	0.0000	0.0802	0.0000	0.4297	0.4900	0.0000	0.0000	0.0000	EEU
FSV_DAS5-002649	0.0115	0.0581	0.1669	0.0425	0.4895	0.0097	0.0000	0.2217	EEU
USA_DAS5-004540	0.0390	0.0000	0.0456	0.0774	0.4822	0.0119	0.3439	0.0000	EEU
AUS_DAS5-EMMIT	0.0487	0.0000	0.0335	0.0204	0.4740	0.0000	0.4193	0.0041	EEU
USA_DAS5-004527	0.0386	0.0086	0.2784	0.1300	0.4740	0.0145	0.0560	0.0000	EEU
GER_DAS5-001733	0.0412	0.0000	0.1291	0.0317	0.4734	0.0000	0.1854	0.1393	EEU
IND_DAS5-001047	0.0381	0.0000	0.1303	0.0312	0.4732	0.0000	0.1873	0.1399	EEU
AUS_WATKINS-007084	0.0000	0.0122	0.0513	0.0000	0.4705	0.0000	0.0829	0.3830	EEU
SWI_DAS5-004420	0.0000	0.0000	0.0000	0.0000	0.4669	0.0000	0.4540	0.0790	EEU
FSV_DAS5-002266	0.0403	0.0918	0.0000	0.4018	0.4660	0.0000	0.0000	0.0000	EEU
RUS_Cltr-7635	0.0000	0.0000	0.3673	0.0000	0.4646	0.0000	0.0000	0.1680	EEU
USA_DAS5-004615	0.0336	0.0255	0.0411	0.0524	0.4625	0.0172	0.2512	0.1165	EEU
ROM_DAS5-003128	0.0550	0.0000	0.0208	0.0431	0.4624	0.0000	0.4187	0.0000	EEU
ROM_DAS5-004609	0.0536	0.0000	0.0279	0.0411	0.4557	0.0000	0.4216	0.0000	EEU
KAZ_DAS5-004472	0.0503	0.0000	0.3636	0.0000	0.4514	0.0000	0.0909	0.0438	EEU
GEO_WATKINS-007824	0.0000	0.0000	0.2731	0.0000	0.4501	0.0000	0.0000	0.2767	EEU
BUL_DAS5-003415	0.0828	0.0000	0.0092	0.0639	0.4497	0.0000	0.3944	0.0000	EEU

Accessions	Q1	Q2	Q3	Q4	Q5	Q6	Q7	Q8	Subpop
ARG_DAS5-002594	0.0000	0.0000	0.0183	0.1713	0.4473	0.0000	0.0925	0.2706	EEU
USA_DAS5-004473	0.0172	0.0000	0.2632	0.0257	0.4459	0.0000	0.0683	0.1796	EEU
FSV_DAS5-002477	0.0261	0.0586	0.0000	0.1641	0.4424	0.0000	0.1502	0.1586	EEU
UNK_WATKINS-007446	0.0000	0.0052	0.0000	0.0000	0.4406	0.0068	0.4337	0.1136	EEU
EGY_DAS5-001797	0.0056	0.0131	0.1143	0.1992	0.4404	0.0000	0.0985	0.1289	EEU
SWI_DAS5-004054	0.0607	0.0571	0.0571	0.2583	0.4388	0.0000	0.0000	0.1280	EEU
MAC_DAS5-003783	0.0010	0.0045	0.1507	0.0461	0.4387	0.0000	0.0000	0.3590	EEU
USA_DAS5-001495	0.0000	0.0000	0.1558	0.2964	0.4350	0.0000	0.0277	0.0852	EEU
KEN_DAS5-001378	0.0000	0.0000	0.2071	0.1422	0.4297	0.0000	0.2209	0.0000	EEU
AFG_WATKINS-007108	0.0000	0.0000	0.3759	0.0000	0.4254	0.0000	0.1987	0.0000	EEU
JAP_DAS5-003725	0.0404	0.0000	0.0567	0.2341	0.4242	0.0000	0.0706	0.1740	EEU
USA_DAS5-MDM	0.0225	0.0000	0.3155	0.1365	0.4196	0.0000	0.0753	0.0307	EEU
YUG_WATKINS-007745	0.0741	0.0000	0.0000	0.0371	0.4153	0.0000	0.3233	0.1501	EEU
USA_DAS5-001582	0.0000	0.0129	0.1863	0.3376	0.4129	0.0000	0.0225	0.0278	EEU
RUS_WATKINS-007288	0.0000	0.0000	0.0000	0.0000	0.4114	0.0000	0.3720	0.2165	EEU
RUS_DAS5-002997	0.0179	0.0000	0.3716	0.0000	0.4033	0.0028	0.0000	0.2044	EEU
USA_DAS5-004550	0.0000	0.0022	0.0920	0.1232	0.3970	0.0275	0.3581	0.0000	EEU
KEN_DAS5-003749	0.0048	0.0421	0.1168	0.1450	0.3890	0.0000	0.0616	0.2407	EEU
ARG_DAS5-002570	0.0000	0.0141	0.0000	0.2100	0.3852	0.0000	0.1877	0.2031	EEU
TAI_DAS5-001895	0.0005	0.0000	0.2661	0.1867	0.3839	0.0000	0.1362	0.0266	EEU
SPA_WATKINS-007190	0.0000	0.0181	0.0000	0.0599	0.3770	0.0000	0.1876	0.3574	EEU
SWE_WSC-7-5	0.0000	0.0000	0.1740	0.2545	0.3765	0.0000	0.0000	0.1950	EEU
FSV_DAS5-002075	0.0105	0.0021	0.0934	0.0000	0.3725	0.0000	0.2248	0.2966	EEU
USA_DAS5-001612	0.0545	0.0361	0.0131	0.2152	0.3696	0.0000	0.2093	0.1022	EEU
USA_DAS5-004590	0.0723	0.0000	0.0992	0.2212	0.3664	0.0000	0.2409	0.0000	EEU
AUS_DAS5-002071	0.0000	0.0000	0.0040	0.3326	0.3661	0.0000	0.0000	0.2974	EEU
ARG_DAS5-002176	0.0659	0.0158	0.0080	0.2500	0.3592	0.0000	0.1373	0.1639	EEU
DEN_DAS5-004666	0.0000	0.0147	0.2762	0.0000	0.3564	0.0000	0.2890	0.0638	EEU
USA_DAS5-003063	0.0163	0.1392	0.0000	0.0727	0.3513	0.0699	0.1084	0.2422	EEU
ARG_WATKINS-007065	0.0064	0.0306	0.2660	0.0000	0.3484	0.0045	0.2605	0.0835	EEU
AUS_DAS5-002267	0.0000	0.0210	0.0000	0.2106	0.3435	0.1200	0.0438	0.2611	EEU
USA_DAS5-004408	0.0147	0.0274	0.0133	0.1302	0.3392	0.0151	0.3356	0.1245	EEU
SPA_WATKINS-007518	0.0000	0.2394	0.0000	0.2254	0.3244	0.2108	0.0000	0.0000	EEU
JOR_DAS5-003735	0.0000	0.1257	0.1724	0.2600	0.3229	0.1188	0.0000	0.0002	EEU
TUN_WATKINS-007759	0.0000	0.0000	0.2701	0.1165	0.3105	0.0000	0.0418	0.2612	EEU
IRQ_WATKINS-007715	0.0057	0.0276	0.2181	0.0000	0.3024	0.0000	0.1700	0.2761	EEU
ARG_DAS5-004660	0.0000	0.0645	0.0615	0.2513	0.2823	0.1838	0.0610	0.0955	EEU
FRA_DAS5-002453	0.0000	0.0000	0.0000	0.0000	0.0000	0.0000	0.9999	0.0000	EUMED
FRA_DAS5-004512	0.0000	0.0000	0.0000	0.0000	0.0000	0.0000	0.9999	0.0000	EUMED
YUG_WATKINS-007079	0.0000	0.0000	0.0000	0.0000	0.0000	0.0000	0.9999	0.0000	EUMED
FRA_WATKINS-007316	0.0000	0.0000	0.0000	0.0000	0.0000	0.0000	0.9999	0.0000	EUMED

Accessions	Q1	Q2	Q3	Q4	Q5	Q6	Q7	Q8	Subpop
BEL_DAS5-004343	0.0000	0.0000	0.0000	0.0305	0.0000	0.0000	0.9695	0.0000	EUMED
FSV_DAS5-003149	0.0000	0.0000	0.0000	0.0000	0.0000	0.0000	0.9396	0.0603	EUMED
NET_DAS5-004495	0.0000	0.0000	0.0729	0.0000	0.0000	0.0000	0.9271	0.0000	EUMED
FRA_DAS5-004402	0.0000	0.0000	0.0014	0.0276	0.0351	0.0000	0.9264	0.0095	EUMED
YUG_WATKINS-007295	0.0000	0.0000	0.0000	0.0000	0.1050	0.0000	0.8949	0.0000	EUMED
CHL_DAS5-001008	0.0000	0.0000	0.0000	0.0000	0.1217	0.0000	0.8782	0.0000	EUMED
IND_WATKINS-007585	0.0000	0.0000	0.0000	0.0611	0.0000	0.0000	0.8716	0.0672	EUMED
USA_DAS5-004185	0.0000	0.0000	0.0000	0.0867	0.0439	0.0000	0.8693	0.0000	EUMED
SWE_DAS5-004043	0.0000	0.0000	0.1500	0.0000	0.0000	0.0000	0.8500	0.0000	EUMED
GER_DAS5-004426	0.0000	0.0000	0.0000	0.0000	0.1681	0.0000	0.8319	0.0000	EUMED
GBR_DAS5-004603	0.0000	0.0065	0.0271	0.0435	0.0966	0.0037	0.8226	0.0000	EUMED
USA_DAS5-004167	0.0295	0.0000	0.0000	0.1266	0.0300	0.0000	0.8139	0.0000	EUMED
MAC_DAS5-004268	0.0000	0.0000	0.0060	0.0000	0.1087	0.0000	0.8123	0.0731	EUMED
ZIM_DAS5-001850	0.0370	0.0000	0.0769	0.0599	0.0083	0.0000	0.7922	0.0257	EUMED
IND_WATKINS-007800	0.0000	0.0000	0.0000	0.0003	0.0290	0.0000	0.7868	0.1838	EUMED
BUL_WATKINS-007766	0.0000	0.0000	0.0000	0.0000	0.0492	0.0000	0.7686	0.1821	EUMED
SPA_WATKINS-007445	0.0000	0.0000	0.0000	0.0000	0.0000	0.0042	0.7582	0.2376	EUMED
CHN_WATKINS-007248	0.0000	0.0000	0.0000	0.0000	0.2427	0.0000	0.7573	0.0000	EUMED
CZE_DAS5-004638	0.0000	0.0000	0.0000	0.0000	0.2444	0.0000	0.7556	0.0000	EUMED
GEO_DAS5-003574	0.0000	0.0000	0.0000	0.0000	0.1532	0.0000	0.7530	0.0937	EUMED
BEL_DAS5-004553	0.0733	0.0000	0.0814	0.0000	0.1133	0.0000	0.7319	0.0000	EUMED
MOR_WATKINS-007014	0.0000	0.0000	0.0000	0.0000	0.2660	0.0000	0.7214	0.0125	EUMED
ITA_DAS5-004421	0.0000	0.0000	0.0000	0.0000	0.0000	0.0000	0.7113	0.2886	EUMED
SPA_WATKINS-007451	0.0000	0.0000	0.0000	0.0708	0.1371	0.0113	0.6955	0.0853	EUMED
DEN_DAS5-004333	0.0001	0.0101	0.0129	0.0000	0.2856	0.0000	0.6913	0.0000	EUMED
GER_DAS5-004694	0.0000	0.0000	0.0011	0.0000	0.2831	0.0000	0.6778	0.0380	EUMED
SPA_WATKINS-007643	0.0000	0.0000	0.0000	0.0000	0.0000	0.0000	0.6721	0.3278	EUMED
POR_WATKINS-007500	0.0193	0.0000	0.0042	0.0809	0.1071	0.0000	0.6715	0.1169	EUMED
ROM_DAS5-002781	0.0000	0.0000	0.0127	0.0000	0.3135	0.0039	0.6700	0.0000	EUMED
SPA_WATKINS-007452	0.0000	0.0000	0.0000	0.0000	0.1543	0.0159	0.6684	0.1614	EUMED
TUR_WATKINS-007756	0.0000	0.0000	0.0000	0.0000	0.0000	0.0000	0.6595	0.3404	EUMED
AUT_DAS5-003346	0.0000	0.0000	0.0000	0.0000	0.3437	0.0000	0.6562	0.0000	EUMED
BOS_DAS5-003394	0.0000	0.0000	0.0031	0.1136	0.1180	0.0000	0.6452	0.1200	EUMED
GBR_DAS5-004156	0.0348	0.0000	0.0000	0.2604	0.0597	0.0000	0.6450	0.0000	EUMED
TUR_WSC-8-6	0.2003	0.0000	0.1557	0.0006	0.0000	0.0000	0.6434	0.0000	EUMED
GBR_DAS5-004425	0.0099	0.0061	0.0000	0.0000	0.2769	0.0000	0.6433	0.0638	EUMED
FSV_DAS5-003084	0.0581	0.0336	0.2129	0.0411	0.0063	0.0050	0.6431	0.0000	EUMED
NEW_DAS5-004328	0.0000	0.0486	0.0000	0.0768	0.0285	0.0442	0.6431	0.1589	EUMED
MOR_WATKINS-007651	0.0000	0.0000	0.0000	0.0000	0.0000	0.0000	0.6422	0.3578	EUMED
FSV_DAS5-002992	0.0000	0.0000	0.0000	0.0256	0.2502	0.0000	0.6359	0.0883	EUMED
POL_DAS5-002864	0.0000	0.0000	0.0000	0.0000	0.3665	0.0000	0.6334	0.0000	EUMED

Accessions	Q1	Q2	Q3	Q4	Q5	Q6	Q7	Q8	Subpop
USA_DAS5-004571	0.0000	0.0000	0.0000	0.0248	0.3434	0.0000	0.6318	0.0000	EUMED
POR_DAS5-001493	0.0000	0.0000	0.0000	0.0000	0.0000	0.0000	0.6285	0.3715	EUMED
JAP_DAS5-003731	0.0210	0.0000	0.2351	0.0000	0.1192	0.0000	0.6246	0.0000	EUMED
MAC_DAS5-003778	0.0000	0.0000	0.0274	0.0000	0.1670	0.0000	0.6223	0.1832	EUMED
POL_DAS5-003915	0.0090	0.0000	0.0366	0.0000	0.3268	0.0059	0.6217	0.0000	EUMED
NEW_DAS5-004587	0.0000	0.1545	0.0421	0.0000	0.0101	0.0089	0.6215	0.1630	EUMED
ITA_DAS5-003724	0.0238	0.0457	0.0000	0.0357	0.1886	0.0000	0.6166	0.0896	EUMED
SPA_WATKINS-007572	0.0000	0.0016	0.0000	0.0000	0.0997	0.0000	0.6138	0.2849	EUMED
CAN_DAS5-004364	0.0000	0.0000	0.0000	0.0000	0.3746	0.0000	0.6138	0.0116	EUMED
BEL_DAS5-003357	0.0000	0.0000	0.3449	0.0499	0.0000	0.0000	0.6052	0.0000	EUMED
CZE_DAS5-004468	0.0017	0.0000	0.0186	0.0384	0.3375	0.0000	0.6037	0.0000	EUMED
GRE_DAS5-003599	0.0869	0.0143	0.0681	0.1905	0.0339	0.0000	0.6030	0.0033	EUMED
USA_DAS5-003074	0.0000	0.0000	0.0000	0.0840	0.3201	0.0000	0.5959	0.0000	EUMED
MEX_WATKINS-007412	0.0319	0.0000	0.3203	0.0000	0.0000	0.0000	0.5909	0.0569	EUMED
AUS_DAS5-HILL-81	0.0010	0.0000	0.0380	0.1200	0.2502	0.0000	0.5907	0.0000	EUMED
ROM_DAS5-003943	0.0165	0.0000	0.0004	0.0000	0.4050	0.0000	0.5782	0.0000	EUMED
OMA_DAS5-001134	0.0158	0.0000	0.0000	0.3909	0.0172	0.0000	0.5760	0.0000	EUMED
USA_DAS5-001756	0.0000	0.0000	0.0000	0.2380	0.0822	0.0000	0.5739	0.1059	EUMED
IRQ_WATKINS-007691	0.0063	0.0000	0.0174	0.0288	0.0389	0.0000	0.5706	0.3381	EUMED
DEN_DAS5-003494	0.0015	0.0000	0.0031	0.0034	0.3843	0.0000	0.5703	0.0373	EUMED
POR_DAS5-002362	0.0000	0.0000	0.0000	0.0000	0.1206	0.0103	0.5699	0.2992	EUMED
CRO_DAS5-003477	0.0308	0.0000	0.0807	0.2342	0.0854	0.0000	0.5689	0.0000	EUMED
POL_DAS5-002998	0.0265	0.1790	0.0000	0.0000	0.1363	0.0961	0.5621	0.0000	EUMED
ROM_DAS5-002299	0.0768	0.0000	0.0404	0.0000	0.3215	0.0000	0.5613	0.0000	EUMED
IND_WATKINS-007395	0.0000	0.0000	0.4402	0.0000	0.0000	0.0000	0.5597	0.0000	EUMED
USA_DAS5-004178	0.0014	0.0013	0.0000	0.0114	0.4061	0.0000	0.5588	0.0209	EUMED
ITA_DAS5-003721	0.1845	0.0000	0.2583	0.0000	0.0000	0.0000	0.5572	0.0000	EUMED
USA_DAS5-004166	0.0296	0.0000	0.0083	0.3434	0.0000	0.0084	0.5518	0.0585	EUMED
CHN_WATKINS-007212	0.0240	0.2797	0.0286	0.0340	0.0261	0.0168	0.5500	0.0409	EUMED
BUL_DAS5-002857	0.0975	0.0201	0.0596	0.0450	0.2309	0.0016	0.5453	0.0000	EUMED
MEX_DAS5-CARA	0.0096	0.0000	0.0674	0.0515	0.3379	0.0000	0.5335	0.0000	EUMED
CHN_WATKINS-007244	0.0000	0.0000	0.0000	0.0236	0.4425	0.0000	0.5326	0.0013	EUMED
RUS_DAS5-002228	0.0000	0.0000	0.0353	0.0191	0.4138	0.0000	0.5318	0.0000	EUMED
CHN_WATKINS-007260	0.0105	0.0000	0.3175	0.0237	0.1194	0.0000	0.5289	0.0000	EUMED
KYR_DAS5-004555	0.0000	0.0173	0.0000	0.0000	0.3848	0.0206	0.5265	0.0508	EUMED
CAN_WATKINS-007678	0.0189	0.0000	0.0439	0.0031	0.0050	0.0000	0.5259	0.4031	EUMED
AUT_DAS5-004389	0.0059	0.0000	0.0000	0.0000	0.4721	0.0000	0.5219	0.0000	EUMED
CHN_WATKINS-007272	0.0529	0.0000	0.3307	0.0123	0.0840	0.0000	0.5202	0.0000	EUMED
JAP_DAS5-003729	0.0000	0.0000	0.0000	0.0000	0.4398	0.0000	0.5152	0.0449	EUMED
DEN_DAS5-003493	0.0348	0.0195	0.0712	0.0141	0.1530	0.0000	0.5147	0.1926	EUMED
SPA_WATKINS-007449	0.0000	0.0000	0.0000	0.0000	0.0948	0.0000	0.5145	0.3907	EUMED

Accessions	Q1	Q2	Q3	Q4	Q5	Q6	Q7	Q8	Subpop
BEL_DAS5-004342	0.0066	0.0118	0.0259	0.0018	0.4140	0.0000	0.5129	0.0270	EUMED
GUA_DAS5-001503	0.0309	0.0009	0.0229	0.0963	0.1516	0.0000	0.5102	0.1871	EUMED
BUR_DAS5-001036	0.2168	0.0000	0.0000	0.2482	0.0000	0.0000	0.5069	0.0280	EUMED
AUS_WATKINS-007285	0.0000	0.0000	0.0000	0.0000	0.4395	0.0000	0.5028	0.0576	EUMED
CHN_DAS5-001672	0.0941	0.0000	0.0045	0.4032	0.0000	0.0000	0.4981	0.0000	EUMED
USA_DAS5-004165	0.0459	0.0000	0.0000	0.2774	0.1803	0.0000	0.4964	0.0000	EUMED
ROM_DAS5-003046	0.0256	0.0000	0.0000	0.2488	0.2310	0.0000	0.4946	0.0000	EUMED
CHL_DAS5-004288	0.0000	0.1696	0.0000	0.0056	0.1778	0.0000	0.4936	0.1534	EUMED
ARG_DAS5-002563	0.0000	0.0000	0.1868	0.0996	0.0670	0.0000	0.4836	0.1629	EUMED
BOS_DAS5-003393	0.0004	0.0000	0.0468	0.0000	0.3424	0.0000	0.4836	0.1267	EUMED
MOR_DAS5-002766	0.0000	0.0000	0.1754	0.1069	0.2444	0.0000	0.4733	0.0000	EUMED
AUS_DAS5-AVA	0.0588	0.0000	0.0159	0.0000	0.4522	0.0000	0.4730	0.0000	EUMED
PHI_DAS5-001051	0.3054	0.0000	0.1066	0.1208	0.0000	0.0000	0.4672	0.0000	EUMED
SPA_WATKINS-007684	0.0551	0.0000	0.0000	0.1956	0.0666	0.0100	0.4646	0.2080	EUMED
URU_DAS5-004229	0.0112	0.0611	0.0095	0.0036	0.0062	0.0075	0.4644	0.4365	EUMED
ITA_WATKINS-007423	0.0090	0.0000	0.0079	0.0000	0.1099	0.0000	0.4618	0.4113	EUMED
CAN_DAS5-001843	0.0480	0.0000	0.0100	0.2947	0.1132	0.0000	0.4596	0.0745	EUMED
ARG_DAS5-002370	0.0149	0.0614	0.0121	0.0034	0.0165	0.0066	0.4541	0.4310	EUMED
URU_DAS5-002852	0.0554	0.0086	0.0176	0.2292	0.0201	0.0000	0.4512	0.2180	EUMED
USA_DAS5-004310	0.0000	0.0000	0.0000	0.0539	0.4081	0.0000	0.4464	0.0916	EUMED
BUL_DAS5-003418	0.0313	0.0000	0.0000	0.1604	0.3646	0.0000	0.4436	0.0000	EUMED
ARG_DAS5-003070	0.0741	0.0000	0.0000	0.2174	0.1365	0.1285	0.4434	0.0000	EUMED
GRE_DAS5-004545	0.0286	0.0000	0.0479	0.0017	0.2049	0.0000	0.4349	0.2819	EUMED
AFG_WATKINS-007048	0.0000	0.0000	0.0905	0.0000	0.1601	0.0000	0.4255	0.3239	EUMED
ZAM_DAS5-001491	0.1650	0.0000	0.0255	0.3250	0.0000	0.0000	0.4236	0.0609	EUMED
USA_DAS5-004161	0.0869	0.0000	0.0000	0.3903	0.1001	0.0000	0.4227	0.0000	EUMED
BRA_DAS5-003410	0.0847	0.0000	0.1082	0.1596	0.0449	0.0000	0.4154	0.1872	EUMED
ARG_DAS5-001508	0.1295	0.0000	0.0000	0.2222	0.2341	0.0000	0.4142	0.0000	EUMED
MEX_WATKINS-007199	0.0000	0.0000	0.1442	0.0000	0.3356	0.0000	0.4134	0.1068	EUMED
USA_DAS5-004557	0.0722	0.0000	0.0555	0.0000	0.3129	0.0000	0.4131	0.1462	EUMED
USA_DAS5-002311	0.0446	0.0056	0.0622	0.0877	0.3902	0.0000	0.4096	0.0000	EUMED
ANG_DAS5-001610	0.1627	0.0000	0.0000	0.3894	0.0394	0.0000	0.4085	0.0000	EUMED
ARG_DAS5-002567	0.0525	0.0000	0.1095	0.1341	0.0625	0.0000	0.3989	0.2426	EUMED
CRO_DAS5-004640	0.0408	0.0000	0.1155	0.2945	0.1646	0.0000	0.3846	0.0000	EUMED
URU_DAS5-004212	0.0000	0.0000	0.0541	0.2300	0.0099	0.0000	0.3744	0.3316	EUMED
BUL_DAS5-003423	0.0409	0.0000	0.0116	0.2320	0.3442	0.0000	0.3712	0.0000	EUMED
BEL_DAS5-004670	0.0000	0.0000	0.0469	0.0830	0.2870	0.0000	0.3650	0.2180	EUMED
URU_DAS5-004217	0.0373	0.0034	0.0863	0.2369	0.0056	0.0029	0.3349	0.2926	EUMED
GBR_DAS5-004155	0.0713	0.2711	0.0707	0.0855	0.0152	0.0403	0.3130	0.1330	EUMED
IND_WATKINS-007339	0.0051	0.0000	0.2448	0.0647	0.1281	0.0000	0.3086	0.2486	EUMED
BEL_DAS5-003370	0.0649	0.1633	0.0809	0.0426	0.1134	0.0000	0.3032	0.2317	EUMED

Accessions	Q1	Q2	Q3	Q4	Q5	Q6	Q7	Q8	Subpop
KOR_DAS5-002505	0.0000	0.0000	0.2752	0.0000	0.2866	0.0000	0.3022	0.1360	EUMED
PER_DAS5-003903	0.0313	0.0693	0.1639	0.2886	0.1412	0.0053	0.3004	0.0000	EUMED
BOS_DAS5-004665	0.0229	0.0000	0.1416	0.1689	0.1436	0.0030	0.2789	0.2411	EUMED
ITA_DAS5-001396	0.0944	0.0038	0.2213	0.1942	0.0937	0.0077	0.2611	0.1238	EUMED
SPA_DAS5-004018	0.0715	0.2340	0.1123	0.0385	0.0160	0.0181	0.2581	0.2514	EUMED
BEL_DAS5-004418	0.0731	0.1302	0.1600	0.0000	0.1776	0.0000	0.2518	0.2073	EUMED
USA_DAS5-004691	0.0511	0.1679	0.1878	0.0000	0.1928	0.0000	0.2510	0.1494	EUMED
SER_DAS5-003993	0.0723	0.1690	0.1655	0.0266	0.1991	0.0000	0.2403	0.1272	EUMED
KEN_DAS5-001271	0.0522	0.0075	0.2230	0.2082	0.0993	0.0047	0.2252	0.1799	EUMED
TUR_DAS5-002287	0.0000	0.9999	0.0000	0.0000	0.0000	0.0000	0.0000	0.0000	FSV
FSV_DAS5-002301	0.0000	0.9999	0.0000	0.0000	0.0000	0.0000	0.0000	0.0000	FSV
FSV_DAS5-002303	0.0000	0.9999	0.0000	0.0000	0.0000	0.0000	0.0000	0.0000	FSV
FSV_DAS5-002449	0.0000	0.9999	0.0000	0.0000	0.0000	0.0000	0.0000	0.0000	FSV
FSV_DAS5-002471	0.0000	0.9999	0.0000	0.0000	0.0000	0.0000	0.0000	0.0000	FSV
FSV_DAS5-002515	0.0000	0.9999	0.0000	0.0000	0.0000	0.0000	0.0000	0.0000	FSV
FSV_DAS5-002631	0.0000	0.9999	0.0000	0.0000	0.0000	0.0000	0.0000	0.0000	FSV
ITA_DAS5-002933	0.0000	0.9999	0.0000	0.0000	0.0000	0.0000	0.0000	0.0000	FSV
FSV_DAS5-002961	0.0000	0.9999	0.0000	0.0000	0.0000	0.0000	0.0000	0.0000	FSV
FSV_DAS5-002978	0.0000	0.9999	0.0000	0.0000	0.0000	0.0000	0.0000	0.0000	FSV
FSV_DAS5-003094	0.0000	0.9999	0.0000	0.0000	0.0000	0.0000	0.0000	0.0000	FSV
AUS_DAS5-000296	0.0000	0.9999	0.0000	0.0000	0.0000	0.0000	0.0000	0.0000	FSV
AZE_DAS5-002735	0.0000	0.9999	0.0000	0.0000	0.0000	0.0000	0.0000	0.0000	FSV
LEB_DAS5-002454	0.0000	0.9999	0.0000	0.0000	0.0000	0.0000	0.0000	0.0000	FSV
POL_DAS5-002338	0.0000	0.9871	0.0000	0.0000	0.0000	0.0128	0.0000	0.0000	FSV
FSV_DAS5-002236	0.0000	0.9686	0.0000	0.0000	0.0000	0.0314	0.0000	0.0000	FSV
GER_DAS5-002790	0.0000	0.9644	0.0000	0.0000	0.0000	0.0355	0.0000	0.0000	FSV
POR_DAS5-002406	0.0000	0.9432	0.0000	0.0000	0.0000	0.0567	0.0000	0.0000	FSV
UNK_DAS5-003033	0.0000	0.9333	0.0093	0.0000	0.0000	0.0354	0.0000	0.0221	FSV
FSV_DAS5-002519	0.0000	0.9287	0.0000	0.0000	0.0000	0.0712	0.0000	0.0000	FSV
FSV_DAS5-003258	0.0000	0.9281	0.0000	0.0000	0.0000	0.0000	0.0000	0.0718	FSV
FSV_DAS5-003142	0.0000	0.9212	0.0000	0.0000	0.0000	0.0787	0.0000	0.0000	FSV
ALG_DAS5-002458	0.0000	0.8832	0.0000	0.0000	0.0000	0.1167	0.0000	0.0000	FSV
IND_DAS5-002138	0.0000	0.8729	0.1096	0.0174	0.0000	0.0000	0.0000	0.0000	FSV
FSV_DAS5-002996	0.0000	0.8629	0.0223	0.0050	0.0000	0.0000	0.0828	0.0270	FSV
ITA_DAS5-002212	0.0000	0.8453	0.0000	0.0000	0.0000	0.1547	0.0000	0.0000	FSV
MON_DAS5-004285	0.0000	0.8299	0.0000	0.0000	0.0000	0.0000	0.0748	0.0953	FSV
ITA_DAS5-002199	0.0000	0.8091	0.0000	0.0000	0.0000	0.0000	0.0600	0.1309	FSV
AZE_DAS5-003041	0.0090	0.7971	0.0078	0.0000	0.0808	0.1053	0.0000	0.0000	FSV
FSV_DAS5-002765	0.0000	0.7956	0.0583	0.0000	0.0000	0.0000	0.0000	0.1461	FSV
AUS_DAS5-000425	0.0399	0.7782	0.0000	0.0000	0.0000	0.0000	0.1067	0.0751	FSV
ITA_DAS5-002425	0.0000	0.7222	0.0000	0.0000	0.0000	0.2777	0.0000	0.0000	FSV

Accessions	Q1	Q2	Q3	Q4	Q5	Q6	Q7	Q8	Subpop
FSV_DAS5-002799	0.0000	0.6904	0.0000	0.0000	0.3095	0.0000	0.0000	0.0000	FSV
FSV_DAS5-002740	0.0000	0.6744	0.0000	0.0000	0.3255	0.0000	0.0000	0.0000	FSV
SPA_DAS5-002916	0.0000	0.6640	0.0000	0.0000	0.0390	0.1658	0.0419	0.0893	FSV
GER_WATKINS-007811	0.0000	0.6349	0.0861	0.0000	0.0000	0.2790	0.0000	0.0000	FSV
YEM_DAS5-004244	0.0175	0.6154	0.2016	0.0622	0.0000	0.0663	0.0370	0.0000	FSV
ETH_DAS5-001932	0.0002	0.5840	0.3128	0.0088	0.0616	0.0000	0.0326	0.0000	FSV
FSV_DAS5-002986	0.0000	0.5781	0.0626	0.0095	0.0738	0.2250	0.0511	0.0000	FSV
SPA_DAS5-004007	0.0424	0.5631	0.0788	0.0299	0.0000	0.0000	0.2858	0.0000	FSV
IND_DAS5-002802	0.0232	0.5563	0.2608	0.0569	0.0571	0.0000	0.0457	0.0000	FSV
MEX_DAS5-001242	0.0000	0.0000	0.0000	0.0000	0.0000	0.0000	0.0000	0.9999	MED
ALG_WATKINS-007122	0.0000	0.0000	0.0000	0.0000	0.0000	0.0000	0.0000	0.9999	MED
CAN_WATKINS-007650	0.0000	0.0000	0.0000	0.0000	0.0000	0.0000	0.0000	0.9999	MED
SPA_WATKINS-007443	0.0000	0.0000	0.0000	0.0000	0.0000	0.0000	0.0000	0.9999	MED
ALG_DAS5-001609	0.0000	0.0000	0.0000	0.0177	0.0000	0.0000	0.0000	0.9822	MED
TUN_WATKINS-007035	0.0000	0.0000	0.0000	0.0000	0.0000	0.0000	0.0408	0.9591	MED
AFG_WATKINS-007117	0.0000	0.0000	0.0041	0.0223	0.0000	0.0000	0.0228	0.9508	MED
ISR_DAS5-003704	0.0000	0.0118	0.0167	0.0000	0.0000	0.0278	0.0000	0.9437	MED
AUS_WATKINS-007089	0.0000	0.0000	0.0000	0.0000	0.0000	0.0000	0.0742	0.9257	MED
PRK_WSC-8-1	0.0000	0.0318	0.0000	0.0000	0.0000	0.0000	0.0494	0.9188	MED
TUR_WATKINS-007030	0.0000	0.0058	0.0375	0.0000	0.0778	0.0000	0.0000	0.8789	MED
POR_DAS5-002049	0.0000	0.0631	0.0000	0.0000	0.0000	0.0000	0.0603	0.8765	MED
ISR_DAS5-001203	0.0000	0.0543	0.0000	0.0000	0.0000	0.0793	0.0000	0.8663	MED
FSV_DAS5-002447	0.0000	0.0000	0.0158	0.0428	0.0000	0.0237	0.0553	0.8624	MED
SPA_WATKINS-007127	0.0008	0.0000	0.0167	0.0000	0.0359	0.0000	0.0896	0.8570	MED
GRE_WATKINS-007090	0.0000	0.0614	0.0000	0.0000	0.0000	0.0877	0.0000	0.8508	MED
SPA_WATKINS-007187	0.0116	0.0794	0.0386	0.0000	0.0195	0.0086	0.0000	0.8422	MED
MOR_DAS5-003835	0.0000	0.0000	0.1226	0.0268	0.0088	0.0000	0.0000	0.8417	MED
SYR_WATKINS-007009	0.0000	0.0000	0.0892	0.0000	0.0628	0.0149	0.0000	0.8331	MED
CYP_WATKINS-007826	0.0000	0.0126	0.1196	0.0000	0.0485	0.0000	0.0000	0.8192	MED
CYP_WSC-7-4	0.0000	0.0000	0.0472	0.0000	0.0018	0.1396	0.0000	0.8113	MED
SPA_WATKINS-007188	0.0631	0.0723	0.0043	0.0000	0.0000	0.0000	0.0579	0.8024	MED
SPA_DAS5-004012	0.0000	0.0000	0.0000	0.0000	0.1497	0.0020	0.0472	0.8011	MED
MEX_Cltr-2804	0.0102	0.0164	0.0065	0.0187	0.0208	0.0000	0.1272	0.8001	MED
KEN_DAS5-001809	0.0000	0.1162	0.0079	0.0000	0.0000	0.0692	0.0108	0.7959	MED
SPA_DAS5-004010	0.0000	0.0307	0.0130	0.0000	0.1209	0.0000	0.0477	0.7877	MED
POR_DAS5-002104	0.0000	0.0434	0.0000	0.0139	0.0398	0.0000	0.1236	0.7793	MED
GRE_DAS5-001641	0.0112	0.0000	0.0000	0.0439	0.0765	0.0932	0.0000	0.7752	MED
CYP_DAS5-003480	0.0185	0.0000	0.1060	0.0000	0.0626	0.0000	0.0397	0.7732	MED
IND_WATKINS-007561	0.0321	0.0000	0.0017	0.0000	0.1429	0.0000	0.0752	0.7480	MED
GRE_WSC-7-3	0.0000	0.0217	0.0000	0.0000	0.0326	0.0000	0.2069	0.7386	MED
SPA_WATKINS-007558	0.0000	0.0000	0.0000	0.0000	0.0947	0.0000	0.1741	0.7312	MED

Accessions	Q1	Q2	Q3	Q4	Q5	Q6	Q7	Q8	Subpop
MOR_DAS5-004265	0.0000	0.0321	0.2282	0.0000	0.0088	0.0000	0.0000	0.7309	MED
SPA_WATKINS-007677	0.0000	0.0000	0.0000	0.0000	0.1529	0.0000	0.1231	0.7240	MED
POR_DAS5-001924	0.0000	0.0851	0.0000	0.0000	0.0000	0.0000	0.1959	0.7189	MED
AUS_WATKINS-007145	0.0000	0.0000	0.0000	0.1854	0.0070	0.0000	0.1012	0.7064	MED
SPA_DAS5-004020	0.0000	0.0226	0.0000	0.0000	0.0000	0.0000	0.2723	0.7051	MED
AUS_WATKINS-007299	0.0000	0.0000	0.0000	0.0000	0.2162	0.0000	0.0879	0.6959	MED
SPA_DAS5-001212	0.0196	0.1264	0.0243	0.0000	0.0000	0.0010	0.1358	0.6929	MED
URU_PI-43355	0.0011	0.0063	0.0040	0.0000	0.0000	0.0000	0.3251	0.6635	MED
ECU_DAS5-003501	0.0157	0.1256	0.0585	0.0054	0.0000	0.1338	0.0000	0.6611	MED
BRA_WSC-8-9	0.0000	0.0000	0.0000	0.0360	0.0000	0.0000	0.3029	0.6610	MED
GRE_WATKINS-007077	0.0000	0.0110	0.0000	0.0263	0.0705	0.0000	0.2361	0.6561	MED
IND_WATKINS-007802	0.0014	0.0015	0.0052	0.0000	0.0000	0.0000	0.3392	0.6527	MED
POL_DAS5-002870	0.0000	0.0000	0.0000	0.0000	0.0000	0.0000	0.3506	0.6494	MED
GRE_WATKINS-007001	0.0000	0.0142	0.0000	0.0000	0.0000	0.0022	0.3358	0.6477	MED
IRQ_WATKINS-007638	0.0270	0.0105	0.0655	0.0000	0.0000	0.0000	0.2505	0.6465	MED
AUS_WATKINS-007156	0.0000	0.0000	0.0000	0.0170	0.0415	0.0000	0.2966	0.6449	MED
EGY_WATKINS-007542	0.0000	0.0000	0.0000	0.0727	0.1378	0.0000	0.1473	0.6421	MED
TUN_DAS5-001935	0.0000	0.0135	0.1086	0.0000	0.1247	0.0358	0.0764	0.6411	MED
SPA_WATKINS-007455	0.0000	0.0000	0.0000	0.0000	0.0294	0.0000	0.3314	0.6392	MED
AUS_WATKINS-007623	0.0000	0.0000	0.0000	0.0916	0.0000	0.0000	0.2726	0.6357	MED
GRE_WATKINS-007002	0.0000	0.0000	0.0731	0.0984	0.0977	0.0000	0.0952	0.6355	MED
POR_DAS5-003928	0.0321	0.2447	0.0067	0.0000	0.0000	0.0000	0.0860	0.6305	MED
GRE_WATKINS-007093	0.0000	0.0000	0.0000	0.0000	0.0000	0.0000	0.3731	0.6269	MED
POR_WSC-8-2	0.0182	0.0000	0.0000	0.0067	0.0000	0.0000	0.3513	0.6238	MED
FSV_DAS5-003176	0.0000	0.0000	0.0000	0.0000	0.1184	0.0000	0.2707	0.6109	MED
MOR_DAS5-004663	0.0000	0.0570	0.0941	0.0000	0.1772	0.0615	0.0000	0.6101	MED
POR_DAS5-003925	0.0000	0.1257	0.0000	0.0000	0.0000	0.0000	0.2674	0.6068	MED
ANG_WSC-8-8	0.0000	0.0049	0.0335	0.2411	0.0000	0.0000	0.1281	0.5924	MED
PER_DAS5-004698	0.0000	0.1343	0.0000	0.0000	0.0000	0.2119	0.0849	0.5689	MED
TUN_WATKINS-007799	0.0000	0.0283	0.0991	0.0642	0.0000	0.0893	0.1517	0.5674	MED
ARG_WATKINS-007070	0.0084	0.1049	0.0081	0.0074	0.1699	0.0481	0.0864	0.5669	MED
ANG_DAS5-001276	0.0000	0.0140	0.0420	0.2327	0.0000	0.0000	0.1500	0.5613	MED
GUA_WSC-8-3	0.0078	0.0560	0.0038	0.0000	0.0909	0.0000	0.2857	0.5558	MED
CRO_DAS5-004316	0.0000	0.0000	0.0000	0.0000	0.2661	0.0000	0.1808	0.5531	MED
TUR_WSC-8-4	0.0000	0.0504	0.2531	0.0000	0.1020	0.0466	0.0000	0.5479	MED
AUS_WATKINS-007148	0.0000	0.0000	0.0254	0.0000	0.2915	0.0000	0.1452	0.5379	MED
ARG_DAS5-003296	0.0365	0.0550	0.0000	0.1375	0.1242	0.1113	0.0000	0.5355	MED
GRE_WATKINS-007088	0.0000	0.0000	0.0000	0.0000	0.0457	0.0000	0.4234	0.5308	MED
AUS_WATKINS-007056	0.0000	0.0244	0.0212	0.0000	0.2401	0.0042	0.1821	0.5280	MED
GUA_DAS5-003601	0.0342	0.1906	0.0832	0.0007	0.0266	0.0000	0.1442	0.5205	MED
BRA_DAS5-003134	0.0646	0.1871	0.0197	0.0313	0.0520	0.0121	0.1146	0.5185	MED

Accessions	Q1	Q2	Q3	Q4	Q5	Q6	Q7	Q8	Subpop
CYP_DAS5-001320	0.0295	0.0553	0.0499	0.1108	0.0466	0.0000	0.1922	0.5156	MED
POR_WATKINS-007494	0.0620	0.0000	0.0792	0.0000	0.0000	0.0000	0.3435	0.5152	MED
SPA_WATKINS-007520	0.0000	0.0000	0.0000	0.0000	0.2300	0.0000	0.2566	0.5133	MED
ZAF_DAS5-002317	0.0000	0.0000	0.0000	0.2597	0.0000	0.0000	0.2277	0.5125	MED
GUA_DAS5-004664	0.0187	0.0492	0.1056	0.0837	0.0980	0.0000	0.1337	0.5111	MED
IRQ_WATKINS-007641	0.0000	0.0000	0.0000	0.1773	0.2678	0.0000	0.0462	0.5087	MED
NEW_DAS5-002463	0.0371	0.1609	0.0328	0.0068	0.0551	0.0000	0.2016	0.5058	MED
POR_WATKINS-007497	0.0212	0.2366	0.0067	0.0013	0.0027	0.0485	0.1827	0.5004	MED
MOR_DAS5-003849	0.0000	0.0089	0.0393	0.1217	0.0000	0.0023	0.3354	0.4924	MED
FSV_DAS5-002708	0.0052	0.0129	0.0000	0.3437	0.0963	0.0088	0.0442	0.4889	MED
TUR_DAS5-004483	0.0000	0.0598	0.2425	0.0000	0.1064	0.1033	0.0000	0.4881	MED
USA_DAS5-001731	0.0504	0.0266	0.0179	0.0019	0.1866	0.0000	0.2301	0.4865	MED
POR_DAS5-004676	0.0381	0.2750	0.0077	0.0000	0.0000	0.0314	0.1643	0.4836	MED
FSV_DAS5-002981	0.0000	0.0000	0.0000	0.2536	0.2315	0.0000	0.0328	0.4821	MED
LEB_DAS5-004337	0.0000	0.0005	0.0000	0.0831	0.0260	0.0000	0.4111	0.4793	MED
URU_DAS5-001755	0.0000	0.0000	0.0864	0.1466	0.1501	0.0000	0.1437	0.4732	MED
TUN_DAS5-001399	0.0000	0.0775	0.0352	0.1553	0.1749	0.0860	0.0000	0.4711	MED
URU_DAS5-001042	0.0132	0.0000	0.0656	0.0674	0.0000	0.0000	0.3836	0.4703	MED
ALG_DAS5-004460	0.0000	0.0351	0.4548	0.0000	0.0000	0.0457	0.0000	0.4643	MED
ITA_DAS5-004695	0.0000	0.0120	0.0000	0.0725	0.0165	0.0000	0.4408	0.4581	MED
FSV_DAS5-002165	0.0000	0.0000	0.0000	0.1287	0.0365	0.0000	0.3772	0.4576	MED
FSV_DAS5-002351	0.0000	0.0000	0.0000	0.0000	0.2139	0.0000	0.3324	0.4537	MED
SPA_WATKINS-007430	0.0000	0.0000	0.0481	0.0000	0.1778	0.0000	0.3220	0.4521	MED
SYR_DAS5-004059	0.0000	0.0000	0.3276	0.0000	0.2140	0.0069	0.0000	0.4515	MED
CHN_WATKINS-007261	0.0077	0.0051	0.1507	0.0291	0.0918	0.0000	0.2698	0.4457	MED
CHN_WATKINS-007202	0.0000	0.0000	0.0000	0.2108	0.0331	0.0000	0.3131	0.4430	MED
ARG_DAS5-002171	0.0000	0.0000	0.0000	0.1603	0.1346	0.0000	0.2626	0.4424	MED
IND_WATKINS-007388	0.0000	0.0000	0.3383	0.0685	0.1144	0.0000	0.0493	0.4295	MED
ZIM_WATKINS-007692	0.0000	0.0000	0.3209	0.0000	0.1142	0.0000	0.1584	0.4065	MED
MEX_WATKINS-007221	0.0000	0.0260	0.0631	0.1164	0.1315	0.0914	0.1667	0.4050	MED
SWE_DAS5-004036	0.0306	0.0590	0.0256	0.0254	0.0422	0.0294	0.3901	0.3977	MED
IRN_DAS5-003670	0.0000	0.0000	0.3080	0.0000	0.3031	0.0000	0.0000	0.3889	MED
ZAF_DAS5-004657	0.0000	0.0000	0.0418	0.2871	0.0899	0.0000	0.1964	0.3848	MED
UKR_DAS5-001791	0.0568	0.0000	0.0915	0.0133	0.3319	0.0000	0.1242	0.3824	MED
IRQ_WSC-7-2	0.0000	0.0000	0.4007	0.0000	0.2188	0.0000	0.0000	0.3804	MED
POR_DAS5-001415	0.0000	0.0000	0.0000	0.0000	0.0000	0.0000	0.6214	0.3785	MED
SPA_DAS5-004493	0.0000	0.0341	0.0453	0.0000	0.1846	0.0000	0.3574	0.3785	MED
IRQ_WATKINS-007640	0.0000	0.0306	0.3609	0.0378	0.1733	0.0134	0.0077	0.3762	MED
CRO_DAS5-003478	0.0000	0.0062	0.0370	0.0000	0.2423	0.0221	0.3163	0.3762	MED
BOS_DAS5-003388	0.0056	0.0000	0.0039	0.0210	0.2275	0.0023	0.3660	0.3737	MED
NOR_DAS5-001859	0.0063	0.0035	0.1372	0.2032	0.2692	0.0085	0.0000	0.3720	MED

Accessions	Q1	Q2	Q3	Q4	Q5	Q6	Q7	Q8	Subpop
ARG_DAS5-002565	0.1155	0.0058	0.0000	0.3026	0.1761	0.0000	0.0310	0.3690	MED
NET_DAS5-001291	0.0367	0.1191	0.0071	0.0868	0.2591	0.0000	0.1284	0.3628	MED
PER_DAS5-003904	0.0227	0.0000	0.1440	0.2604	0.0000	0.0000	0.2161	0.3569	MED
JAP_DAS5-003726	0.0000	0.1358	0.0000	0.0894	0.2562	0.1369	0.0275	0.3542	MED
AUS_WATKINS-007017	0.0131	0.0137	0.1786	0.1073	0.0000	0.0055	0.3290	0.3528	MED
ARG_DAS5-002568	0.0000	0.0513	0.0000	0.1489	0.2598	0.0554	0.1411	0.3435	MED
ARG_DAS5-001845	0.0000	0.0550	0.0478	0.2282	0.1661	0.1696	0.0000	0.3333	MED
AUS_DAS5-001910	0.0248	0.0312	0.1717	0.2537	0.1883	0.0000	0.0004	0.3299	MED
CHN_WATKINS-007246	0.0006	0.0007	0.1502	0.0104	0.2666	0.0192	0.2370	0.3153	MED
ROM_DAS5-002087	0.0000	0.0000	0.1822	0.2482	0.0827	0.0064	0.1816	0.2990	MED
FSV_DAS5-002700	0.0918	0.0328	0.2076	0.1277	0.0543	0.0434	0.1623	0.2802	MED
PAK_Cltr-15134	0.0000	0.0000	0.9999	0.0000	0.0000	0.0000	0.0000	0.0000	OLDWP
BUR_DAS5-001007	0.0000	0.0000	0.9999	0.0000	0.0000	0.0000	0.0000	0.0000	OLDWP
BUR_DAS5-001521	0.0000	0.0000	0.9999	0.0000	0.0000	0.0000	0.0000	0.0000	OLDWP
IND_DAS5-001550	0.0000	0.0000	0.9999	0.0000	0.0000	0.0000	0.0000	0.0000	OLDWP
OMA_DAS5-001657	0.0000	0.0000	0.9999	0.0000	0.0000	0.0000	0.0000	0.0000	OLDWP
EGY_DAS5-002464	0.0000	0.0000	0.9999	0.0000	0.0000	0.0000	0.0000	0.0000	OLDWP
FSV_DAS5-002959	0.0000	0.0000	0.9999	0.0000	0.0000	0.0000	0.0000	0.0000	OLDWP
AFG_DAS5-003279	0.0000	0.0000	0.9999	0.0000	0.0000	0.0000	0.0000	0.0000	OLDWP
CHN_DAS5-003455	0.0000	0.0000	0.9999	0.0000	0.0000	0.0000	0.0000	0.0000	OLDWP
IRN_DAS5-003690	0.0000	0.0000	0.9999	0.0000	0.0000	0.0000	0.0000	0.0000	OLDWP
ISR_PI-94567	0.0000	0.0000	0.9999	0.0000	0.0000	0.0000	0.0000	0.0000	OLDWP
UZB_PI-9791	0.0000	0.0000	0.9999	0.0000	0.0000	0.0000	0.0000	0.0000	OLDWP
CHN_WATKINS-007210	0.0000	0.0000	0.9999	0.0000	0.0000	0.0000	0.0000	0.0000	OLDWP
MEX_WATKINS-007251	0.0000	0.0000	0.9999	0.0000	0.0000	0.0000	0.0000	0.0000	OLDWP
ROM_WATKINS-007510	0.0000	0.0000	0.9999	0.0000	0.0000	0.0000	0.0000	0.0000	OLDWP
PAK_DAS5-001341	0.0000	0.0000	0.9999	0.0000	0.0000	0.0000	0.0000	0.0000	OLDWP
ANG_DAS5-001803	0.0000	0.0000	0.9999	0.0000	0.0000	0.0000	0.0000	0.0000	OLDWP
FSV_DAS5-003245	0.0000	0.0000	0.9999	0.0000	0.0000	0.0000	0.0000	0.0000	OLDWP
IRQ_WATKINS-007702	0.0000	0.0000	0.9999	0.0000	0.0000	0.0000	0.0000	0.0000	OLDWP
PAR_DAS5-001524	0.0000	0.0000	0.9999	0.0000	0.0000	0.0000	0.0000	0.0000	OLDWP
UZB_DAS5-001984	0.0000	0.0000	0.9999	0.0000	0.0000	0.0000	0.0000	0.0000	OLDWP
TAJ_DAS5-001916	0.0000	0.0000	0.9999	0.0000	0.0000	0.0000	0.0000	0.0000	OLDWP
UZB_DAS5-001577	0.0000	0.0000	0.9984	0.0000	0.0000	0.0000	0.0000	0.0015	OLDWP
CHN_WATKINS-007220	0.0017	0.0000	0.9982	0.0000	0.0000	0.0000	0.0000	0.0000	OLDWP
CHN_ChineseSpring	0.0026	0.0000	0.9974	0.0000	0.0000	0.0000	0.0000	0.0000	OLDWP
NEP_DAS5-002811	0.0000	0.0000	0.9912	0.0000	0.0088	0.0000	0.0000	0.0000	OLDWP
IRN_DAS5-003654	0.0000	0.0000	0.9896	0.0000	0.0000	0.0104	0.0000	0.0000	OLDWP
BHU_WSC-4-6	0.0000	0.0000	0.9877	0.0122	0.0000	0.0000	0.0000	0.0000	OLDWP
AFG_DAS5-004674	0.0144	0.0000	0.9855	0.0000	0.0000	0.0000	0.0000	0.0000	OLDWP
AFG_WATKINS-007049	0.0000	0.0122	0.9841	0.0000	0.0034	0.0003	0.0000	0.0000	OLDWP

Accessions	Q1	Q2	Q3	Q4	Q5	Q6	Q7	Q8	Subpop
AFG_WSC-4-2	0.0000	0.0000	0.9808	0.0000	0.0191	0.0000	0.0000	0.0000	OLDWP
MWI_PI-61693	0.0000	0.0153	0.9798	0.0049	0.0000	0.0000	0.0000	0.0000	OLDWP
TKM_WSC-7-1	0.0000	0.0000	0.9780	0.0000	0.0000	0.0000	0.0000	0.0220	OLDWP
COL_DAS5-001666	0.0000	0.0000	0.9748	0.0000	0.0000	0.0000	0.0000	0.0252	OLDWP
JAP_DAS5-003119	0.0261	0.0000	0.9738	0.0000	0.0000	0.0000	0.0000	0.0000	OLDWP
PHI_Cltr-4175	0.0263	0.0000	0.9736	0.0000	0.0000	0.0000	0.0000	0.0000	OLDWP
FSV_WATKINS-007547	0.0000	0.0000	0.9731	0.0000	0.0268	0.0000	0.0000	0.0000	OLDWP
AFG_DAS5-004583	0.0000	0.0000	0.9700	0.0000	0.0000	0.0000	0.0299	0.0000	OLDWP
UNK_DAS5-004209	0.0000	0.0000	0.9660	0.0000	0.0022	0.0000	0.0318	0.0000	OLDWP
AFG_WSC-4-9	0.0177	0.0000	0.9659	0.0000	0.0000	0.0000	0.0163	0.0000	OLDWP
IDN_DAS5-001690	0.0121	0.0000	0.9653	0.0183	0.0000	0.0043	0.0000	0.0000	OLDWP
BOL_DAS5-001094	0.0090	0.0000	0.9617	0.0292	0.0000	0.0000	0.0000	0.0000	OLDWP
FSV_WATKINS-007827	0.0019	0.0000	0.9560	0.0000	0.0000	0.0000	0.0000	0.0420	OLDWP
FSV_DAS5-002819	0.0000	0.0000	0.9543	0.0456	0.0000	0.0000	0.0000	0.0000	OLDWP
NEP_WSC-4-3	0.0084	0.0000	0.9515	0.0400	0.0000	0.0000	0.0000	0.0000	OLDWP
UZB_DAS5-004240	0.0000	0.0043	0.9505	0.0137	0.0000	0.0000	0.0222	0.0093	OLDWP
NEP_WSC-5-13	0.0152	0.0000	0.9328	0.0520	0.0000	0.0000	0.0000	0.0000	OLDWP
IRQ_WATKINS-007708	0.0000	0.0000	0.9119	0.0000	0.0196	0.0000	0.0153	0.0532	OLDWP
BUL_DAS5-001154	0.0000	0.0000	0.9058	0.0000	0.0389	0.0000	0.0070	0.0484	OLDWP
FSV_DAS5-002641	0.0942	0.0000	0.9048	0.0010	0.0000	0.0000	0.0000	0.0000	OLDWP
POR_DAS5-001847	0.0000	0.0049	0.9017	0.0000	0.0348	0.0000	0.0041	0.0544	OLDWP
SUD_WSC-4-5	0.0000	0.0121	0.8890	0.0037	0.0363	0.0000	0.0339	0.0249	OLDWP
AFG_DAS5-003288	0.0000	0.0000	0.8702	0.0000	0.0000	0.0000	0.0000	0.1297	OLDWP
CHN_WATKINS-007263	0.0653	0.0105	0.8626	0.0284	0.0000	0.0117	0.0214	0.0000	OLDWP
UZB_DAS5-001540	0.0031	0.0000	0.8556	0.0000	0.0300	0.0000	0.0238	0.0875	OLDWP
FSV_DAS5-003026	0.0087	0.0178	0.8552	0.0000	0.0043	0.0000	0.0000	0.1140	OLDWP
IRN_DAS5-003660	0.0088	0.0095	0.8478	0.0000	0.0681	0.0000	0.0348	0.0310	OLDWP
IRN_DAS5-003689	0.0038	0.0237	0.8390	0.0000	0.0000	0.0000	0.0480	0.0854	OLDWP
AUT_DAS5-001116	0.0036	0.0182	0.8365	0.0000	0.0000	0.0000	0.0000	0.1417	OLDWP
TAJ_DAS5-004406	0.0000	0.0000	0.8202	0.0000	0.0308	0.0000	0.0000	0.1489	OLDWP
JAP_DAS5-001696	0.1173	0.0000	0.8196	0.0000	0.0000	0.0000	0.0000	0.0631	OLDWP
TAN_DAS5-001205	0.0000	0.0288	0.8124	0.0000	0.0098	0.0000	0.0000	0.1490	OLDWP
CHA_DAS5-001251	0.0000	0.0296	0.8086	0.0000	0.0085	0.0000	0.0000	0.1532	OLDWP
SYR_DAS5-001152	0.0014	0.0000	0.8032	0.0000	0.0604	0.0000	0.0000	0.1349	OLDWP
FSV_DAS5-002350	0.0000	0.0000	0.8007	0.0000	0.0609	0.0000	0.0000	0.1384	OLDWP
USA_DAS5-001872	0.1010	0.0000	0.8006	0.0000	0.0000	0.0000	0.0000	0.0984	OLDWP
JOR_PI-283147	0.0000	0.0000	0.7880	0.1001	0.0327	0.0000	0.0000	0.0791	OLDWP
YEM_DAS5-004256	0.0000	0.0260	0.7826	0.0000	0.0126	0.0069	0.0000	0.1719	OLDWP
FSV_WATKINS-007789	0.0000	0.0000	0.7770	0.0000	0.1778	0.0000	0.0296	0.0156	OLDWP
YEM_DAS5-004253	0.0000	0.0217	0.7749	0.0000	0.0412	0.0000	0.0000	0.1622	OLDWP
IND_WATKINS-007581	0.0000	0.0485	0.7653	0.0000	0.0733	0.0000	0.0000	0.1129	OLDWP

Accessions	Q1	Q2	Q3	Q4	Q5	Q6	Q7	Q8	Subpop
AFG_PI-268466	0.0000	0.0476	0.7642	0.0000	0.0822	0.0000	0.0000	0.1060	OLDWP
NIG_DAS5-003881	0.0092	0.0296	0.7620	0.0000	0.0283	0.0000	0.0000	0.1709	OLDWP
IND_WATKINS-007606	0.0070	0.0049	0.7590	0.0427	0.0246	0.0000	0.0341	0.1278	OLDWP
IRQ_WSC-7-8	0.0000	0.0170	0.7449	0.0000	0.0818	0.0192	0.0000	0.1371	OLDWP
IRQ_WATKINS-007470	0.0000	0.0000	0.7361	0.0000	0.2201	0.0000	0.0010	0.0427	OLDWP
YEM_DAS5-001244	0.0433	0.0000	0.7305	0.1594	0.0211	0.0000	0.0456	0.0000	OLDWP
FSV_DAS5-002419	0.0000	0.0000	0.7291	0.0000	0.1908	0.0000	0.0000	0.0801	OLDWP
IND_WATKINS-007401	0.0000	0.0079	0.7171	0.0192	0.1081	0.0025	0.1451	0.0000	OLDWP
KOR_DAS5-001100	0.0038	0.0000	0.6896	0.0000	0.1569	0.0000	0.1497	0.0000	OLDWP
IRN_DAS5-001458	0.0000	0.0395	0.6797	0.0000	0.0866	0.0000	0.0392	0.1549	OLDWP
ETH_WSC-7-6	0.0000	0.0000	0.6715	0.0000	0.0805	0.0452	0.0526	0.1502	OLDWP
IND_WATKINS-007398	0.0000	0.0000	0.6705	0.1590	0.1322	0.0000	0.0000	0.0383	OLDWP
MLI_DAS5-001100	0.0122	0.0000	0.6595	0.2790	0.0492	0.0000	0.0000	0.0000	OLDWP
HON_DAS5-001079	0.0000	0.0068	0.6460	0.0000	0.0000	0.0000	0.0532	0.2940	OLDWP
IND_WSC-8-5	0.0095	0.0000	0.6441	0.2930	0.0533	0.0000	0.0000	0.0000	OLDWP
TUN_DAS5-004072	0.0000	0.0627	0.6423	0.0000	0.0000	0.0193	0.0000	0.2756	OLDWP
BRA_WATKINS-007705	0.0095	0.0646	0.6345	0.0022	0.1506	0.0144	0.0936	0.0306	OLDWP
EGY_PI-220431	0.0000	0.0026	0.6282	0.3407	0.0000	0.0000	0.0000	0.0285	OLDWP
IRN_DAS5-003641	0.0000	0.0000	0.6279	0.0000	0.1730	0.0000	0.0000	0.1990	OLDWP
USA_DAS5-004667	0.0284	0.0137	0.6147	0.2075	0.1357	0.0000	0.0000	0.0000	OLDWP
ETH_DAS5-003524	0.0000	0.0181	0.6028	0.0000	0.1095	0.0243	0.0442	0.2009	OLDWP
FSV_WATKINS-007137	0.0000	0.0000	0.5930	0.0000	0.3075	0.0000	0.0000	0.0995	OLDWP
FSV_WATKINS-007194	0.0000	0.0000	0.5761	0.0000	0.1826	0.0000	0.0000	0.2413	OLDWP
ARM_DAS5-003308	0.0000	0.0000	0.5733	0.0000	0.2635	0.0099	0.0000	0.1533	OLDWP
IND_WATKINS-007350	0.0026	0.0000	0.5579	0.1241	0.0000	0.0000	0.1257	0.1896	OLDWP
FSV_WATKINS-007548	0.0000	0.0000	0.5504	0.0000	0.2565	0.0092	0.0000	0.1839	OLDWP
KEN_DAS5-003754	0.0000	0.0197	0.5480	0.2528	0.1530	0.0000	0.0265	0.0000	OLDWP
AZE_DAS5-004465	0.0000	0.0000	0.5403	0.0000	0.2784	0.0077	0.0000	0.1735	OLDWP
AZE_WATKINS-007784	0.1003	0.0000	0.5393	0.0020	0.2550	0.0000	0.0170	0.0864	OLDWP
ZAF_DAS5-003999	0.0000	0.0390	0.5338	0.1697	0.0192	0.0956	0.0363	0.1063	OLDWP
GBR_DAS5-004158	0.0000	0.0000	0.5232	0.0992	0.2700	0.0000	0.1040	0.0036	OLDWP
USA_DAS5-001165	0.0609	0.0119	0.5217	0.2689	0.1356	0.0000	0.0000	0.0010	OLDWP
GEO_DAS5-003570	0.0049	0.0000	0.5191	0.0000	0.3233	0.0000	0.0000	0.1526	OLDWP
FSV_DAS5-003170	0.0000	0.0000	0.5094	0.0000	0.2706	0.0000	0.2199	0.0000	OLDWP
CAN_WATKINS-007472	0.0000	0.0000	0.5041	0.0000	0.2359	0.0000	0.0000	0.2599	OLDWP
IRN_DAS5-003661	0.0000	0.0000	0.5026	0.0000	0.2018	0.0000	0.0000	0.2955	OLDWP
ALG_WSC-5-12	0.0000	0.0623	0.4974	0.0000	0.0000	0.0212	0.0000	0.4190	OLDWP
AUS_DAS5-003327	0.0000	0.0372	0.4974	0.2358	0.1674	0.0038	0.0000	0.0583	OLDWP
FRA_WATKINS-007695	0.0000	0.0000	0.4942	0.0000	0.0726	0.0000	0.1668	0.2663	OLDWP
FSV_WATKINS-007191	0.0000	0.0000	0.4905	0.0000	0.3672	0.0000	0.0000	0.1423	OLDWP
ERI_DAS5-001368	0.0000	0.0152	0.4900	0.0000	0.2443	0.0098	0.0000	0.2407	OLDWP

Accessions	Q1	Q2	Q3	Q4	Q5	Q6	Q7	Q8	Subpop
IND_WATKINS-007383	0.0148	0.0000	0.4779	0.0732	0.1572	0.0000	0.2769	0.0000	OLDWP
AUS_WATKINS-007036	0.0000	0.0372	0.4748	0.0000	0.1032	0.0424	0.0697	0.2727	OLDWP
CHN_WATKINS-007218	0.0000	0.0000	0.4435	0.1315	0.2472	0.0000	0.1663	0.0115	OLDWP
FSV_DAS5-002970	0.0000	0.3597	0.4406	0.0000	0.1765	0.0000	0.0232	0.0000	OLDWP
SAU_DAS5-003980	0.0212	0.0000	0.4293	0.0000	0.1754	0.0175	0.0000	0.3565	OLDWP
CHN_WATKINS-007250	0.0829	0.0000	0.4283	0.0541	0.0503	0.0000	0.3844	0.0000	OLDWP
MEX_WATKINS-007233	0.0000	0.0112	0.4211	0.1092	0.0628	0.0000	0.3536	0.0421	OLDWP
CHN_WATKINS-007256	0.0712	0.0000	0.4201	0.0470	0.0622	0.0000	0.3994	0.0000	OLDWP
FSV_DAS5-002492	0.0000	0.0000	0.4091	0.0136	0.2710	0.0017	0.0174	0.2872	OLDWP
CHN_WATKINS-007276	0.0033	0.0000	0.4089	0.0000	0.3197	0.0000	0.1921	0.0761	OLDWP
FSV_DAS5-002352	0.0000	0.0000	0.4059	0.0000	0.3273	0.0000	0.0000	0.2667	OLDWP
ROM_WATKINS-007515	0.0043	0.0000	0.3987	0.0000	0.3504	0.0000	0.2465	0.0000	OLDWP
SWE_DAS5-004415	0.0521	0.0902	0.3934	0.0000	0.1663	0.0482	0.0832	0.1666	OLDWP
EGY_DAS5-002920	0.0000	0.0347	0.3922	0.0690	0.3604	0.0000	0.0488	0.0949	OLDWP
AUS_DAS5-003227	0.0000	0.0000	0.3760	0.2960	0.3279	0.0000	0.0000	0.0000	OLDWP
SUD_DAS5-001470	0.1277	0.1166	0.3494	0.2835	0.0000	0.1227	0.0000	0.0000	OLDWP
GEO_DAS5-003577	0.0561	0.0948	0.3489	0.0000	0.0789	0.0570	0.0358	0.3285	OLDWP
TKM_DAS5-001564	0.0000	0.0000	0.3488	0.0000	0.3236	0.0000	0.0000	0.3276	OLDWP
UNK_WATKINS-007801	0.0004	0.0173	0.3454	0.0595	0.0076	0.0000	0.3257	0.2440	OLDWP
CHN_WATKINS-007209	0.0566	0.0000	0.3332	0.0000	0.0000	0.0000	0.2918	0.3183	OLDWP
FSV_DAS5-003243	0.0222	0.0000	0.3316	0.0000	0.1278	0.0028	0.3723	0.1433	OLDWP
BEL_DAS5-003360	0.0363	0.0996	0.3241	0.1172	0.1419	0.0000	0.1544	0.1265	OLDWP
AFG_WATKINS-007770	0.0171	0.0055	0.3077	0.0000	0.2972	0.0033	0.1962	0.1731	OLDWP
SPA_WATKINS-007661	0.0000	0.0000	0.3038	0.0000	0.1206	0.0000	0.2785	0.2970	OLDWP
POL_DAS5-002871	0.0131	0.0000	0.3021	0.0354	0.1773	0.0000	0.2036	0.2684	OLDWP
IND_WATKINS-007576	0.0174	0.0126	0.3011	0.0620	0.2427	0.0137	0.2140	0.1365	OLDWP
ROM_DAS5-002944	0.0000	0.0000	0.2994	0.0000	0.2931	0.0000	0.1778	0.2297	OLDWP
ZAF_DAS5-004000	0.0000	0.0616	0.2834	0.2713	0.0508	0.0775	0.1314	0.1239	OLDWP
USA_DAS5-001195	0.0002	0.0000	0.0000	0.9876	0.0000	0.0000	0.0121	0.0000	WLDMix
AUS_DAS5-002909	0.0697	0.0000	0.0000	0.9303	0.0000	0.0000	0.0000	0.0000	WLDMix
ALG_WSC-5-14	0.1374	0.0000	0.0000	0.8626	0.0000	0.0000	0.0000	0.0000	WLDMix
USA_DAS5-003231	0.0754	0.0010	0.0000	0.8557	0.0000	0.0208	0.0000	0.0471	WLDMix
USA_WSC-2-2	0.1559	0.0000	0.0000	0.8440	0.0000	0.0000	0.0000	0.0000	WLDMix
AUS_DAS5-CORRELL	0.0000	0.0000	0.0000	0.8420	0.0000	0.0958	0.0000	0.0622	WLDMix
USA_DAS5-003232	0.1596	0.0000	0.0000	0.8404	0.0000	0.0000	0.0000	0.0000	WLDMix
MEX_WSC-1-3	0.1433	0.0000	0.0000	0.8336	0.0105	0.0000	0.0126	0.0000	WLDMix
USA_DAS5-001463	0.0554	0.0546	0.0000	0.8223	0.0000	0.0000	0.0676	0.0000	WLDMix
AUS_DAS5-KENNEDY	0.1884	0.0000	0.0000	0.8115	0.0000	0.0000	0.0000	0.0000	WLDMix
USA_DAS5-003234	0.0734	0.0000	0.0000	0.7923	0.0000	0.0000	0.1342	0.0000	WLDMix
ARG_DAS5-002017	0.0592	0.0000	0.0000	0.7676	0.1435	0.0000	0.0297	0.0000	WLDMix
AUS_DAS5-AXE	0.0605	0.0000	0.0199	0.7650	0.0139	0.0000	0.1406	0.0000	WLDMix

Accessions	Q1	Q2	Q3	Q4	Q5	Q6	Q7	Q8	Subpop
MEX_WSC-6-9	0.0985	0.0000	0.0000	0.7569	0.1255	0.0000	0.0192	0.0000	WLDMix
CAN_DAS5-002218	0.0209	0.1009	0.0000	0.7541	0.0000	0.0028	0.0952	0.0261	WLDMix
ZIM_DAS5-002482	0.0556	0.0439	0.0999	0.7499	0.0184	0.0000	0.0000	0.0323	WLDMix
AUS_DAS5-WYALKATCHEM	0.2107	0.0000	0.0000	0.7492	0.0000	0.0000	0.0011	0.0389	WLDMix
USA_WSC-4-1	0.1478	0.0000	0.0995	0.7458	0.0000	0.0000	0.0069	0.0000	WLDMix
MEX_DAS5-003809	0.1708	0.0000	0.0000	0.7457	0.0834	0.0000	0.0000	0.0000	WLDMix
MEX_WSC-2-6	0.2583	0.0000	0.0000	0.7416	0.0000	0.0000	0.0000	0.0000	WLDMix
MEX_DAS5-003811	0.1569	0.0000	0.0000	0.7390	0.1040	0.0000	0.0000	0.0000	WLDMix
SWI_WSC-4-4	0.0427	0.1105	0.0402	0.7385	0.0681	0.0000	0.0000	0.0000	WLDMix
AUS_DAS5-HARTOG	0.2658	0.0000	0.0000	0.7341	0.0000	0.0000	0.0000	0.0000	WLDMix
FSV_DAS5-002443	0.0000	0.0536	0.0076	0.7334	0.1977	0.0000	0.0000	0.0076	WLDMix
USA_DAS5-LINCOLN	0.1819	0.0000	0.0547	0.7307	0.0000	0.0000	0.0000	0.0327	WLDMix
MEX_DAS5-003817	0.2179	0.0000	0.0000	0.7305	0.0000	0.0000	0.0515	0.0000	WLDMix
AUS_DAS5-BRAHAM	0.0643	0.0000	0.0000	0.7301	0.0934	0.0183	0.0699	0.0240	WLDMix
AUS_DAS5-ESPADA	0.0567	0.0000	0.0445	0.7298	0.1549	0.0136	0.0005	0.0000	WLDMix
MEX_DAS5-002705	0.1251	0.0000	0.0000	0.7297	0.0000	0.0000	0.0000	0.1451	WLDMix
AUS_DAS5-FRAME	0.0312	0.0000	0.0000	0.7282	0.0581	0.0184	0.0312	0.1328	WLDMix
AUS_DAS5-000110	0.0000	0.0142	0.1611	0.7194	0.1054	0.0000	0.0000	0.0000	WLDMix
SYR_WSC-6-3	0.2167	0.0000	0.0000	0.7184	0.0000	0.0000	0.0649	0.0000	WLDMix
ARG_DAS5-002590	0.0942	0.0000	0.0000	0.7165	0.0096	0.0000	0.1797	0.0000	WLDMix
AUS_DAS5-002644	0.0866	0.0000	0.0000	0.7128	0.0181	0.0000	0.1825	0.0000	WLDMix
MEX_DAS5-003815	0.1019	0.0000	0.0439	0.7127	0.0706	0.0000	0.0709	0.0000	WLDMix
ECU_DAS5-001572	0.0000	0.0174	0.1729	0.7119	0.0977	0.0000	0.0000	0.0000	WLDMix
ARG_DAS5-002577	0.0000	0.0000	0.0000	0.7103	0.0370	0.0000	0.2527	0.0000	WLDMix
JAP_DAS5-001830	0.0000	0.0135	0.1641	0.7041	0.1184	0.0000	0.0000	0.0000	WLDMix
AUS_DAS5-CARNAMAH	0.2092	0.0000	0.0386	0.7010	0.0420	0.0000	0.0091	0.0000	WLDMix
AUS_DAS5-000350	0.0844	0.0000	0.0000	0.6999	0.0000	0.1835	0.0322	0.0000	WLDMix
USA_WSC-3-7	0.0637	0.0000	0.0826	0.6957	0.0000	0.0000	0.1580	0.0000	WLDMix
USA_Choteau	0.0437	0.0140	0.0000	0.6837	0.1041	0.0000	0.0591	0.0954	WLDMix
USA_WSC-3-14	0.1357	0.0000	0.0165	0.6795	0.0000	0.0000	0.0795	0.0887	WLDMix
USA_DAS5-001496	0.0507	0.0000	0.0174	0.6783	0.0000	0.0000	0.1171	0.1364	WLDMix
AUS_WSC-1-1	0.0339	0.0000	0.0000	0.6770	0.1685	0.0428	0.0777	0.0000	WLDMix
USA_DAS5-001767	0.0455	0.0525	0.0000	0.6765	0.1017	0.0068	0.0411	0.0759	WLDMix
PAK_WSC-6-8	0.1059	0.0000	0.0506	0.6730	0.0000	0.0000	0.1705	0.0000	WLDMix
ANG_DAS5-004675	0.1788	0.0000	0.0000	0.6693	0.0000	0.0000	0.1518	0.0000	WLDMix
IND_WSC-6-1	0.0682	0.0000	0.1862	0.6689	0.0766	0.0000	0.0000	0.0000	WLDMix
AUS_DAS5-003131	0.0000	0.0000	0.0161	0.6687	0.0742	0.0118	0.0351	0.1941	WLDMix
MEX_DAS5-003794	0.0011	0.0694	0.0000	0.6655	0.0000	0.0846	0.0408	0.1387	WLDMix
USA_WSC-3-12	0.1450	0.0000	0.0032	0.6641	0.0000	0.0000	0.1877	0.0000	WLDMix
POR_DAS5-001382	0.0000	0.0465	0.0000	0.6610	0.0000	0.0564	0.0000	0.2360	WLDMix
USA_WSC-3-13	0.0418	0.0000	0.0181	0.6602	0.0000	0.0000	0.2799	0.0000	WLDMix

Accessions	Q1	Q2	Q3	Q4	Q5	Q6	Q7	Q8	Subpop
IND_WSC-1-9	0.1088	0.0000	0.0606	0.6584	0.0000	0.0000	0.1721	0.0000	WLDMix
SAU_DAS5-003977	0.2503	0.0000	0.0000	0.6581	0.0019	0.0000	0.0896	0.0000	WLDMix
ISR_DAS5-003698	0.1086	0.0908	0.0570	0.6520	0.0000	0.0875	0.0042	0.0000	WLDMix
ZAF_DAS5-003120	0.1588	0.0371	0.0613	0.6359	0.0670	0.0000	0.0374	0.0025	WLDMix
ARG_WSC-2-5	0.1200	0.0000	0.0000	0.6338	0.2461	0.0000	0.0000	0.0000	WLDMix
ZIM_DAS5-004536	0.1168	0.0000	0.0782	0.6203	0.0860	0.0000	0.0631	0.0356	WLDMix
BOL_DAS5-002237	0.0907	0.0000	0.0658	0.6166	0.0000	0.0000	0.2268	0.0000	WLDMix
ISR_DAS5-001225	0.0733	0.0122	0.0556	0.6162	0.1567	0.0000	0.0860	0.0000	WLDMix
CHL_DAS5-003442	0.1338	0.0119	0.0476	0.6138	0.0000	0.0000	0.1928	0.0000	WLDMix
AUS_DAS5-ELLISON	0.1674	0.0108	0.1535	0.6124	0.0559	0.0000	0.0000	0.0000	WLDMix
MEX_WSC-2-4	0.0524	0.0105	0.0000	0.5994	0.2037	0.1290	0.0049	0.0000	WLDMix
COL_DAS5-003123	0.1367	0.0000	0.0000	0.5953	0.0000	0.0000	0.2679	0.0000	WLDMix
COL_DAS5-003468	0.1818	0.0000	0.0024	0.5930	0.0732	0.0000	0.1496	0.0000	WLDMix
ARG_DAS5-002555	0.1278	0.0000	0.0000	0.5912	0.0000	0.0000	0.1328	0.1481	WLDMix
AUS_DAS5-SHAW-VB	0.0629	0.0742	0.0000	0.5868	0.2485	0.0000	0.0276	0.0000	WLDMix
ARG_DAS5-002173	0.0542	0.0090	0.0000	0.5839	0.1429	0.0000	0.2099	0.0000	WLDMix
ERI_DAS5-001365	0.0424	0.0124	0.0800	0.5819	0.2832	0.0000	0.0000	0.0000	WLDMix
ZAF_DAS5-004005	0.0983	0.0913	0.0418	0.5819	0.1410	0.0213	0.0000	0.0245	WLDMix
MEX_DAS5-001740	0.0063	0.0000	0.0762	0.5792	0.2460	0.0000	0.0159	0.0763	WLDMix
BOL_DAS5-003387	0.1028	0.0000	0.0886	0.5772	0.0000	0.0000	0.2313	0.0000	WLDMix
PER_DAS5-003907	0.0000	0.0000	0.0054	0.5747	0.0438	0.0000	0.2944	0.0817	WLDMix
COL_DAS5-003469	0.1647	0.0000	0.0233	0.5707	0.1230	0.0000	0.1184	0.0000	WLDMix
AUS_DAS5-000439	0.0000	0.0000	0.0042	0.5696	0.0000	0.4029	0.0000	0.0233	WLDMix
MOR_DAS5-003838	0.0970	0.0000	0.0657	0.5596	0.2777	0.0000	0.0000	0.0000	WLDMix
AUS_DAS5-000305	0.0000	0.0000	0.0000	0.5590	0.0000	0.4390	0.0020	0.0000	WLDMix
AUS_DAS5-QALBIS	0.1004	0.0000	0.0000	0.5563	0.0944	0.0000	0.1019	0.1469	WLDMix
KEN_DAS5-003744	0.0000	0.0233	0.0000	0.5546	0.1115	0.0000	0.0374	0.2732	WLDMix
AUS_DAS5-003319	0.0000	0.0000	0.0573	0.5520	0.2947	0.0000	0.0000	0.0960	WLDMix
COL_DAS5-001955	0.0590	0.0000	0.0000	0.5465	0.2460	0.0000	0.1484	0.0000	WLDMix
EGY_DAS5-002411	0.0175	0.0187	0.4120	0.5446	0.0020	0.0012	0.0000	0.0040	WLDMix
EGY_DAS5-001829	0.0000	0.0076	0.3792	0.5439	0.0582	0.0111	0.0000	0.0000	WLDMix
ISR_DAS5-001016	0.0138	0.1100	0.1787	0.5400	0.0620	0.0795	0.0160	0.0000	WLDMix
EGY_DAS5-001149	0.0000	0.0040	0.4032	0.5385	0.0398	0.0145	0.0000	0.0000	WLDMix
AUS_DAS5-003320	0.0000	0.0000	0.0688	0.5382	0.3139	0.0000	0.0000	0.0790	WLDMix
ARG_DAS5-002166	0.1221	0.0133	0.0000	0.5365	0.1452	0.0000	0.1552	0.0276	WLDMix
ARG_DAS5-002178	0.1213	0.0054	0.0000	0.5341	0.1634	0.0000	0.0000	0.1757	WLDMix
ZAM_DAS5-001488	0.0893	0.0000	0.0870	0.5328	0.1022	0.0000	0.0000	0.1887	WLDMix
AUS_DAS5-000265	0.0000	0.0000	0.0259	0.5294	0.0000	0.2878	0.1548	0.0021	WLDMix
ISR_DAS5-003707	0.0728	0.0000	0.2812	0.5264	0.1099	0.0000	0.0098	0.0000	WLDMix
ARG_DAS5-002566	0.1332	0.0000	0.0000	0.5220	0.0577	0.0000	0.1652	0.1219	WLDMix
FSV_DAS5-003024	0.2144	0.0116	0.0031	0.5217	0.1211	0.0027	0.0720	0.0534	WLDMix

Accessions	Q1	Q2	Q3	Q4	Q5	Q6	Q7	Q8	Subpop
MEX_DAS5-002004	0.1046	0.0433	0.1866	0.5216	0.0000	0.0832	0.0000	0.0608	WLDMix
AUS_DAS5-000297	0.0943	0.0007	0.0000	0.5204	0.1086	0.1400	0.1288	0.0071	WLDMix
AUS_DAS5-H45	0.4094	0.0000	0.0000	0.5190	0.0257	0.0000	0.0458	0.0000	WLDMix
AUS_DAS5-000317	0.0910	0.0000	0.0113	0.5133	0.0456	0.2827	0.0561	0.0000	WLDMix
PER_DAS5-003905	0.0000	0.0000	0.2752	0.5126	0.1434	0.0000	0.0687	0.0000	WLDMix
MEX_DAS5-BOBWHITE	0.0667	0.0000	0.0000	0.5085	0.2579	0.0000	0.1670	0.0000	WLDMix
YEM_DAS5-004257	0.0927	0.0000	0.0704	0.5034	0.3086	0.0000	0.0249	0.0000	WLDMix
TUN_DAS5-004078	0.0196	0.0000	0.0773	0.5028	0.4003	0.0000	0.0000	0.0000	WLDMix
BUR_DAS5-001002	0.0000	0.1047	0.0914	0.4997	0.0000	0.0540	0.0671	0.1831	WLDMix
AUS_DAS5-000336	0.1345	0.0000	0.0000	0.4997	0.0034	0.3215	0.0409	0.0000	WLDMix
CHN_DAS5-001309	0.0880	0.0000	0.0819	0.4987	0.0000	0.0000	0.3219	0.0095	WLDMix
AUS_DAS5-000291	0.0000	0.0058	0.0000	0.4979	0.1077	0.2363	0.1190	0.0333	WLDMix
ARG_DAS5-002014	0.0244	0.0058	0.0000	0.4953	0.1005	0.0000	0.1083	0.2657	WLDMix
JAP_WSC-4-8	0.0473	0.1286	0.0058	0.4943	0.1303	0.0000	0.0026	0.1911	WLDMix
AUS_DAS5-000250	0.0454	0.0157	0.0000	0.4935	0.0105	0.4274	0.0048	0.0027	WLDMix
AUS_DAS5-000032	0.0000	0.0000	0.0000	0.4907	0.0000	0.3544	0.0191	0.1357	WLDMix
BUR_DAS5-003428	0.0000	0.0610	0.1589	0.4885	0.1288	0.0320	0.0479	0.0829	WLDMix
MEX_DAS5-001752	0.0891	0.0000	0.1021	0.4848	0.2193	0.0000	0.0151	0.0896	WLDMix
JAP_DAS5-002487	0.0437	0.1364	0.0000	0.4846	0.1447	0.0000	0.0000	0.1906	WLDMix
COL_DAS5-003471	0.0431	0.0165	0.0230	0.4841	0.2460	0.0000	0.1519	0.0354	WLDMix
MEX_DAS5-002368	0.1004	0.0061	0.0408	0.4809	0.0730	0.0000	0.2988	0.0000	WLDMix
USA_Vida	0.1191	0.0259	0.0190	0.4792	0.0588	0.0000	0.1149	0.1830	WLDMix
KEN_DAS5-001548	0.0221	0.0139	0.0513	0.4783	0.1961	0.0000	0.1927	0.0455	WLDMix
AUS_DAS5-000318	0.1284	0.0016	0.0000	0.4773	0.0000	0.2656	0.1271	0.0000	WLDMix
AUS_DAS5-000263	0.0000	0.0026	0.0182	0.4750	0.0914	0.3516	0.0612	0.0000	WLDMix
KEN_DAS5-003753	0.0000	0.1254	0.0485	0.4738	0.1309	0.1145	0.0655	0.0413	WLDMix
SAU_Cltr-15144	0.0000	0.0071	0.3802	0.4703	0.1190	0.0233	0.0000	0.0000	WLDMix
AUS_DAS5-EGA-WYLIE	0.3917	0.0029	0.0000	0.4686	0.0557	0.0000	0.0810	0.0000	WLDMix
PER_WSC-5-7	0.0820	0.0169	0.0156	0.4667	0.1597	0.0000	0.2105	0.0485	WLDMix
LIB_PI-54431	0.0000	0.0000	0.1572	0.4666	0.3022	0.0000	0.0000	0.0740	WLDMix
ARG_DAS5-002180	0.0687	0.0207	0.0000	0.4651	0.1284	0.0005	0.1212	0.1954	WLDMix
ARG_DAS5-002255	0.1194	0.0323	0.0000	0.4591	0.1217	0.0000	0.1242	0.1432	WLDMix
AUS_DAS5-003337	0.0000	0.0000	0.0397	0.4584	0.2623	0.0000	0.0369	0.2028	WLDMix
EGY_DAS5-003502	0.0000	0.0000	0.4000	0.4574	0.1112	0.0313	0.0000	0.0000	WLDMix
AUS_DAS5-000298	0.0064	0.0000	0.0000	0.4567	0.0497	0.3589	0.1282	0.0000	WLDMix
Bakhatwa	0.3668	0.0000	0.0000	0.4555	0.1303	0.0000	0.0474	0.0000	WLDMix
USA_DAS5-ALPOWA	0.0587	0.0000	0.0133	0.4504	0.2913	0.0000	0.0413	0.1448	WLDMix
ZAM_DAS5-001490	0.0000	0.0000	0.1446	0.4429	0.1010	0.0000	0.0715	0.2399	WLDMix
MEX_DAS5-003805	0.0710	0.0000	0.0412	0.4394	0.0000	0.2889	0.1595	0.0000	WLDMix
CHL_DAS5-003447	0.1633	0.0000	0.0430	0.4388	0.1462	0.0000	0.1115	0.0973	WLDMix
ECU_DAS5-003499	0.3704	0.0000	0.0000	0.4364	0.1932	0.0000	0.0000	0.0000	WLDMix

Accessions	Q1	Q2	Q3	Q4	Q5	Q6	Q7	Q8	Subpop
ECU_DAS5-001191	0.1457	0.0035	0.0644	0.4329	0.2123	0.0000	0.1411	0.0000	WLDMix
AUS_DAS5-000339	0.1671	0.0000	0.0000	0.4312	0.0000	0.3556	0.0461	0.0000	WLDMix
YEM_DAS5-004245	0.1249	0.0000	0.1990	0.4309	0.0000	0.0000	0.1499	0.0953	WLDMix
PER_DAS5-003909	0.0000	0.0000	0.2757	0.4207	0.1366	0.0000	0.0422	0.1249	WLDMix
CZE_DAS5-001559	0.1728	0.0000	0.0000	0.4185	0.1011	0.0000	0.2027	0.1050	WLDMix
BRA_DAS5-003405	0.1455	0.0332	0.0997	0.4177	0.0430	0.0000	0.1238	0.1371	WLDMix
FSV_WATKINS-007192	0.0000	0.0000	0.0904	0.4067	0.1961	0.0000	0.0000	0.3067	WLDMix
ZAF_DAS5-004001	0.0816	0.0245	0.1091	0.4066	0.0000	0.0000	0.3547	0.0235	WLDMix
VEN_DAS5-001433	0.0922	0.0341	0.1528	0.4064	0.0752	0.0000	0.0000	0.2394	WLDMix
PER_DAS5-003908	0.0052	0.0000	0.2566	0.4054	0.0000	0.0000	0.2454	0.0874	WLDMix
CAN_DAS5-003430	0.0000	0.0578	0.0162	0.4044	0.4200	0.0000	0.1016	0.0000	WLDMix
ARG_DAS5-002192	0.0845	0.0000	0.0000	0.4029	0.3352	0.0000	0.1615	0.0159	WLDMix
ARG_DAS5-003299	0.0397	0.0043	0.1574	0.3974	0.2286	0.0257	0.1095	0.0374	WLDMix
AUS_DAS5-000295	0.0820	0.0000	0.0065	0.3872	0.0621	0.2716	0.1906	0.0000	WLDMix
ARG_DAS5-002549	0.0000	0.0113	0.0753	0.3853	0.3528	0.0000	0.0552	0.1201	WLDMix
USA_DAS5-002652	0.0027	0.0057	0.2078	0.3835	0.3760	0.0046	0.0195	0.0000	WLDMix
AUS_DAS5-000311	0.1721	0.0000	0.0130	0.3788	0.0048	0.3238	0.1076	0.0000	WLDMix
USA_DAS5-002775	0.0175	0.0448	0.0000	0.3770	0.1244	0.0000	0.1433	0.2930	WLDMix
AUS_DAS5-003338	0.0000	0.0971	0.0598	0.3770	0.2121	0.0000	0.0621	0.1919	WLDMix
ARG_DAS5-002584	0.1698	0.0615	0.0000	0.3641	0.0425	0.0980	0.2421	0.0219	WLDMix
ARM_DAS5-003301	0.0485	0.0000	0.2329	0.3544	0.2230	0.0172	0.1092	0.0147	WLDMix
BRA_DAS5-003402	0.0904	0.0000	0.0700	0.3456	0.0512	0.0000	0.2838	0.1589	WLDMix
FSV_DAS5-002467	0.0000	0.1504	0.1772	0.3436	0.2878	0.0231	0.0177	0.0000	WLDMix
ARG_DAS5-002174	0.0148	0.0216	0.0000	0.3395	0.2685	0.0000	0.1301	0.2254	WLDMix
PER_DAS5-003911	0.0000	0.0000	0.2829	0.3350	0.2218	0.0000	0.0769	0.0834	WLDMix
ZIM_DAS5-001471	0.0000	0.1231	0.0000	0.3233	0.0962	0.0829	0.0970	0.2774	WLDMix
ECU_DAS5-003497	0.2115	0.0000	0.1685	0.3223	0.0000	0.0000	0.2976	0.0000	WLDMix
ANG_DAS5-001265	0.0000	0.0622	0.1339	0.3206	0.1900	0.0827	0.0000	0.2106	WLDMix
AUS_DAS5-003316	0.0000	0.0311	0.1171	0.3029	0.1816	0.0005	0.0673	0.2993	WLDMix
ARG_DAS5-004653	0.0069	0.0031	0.1336	0.2947	0.1625	0.0243	0.1464	0.2284	WLDMix
ETH_DAS5-001163	0.0666	0.0000	0.2501	0.2941	0.2538	0.0084	0.0463	0.0807	WLDMix
NEW_DAS5-001196	0.0388	0.0936	0.0849	0.2870	0.1889	0.0089	0.2095	0.0884	WLDMix
LEB_DAS5-001551	0.0250	0.0065	0.1946	0.2845	0.1479	0.0016	0.1675	0.1723	WLDMix
ZAF_DAS5-001866	0.0297	0.1199	0.1194	0.2322	0.1956	0.1834	0.0783	0.0416	WLDMix
POL_DAS5-002295	0.0503	0.2987	0.0985	0.1914	0.1773	0.0000	0.1837	0.0000	WLDMix

Accession: the first three letters indicate the United Nations country naming code from where the accession originated followed by accession name.

Table S2. The list of accessions (n=347) in the landrace subset of the exome data set and ancestral coefficient of each accession based on ancestral population defined at *K*=7.

Accession	Q1	Q2	Q3	Q4	Q5	Q6	Q7	Subpop
ROM_DAS5-002943	0.0000	0.0000	0.0000	0.0000	0.0000	0.9999	0.0000	EEU
FSV_DAS5-003162	0.0000	0.0000	0.0000	0.0000	0.0000	0.9999	0.0000	EEU
FSV_DAS5-003175	0.0000	0.0000	0.0000	0.0000	0.0000	0.9999	0.0000	EEU
TUR_WATKINS-007033	0.0000	0.0000	0.0000	0.0000	0.0000	0.9999	0.0000	EEU
UNK_WATKINS-007096	0.0000	0.0000	0.0000	0.0000	0.0000	0.9999	0.0000	EEU
UNK_WATKINS-007099	0.0000	0.0000	0.0000	0.0000	0.0000	0.9999	0.0000	EEU
AZE_WATKINS-007170	0.0000	0.0000	0.0000	0.0000	0.0000	0.9999	0.0000	EEU
BRA_WATKINS-007179	0.0000	0.0000	0.0000	0.0000	0.0000	0.9999	0.0000	EEU
IRN_WATKINS-007697	0.0000	0.0000	0.0000	0.0000	0.0000	0.9999	0.0000	EEU
FSV_DAS5-003174	0.0000	0.0000	0.0000	0.0000	0.0000	0.9999	0.0000	EEU
BUL_WATKINS-007565	0.0097	0.0000	0.0000	0.0000	0.0000	0.9902	0.0000	EEU
POL_DAS5-002877	0.0000	0.0000	0.0000	0.0122	0.0000	0.9878	0.0000	EEU
ROM_DAS5-002946	0.0153	0.0000	0.0000	0.0000	0.0000	0.9653	0.0193	EEU
POL_DAS5-002878	0.0013	0.0000	0.0000	0.0564	0.0000	0.9422	0.0000	EEU
YUG_WATKINS-007292	0.0464	0.0000	0.0000	0.0126	0.0000	0.9410	0.0000	EEU
FSV_DAS5-003163	0.0497	0.0000	0.0000	0.0000	0.0000	0.9376	0.0127	EEU
BUL_WATKINS-007763	0.0000	0.0000	0.0853	0.0000	0.0000	0.9146	0.0000	EEU
BUL_WATKINS-007563	0.0220	0.0000	0.0000	0.0656	0.0000	0.9123	0.0000	EEU
POL_DAS5-002862	0.0157	0.0000	0.0000	0.0931	0.0000	0.8912	0.0000	EEU
EGY_WATKINS-007325	0.0000	0.0000	0.0329	0.0820	0.0000	0.8851	0.0000	EEU
YUG_WATKINS-007290	0.0000	0.0000	0.0000	0.1237	0.0000	0.8763	0.0000	EEU
EUR_DAS5-004508	0.0503	0.0151	0.0069	0.1074	0.0000	0.8202	0.0000	EEU
FSV_WATKINS-007536	0.0367	0.0000	0.0295	0.0460	0.0657	0.8093	0.0129	EEU
MON_DAS5-004267	0.0089	0.0936	0.0281	0.0000	0.0060	0.8010	0.0624	EEU
ROM_DAS5-002945	0.0298	0.0102	0.0367	0.0649	0.0191	0.7986	0.0406	EEU
GRE_WATKINS-007738	0.0000	0.1132	0.0430	0.0678	0.0000	0.7536	0.0224	EEU
YUG_WATKINS-007174	0.0000	0.1959	0.0000	0.0544	0.0000	0.7497	0.0000	EEU
POL_DAS5-002876	0.0356	0.0000	0.0000	0.2174	0.0000	0.7470	0.0000	EEU
POL_DAS5-002863	0.0000	0.0000	0.0000	0.2573	0.0000	0.7427	0.0000	EEU
AUT_DAS5-003343	0.0543	0.1130	0.1105	0.0000	0.0000	0.7222	0.0000	EEU
FSV_DAS5-002494	0.0623	0.0818	0.1390	0.0000	0.0334	0.6834	0.0000	EEU
YUG_WATKINS-007061	0.0000	0.2080	0.0101	0.1022	0.0000	0.6797	0.0000	EEU
BUL_DAS5-003205	0.0871	0.1762	0.0512	0.0120	0.0000	0.6736	0.0000	EEU
GRE_WATKINS-007523	0.0000	0.2661	0.0000	0.0000	0.0000	0.6718	0.0621	EEU
GRE_WATKINS-007524	0.0000	0.1385	0.0435	0.1192	0.0043	0.6659	0.0286	EEU
BUL_WATKINS-007769	0.0000	0.0000	0.0000	0.3505	0.0000	0.6495	0.0000	EEU
AUT_DAS5-003342	0.0497	0.1216	0.1745	0.0000	0.0150	0.6392	0.0000	EEU
DEN_DAS5-003490	0.0465	0.0682	0.0635	0.1313	0.0569	0.6336	0.0000	EEU
FSV_DAS5-003244	0.0000	0.0348	0.3619	0.0000	0.0000	0.6033	0.0000	EEU

Accession	Q1	Q2	Q3	Q4	Q5	Q6	Q7	Subpop
BEL_DAS5-004342	0.0000	0.0000	0.0000	0.3858	0.0165	0.5793	0.0184	EEU
POL_DAS5-002864	0.0000	0.0000	0.0000	0.4252	0.0000	0.5747	0.0000	EEU
MOR_WATKINS-007435	0.0213	0.1674	0.0000	0.0390	0.1976	0.5537	0.0210	EEU
CHN_WATKINS-007244	0.0746	0.0000	0.0000	0.3858	0.0000	0.5396	0.0000	EEU
YUG_WATKINS-007803	0.2218	0.0000	0.0000	0.1566	0.0079	0.5190	0.0947	EEU
JAP_DAS5-003729	0.0324	0.0000	0.0000	0.4681	0.0000	0.4995	0.0000	EEU
FSV_DAS5-003172	0.1155	0.1532	0.2481	0.0000	0.0000	0.4832	0.0000	EEU
DEN_DAS5-003494	0.0475	0.0068	0.0047	0.4474	0.0106	0.4829	0.0000	EEU
AFG_WATKINS-007108	0.0000	0.0000	0.3484	0.1066	0.0000	0.4805	0.0645	EEU
SPA_WATKINS-007190	0.0523	0.3599	0.0000	0.1061	0.0104	0.4713	0.0000	EEU
ROM_WATKINS-007515	0.0140	0.0000	0.1364	0.2139	0.0000	0.4062	0.2295	EEU
CHN_WATKINS-007276	0.0000	0.0284	0.1885	0.1652	0.0000	0.3940	0.2238	EEU
CHN_WATKINS-007218	0.0312	0.0000	0.1232	0.1749	0.0000	0.3645	0.3062	EEU
AFG_WATKINS-007770	0.0047	0.0549	0.2927	0.2351	0.0069	0.3369	0.0688	EEU
IRN_WATKINS-007715	0.0118	0.1638	0.1711	0.2057	0.0372	0.3365	0.0740	EEU
CHN_WATKINS-007246	0.0000	0.2643	0.0391	0.2360	0.0242	0.3359	0.1004	EEU
CRO_DAS5-003478	0.0000	0.2809	0.0839	0.3039	0.0198	0.3115	0.0000	EEU
MAC_DAS5-003783	0.1745	0.2893	0.2357	0.0000	0.0000	0.3005	0.0000	EEU
NLD_DAS5-001291	0.0490	0.2551	0.0000	0.1525	0.1960	0.2990	0.0483	EEU
ITA_DAS5-004421	0.0000	0.0000	0.0000	0.9999	0.0000	0.0000	0.0000	EUMED
SPA_WATKINS-007445	0.0000	0.0000	0.0000	0.9999	0.0000	0.0000	0.0000	EUMED
MOR_WATKINS-007651	0.0000	0.0000	0.0000	0.9999	0.0000	0.0000	0.0000	EUMED
TUR_WATKINS-007756	0.0000	0.0000	0.0000	0.9999	0.0000	0.0000	0.0000	EUMED
SPA_WATKINS-007643	0.0000	0.0439	0.0000	0.9319	0.0000	0.0241	0.0000	EUMED
POR_DAS5-001415	0.0000	0.0996	0.0000	0.9004	0.0000	0.0000	0.0000	EUMED
FRA_WATKINS-007316	0.0000	0.0000	0.0000	0.8595	0.0000	0.1405	0.0000	EUMED
IND_WATKINS-007585	0.0806	0.0212	0.0000	0.8356	0.0000	0.0626	0.0000	EUMED
YUG_WATKINS-007295	0.0400	0.0000	0.0000	0.8348	0.0000	0.1252	0.0000	EUMED
BOS_DAS5-003394	0.1902	0.0000	0.0000	0.8097	0.0000	0.0000	0.0000	EUMED
IRN_WATKINS-007691	0.0000	0.0766	0.0212	0.7947	0.0000	0.0978	0.0097	EUMED
CHL_DAS5-001008	0.1178	0.0000	0.0000	0.7789	0.0000	0.1033	0.0000	EUMED
MAC_DAS5-004268	0.0000	0.0000	0.0000	0.7729	0.0000	0.2271	0.0000	EUMED
YUG_WATKINS-007079	0.0000	0.0000	0.0000	0.7724	0.0000	0.2276	0.0000	EUMED
POR_WATKINS-007500	0.0574	0.0000	0.0000	0.7700	0.0000	0.1523	0.0202	EUMED
GEO_DAS5-003574	0.0000	0.0000	0.0000	0.7589	0.0000	0.2411	0.0000	EUMED
BUL_WATKINS-007766	0.0000	0.0903	0.0000	0.7535	0.0000	0.1562	0.0000	EUMED
SPA_WATKINS-007572	0.0000	0.0784	0.0000	0.7531	0.0000	0.1685	0.0000	EUMED
IND_WATKINS-007800	0.0000	0.0990	0.0000	0.7451	0.0000	0.1558	0.0000	EUMED
MAC_DAS5-003778	0.0000	0.0145	0.0380	0.7081	0.0000	0.2394	0.0000	EUMED
BUR_DAS5-001036	0.1490	0.0000	0.0022	0.6955	0.0459	0.0000	0.1074	EUMED
GRE_WATKINS-007088	0.0197	0.3095	0.0000	0.6688	0.0000	0.0019	0.0000	EUMED

Accession	Q1	Q2	Q3	Q4	Q5	Q6	Q7	Subpop
SPA_WATKINS-007449	0.0000	0.1781	0.0000	0.6677	0.0000	0.1542	0.0000	EUMED
SPA_WATKINS-007451	0.0080	0.0523	0.0000	0.6677	0.0076	0.2561	0.0083	EUMED
TUR_WSC-8-6	0.0745	0.0000	0.1200	0.6619	0.0000	0.0000	0.1436	EUMED
ITA_DAS5-004695	0.0785	0.2673	0.0000	0.6400	0.0141	0.0000	0.0000	EUMED
CHN_WATKINS-007248	0.0000	0.0000	0.0000	0.6330	0.0000	0.3614	0.0055	EUMED
MOR_WATKINS-007014	0.0000	0.0000	0.0000	0.6278	0.0000	0.3721	0.0000	EUMED
DEN_DAS5-004333	0.0097	0.0000	0.0040	0.6222	0.0000	0.3641	0.0000	EUMED
SPA_WATKINS-007452	0.0000	0.1280	0.0000	0.6177	0.0000	0.2543	0.0000	EUMED
LEB_DAS5-004337	0.1109	0.2783	0.0000	0.6107	0.0000	0.0000	0.0000	EUMED
FSV_DAS5-002165	0.1218	0.2234	0.0500	0.6048	0.0000	0.0000	0.0000	EUMED
ITA_WATKINS-007423	0.0000	0.2164	0.0000	0.6028	0.0000	0.1807	0.0000	EUMED
GER_DAS5-004694	0.0000	0.0000	0.0000	0.6025	0.0000	0.3974	0.0000	EUMED
PHI_DAS5-001051	0.0715	0.0000	0.2554	0.6020	0.0000	0.0000	0.0712	EUMED
SPA_WATKINS-007684	0.1210	0.0799	0.0577	0.5855	0.0292	0.1105	0.0162	EUMED
OMA_DAS5-001134	0.2996	0.0752	0.0182	0.5615	0.0000	0.0350	0.0105	EUMED
BOS_DAS5-003393	0.0000	0.0000	0.0130	0.5551	0.0000	0.4264	0.0055	EUMED
POR_WSC-8-2	0.0000	0.4474	0.0000	0.5526	0.0000	0.0000	0.0000	EUMED
POR_WATKINS-007494	0.0146	0.3586	0.0000	0.5292	0.0128	0.0000	0.0848	EUMED
POL_DAS5-002870	0.0000	0.4819	0.0000	0.5180	0.0000	0.0000	0.0000	EUMED
AFG_WATKINS-007048	0.0000	0.1949	0.0508	0.5153	0.0000	0.1958	0.0432	EUMED
MOR_DAS5-003849	0.0468	0.3585	0.0166	0.5051	0.0232	0.0000	0.0498	EUMED
IND_WATKINS-007339	0.0062	0.0944	0.1091	0.4980	0.0000	0.1440	0.1483	EUMED
DEN_DAS5-003493	0.0014	0.1002	0.0959	0.4765	0.0659	0.2601	0.0000	EUMED
FSV_DAS5-003243	0.0576	0.0000	0.3848	0.4703	0.0000	0.0746	0.0126	EUMED
YUG_WATKINS-007745	0.1040	0.0007	0.0091	0.4683	0.0037	0.3887	0.0255	EUMED
CHN_WATKINS-007209	0.0000	0.1500	0.2944	0.4587	0.0000	0.0000	0.0969	EUMED
CHN_WATKINS-007260	0.0000	0.0000	0.1238	0.4522	0.0000	0.2307	0.1932	EUMED
BOL_DAS5-003387	0.2825	0.0000	0.1251	0.4433	0.0195	0.0000	0.1296	EUMED
CHN_WATKINS-007202	0.1151	0.3942	0.0000	0.4230	0.0000	0.0678	0.0000	EUMED
BOS_DAS5-004665	0.1196	0.1164	0.0787	0.4190	0.0250	0.1436	0.0977	EUMED
BOS_DAS5-003388	0.0559	0.2453	0.0457	0.4163	0.0000	0.2367	0.0000	EUMED
CHN_WATKINS-007272	0.0506	0.0000	0.1122	0.4153	0.0000	0.1839	0.2379	EUMED
MOR_DAS5-002766	0.2319	0.0000	0.0000	0.3940	0.0000	0.1703	0.2037	EUMED
BEL_DAS5-003370	0.0292	0.0923	0.0353	0.3636	0.3073	0.1140	0.0582	EUMED
CHL_DAS5-004288	0.0000	0.0486	0.0000	0.3585	0.2632	0.3230	0.0067	EUMED
CHN_WATKINS-007256	0.0246	0.0112	0.2391	0.3422	0.0077	0.1385	0.2368	EUMED
CHN_WATKINS-007250	0.0000	0.0000	0.1858	0.3379	0.0000	0.1795	0.2968	EUMED
SPA_DAS5-004493	0.0000	0.2623	0.0000	0.3210	0.0551	0.2811	0.0804	EUMED
BEL_DAS5-004418	0.0000	0.0250	0.1047	0.3200	0.2842	0.1904	0.0757	EUMED
YEM_DAS5-004245	0.2321	0.0862	0.2769	0.3181	0.0000	0.0000	0.0866	EUMED
LEB_DAS5-001551	0.2572	0.1030	0.1515	0.2996	0.0344	0.0432	0.1111	EUMED

Accession	Q1	Q2	Q3	Q4	Q5	Q6	Q7	Subpop
TUR_DAS5-002287	0.0000	0.0000	0.0000	0.0000	0.9999	0.0000	0.0000	FSV
FSV_DAS5-002515	0.0000	0.0000	0.0000	0.0000	0.9999	0.0000	0.0000	FSV
ITA_DAS5-002933	0.0000	0.0000	0.0000	0.0000	0.9999	0.0000	0.0000	FSV
MON_DAS5-004285	0.0000	0.0000	0.0000	0.0000	0.9999	0.0000	0.0000	FSV
FSV_DAS5-002303	0.0000	0.0000	0.0000	0.0000	0.9999	0.0000	0.0000	FSV
GER_WATKINS-007811	0.0000	0.0000	0.0084	0.0000	0.8863	0.0000	0.1053	FSV
SPA_DAS5-002916	0.0000	0.1007	0.0000	0.0000	0.8489	0.0503	0.0000	FSV
YEM_DAS5-004244	0.0138	0.0000	0.1216	0.0000	0.7776	0.0000	0.0869	FSV
SPA_DAS5-004007	0.0000	0.0000	0.0000	0.2668	0.6978	0.0000	0.0353	FSV
ETH_DAS5-001932	0.0000	0.0000	0.2197	0.0000	0.6201	0.0308	0.1294	FSV
POR_DAS5-004676	0.0000	0.3695	0.0000	0.0889	0.4841	0.0351	0.0224	FSV
SPA_DAS5-004018	0.0098	0.0988	0.0997	0.2723	0.4330	0.0397	0.0466	FSV
POR_WATKINS-007497	0.0000	0.3449	0.0000	0.1849	0.4212	0.0075	0.0415	FSV
CHN_WATKINS-007212	0.0000	0.0000	0.0000	0.3945	0.3966	0.1542	0.0547	FSV
SER_DAS5-003993	0.0164	0.0000	0.1431	0.2645	0.3194	0.2015	0.0551	FSV
USA_DAS5-004691	0.0000	0.0000	0.1341	0.2733	0.3162	0.1925	0.0838	FSV
ALG_DAS5-001609	0.0000	0.9999	0.0000	0.0000	0.0000	0.0000	0.0000	MED
ALG_WATKINS-007122	0.0000	0.9999	0.0000	0.0000	0.0000	0.0000	0.0000	MED
SPA_WATKINS-007443	0.0000	0.9999	0.0000	0.0000	0.0000	0.0000	0.0000	MED
AFG_WATKINS-007117	0.0000	0.9634	0.0000	0.0365	0.0000	0.0000	0.0000	MED
TUN_WATKINS-007035	0.0000	0.9472	0.0000	0.0528	0.0000	0.0000	0.0000	MED
ISR_DAS5-003704	0.0000	0.9161	0.0544	0.0000	0.0295	0.0000	0.0000	MED
ISR_DAS5-001203	0.0000	0.8905	0.0000	0.0000	0.1094	0.0000	0.0000	MED
TUR_WATKINS-007030	0.0000	0.8894	0.0071	0.0000	0.0000	0.0953	0.0082	MED
GRE_WATKINS-007090	0.0000	0.8698	0.0000	0.0000	0.1301	0.0000	0.0000	MED
SPA_WATKINS-007127	0.0000	0.8534	0.0250	0.0345	0.0000	0.0871	0.0000	MED
SYR_WATKINS-007009	0.0000	0.8404	0.1596	0.0000	0.0000	0.0000	0.0000	MED
SPA_WATKINS-007187	0.0000	0.8310	0.0191	0.0000	0.1101	0.0156	0.0241	MED
CYP_WSC-7-4	0.0000	0.8296	0.0570	0.0000	0.1134	0.0000	0.0000	MED
PRK_WSC-8-1	0.0000	0.8279	0.0000	0.1151	0.0570	0.0000	0.0000	MED
MOR_DAS5-003835	0.0196	0.8247	0.0000	0.0000	0.0000	0.0000	0.1557	MED
CYP_WATKINS-007826	0.0000	0.8138	0.1557	0.0000	0.0000	0.0305	0.0000	MED
SPA_DAS5-004012	0.0000	0.8128	0.0000	0.0004	0.0000	0.1868	0.0000	MED
SPA_WATKINS-007188	0.0000	0.7870	0.0072	0.0085	0.1381	0.0561	0.0032	MED
MEX_Cltr-2804	0.0016	0.7738	0.0213	0.0931	0.0462	0.0639	0.0000	MED
GRE_DAS5-001641	0.0318	0.7715	0.0255	0.0277	0.0814	0.0621	0.0000	MED
CYP_DAS5-003480	0.0000	0.7626	0.1406	0.0328	0.0000	0.0640	0.0000	MED
KEN_DAS5-001809	0.0000	0.7613	0.0000	0.0000	0.2169	0.0000	0.0218	MED
MOR_DAS5-004265	0.0000	0.6941	0.2095	0.0000	0.0443	0.0000	0.0521	MED
SPA_WATKINS-007677	0.0000	0.6906	0.0000	0.1040	0.0000	0.2053	0.0000	MED
SPA_DAS5-001212	0.0000	0.6781	0.0244	0.0715	0.2004	0.0256	0.0000	MED

Accession	Q1	Q2	Q3	Q4	Q5	Q6	Q7	Subpop
GRE_WSC-7-3	0.0000	0.6702	0.0000	0.2762	0.0296	0.0239	0.0000	MED
ECU_DAS5-003501	0.0000	0.6445	0.0690	0.0000	0.2739	0.0000	0.0126	MED
TUN_DAS5-001935	0.0000	0.6374	0.1644	0.0170	0.0434	0.1378	0.0000	MED
GRE_WATKINS-007002	0.0844	0.6350	0.0000	0.1439	0.0000	0.0163	0.1204	MED
SPA_WATKINS-007558	0.0000	0.6283	0.0230	0.2558	0.0000	0.0928	0.0000	MED
GRE_WATKINS-007077	0.0000	0.6025	0.0000	0.3142	0.0000	0.0833	0.0000	MED
POR_DAS5-001924	0.0036	0.5997	0.0000	0.2701	0.1265	0.0000	0.0000	MED
EGY_WATKINS-007542	0.0492	0.5970	0.0068	0.1980	0.0000	0.1489	0.0000	MED
SPA_DAS5-004020	0.0000	0.5768	0.0000	0.3968	0.0264	0.0000	0.0000	MED
MOR_DAS5-004663	0.0000	0.5725	0.0675	0.0000	0.1320	0.1866	0.0414	MED
TUN_WATKINS-007799	0.0000	0.5721	0.0519	0.1570	0.1036	0.0459	0.0695	MED
AUS_WATKINS-007156	0.0412	0.5626	0.0000	0.3721	0.0000	0.0241	0.0000	MED
PER_DAS5-004698	0.0000	0.5533	0.0000	0.0947	0.3450	0.0000	0.0070	MED
ANG_WSC-8-8	0.0552	0.5416	0.0000	0.2212	0.0000	0.0000	0.1820	MED
GRE_WATKINS-007093	0.0000	0.5400	0.0000	0.4600	0.0000	0.0000	0.0000	MED
BRA_WSC-8-9	0.0410	0.5362	0.0000	0.4199	0.0029	0.0000	0.0000	MED
TUR_WSC-8-4	0.0000	0.5327	0.3580	0.0000	0.0776	0.0317	0.0000	MED
URU_PI-43355	0.0000	0.5252	0.0000	0.4748	0.0000	0.0000	0.0000	MED
GRE_WATKINS-007001	0.0000	0.5170	0.0000	0.4805	0.0024	0.0000	0.0000	MED
SPA_WATKINS-007455	0.0000	0.5140	0.0000	0.3997	0.0000	0.0862	0.0000	MED
ANG_DAS5-001276	0.0303	0.5124	0.0000	0.2518	0.0064	0.0000	0.1991	MED
GUA_WSC-8-3	0.0000	0.5010	0.0286	0.1779	0.1130	0.1796	0.0000	MED
TUN_DAS5-001399	0.2864	0.4849	0.0000	0.0000	0.1659	0.0000	0.0628	MED
TUR_DAS5-004483	0.0000	0.4814	0.3385	0.0000	0.1354	0.0446	0.0000	MED
SPA_WATKINS-007520	0.0000	0.4755	0.0000	0.2404	0.0000	0.2841	0.0000	MED
CRO_DAS5-004316	0.0000	0.4659	0.0000	0.2621	0.0000	0.2720	0.0000	MED
IRQ_WATKINS-007641	0.1140	0.4578	0.0000	0.1795	0.0000	0.2487	0.0000	MED
FSV_DAS5-003176	0.0267	0.4516	0.0000	0.4207	0.0170	0.0839	0.0000	MED
GUA_DAS5-003601	0.0000	0.4479	0.0965	0.0735	0.2841	0.0872	0.0107	MED
GUA_DAS5-004664	0.0647	0.4472	0.1648	0.1097	0.1025	0.1110	0.0000	MED
AUS_WATKINS-007148	0.0000	0.4360	0.0533	0.2006	0.0000	0.3101	0.0000	MED
IRQ_WATKINS-007638	0.0000	0.4321	0.1440	0.4224	0.0015	0.0000	0.0000	MED
SYR_DAS5-004059	0.0000	0.4259	0.3965	0.0000	0.0000	0.1776	0.0000	MED
CYP_DAS5-001320	0.1205	0.4157	0.1056	0.2671	0.0911	0.0000	0.0000	MED
IND_WATKINS-007388	0.0103	0.4135	0.0882	0.1005	0.0000	0.1338	0.2538	MED
CHN_WATKINS-007261	0.0232	0.3776	0.0714	0.2996	0.0061	0.1253	0.0969	MED
SPA_WATKINS-007430	0.0000	0.3635	0.1025	0.2845	0.0000	0.2494	0.0000	MED
VEN_DAS5-001433	0.2169	0.2351	0.1583	0.1191	0.0734	0.0681	0.1291	MED
BOL_DAS5-001094	0.0000	0.0000	0.9999	0.0000	0.0000	0.0000	0.0000	OLDWP
TKM_WSC-7-1	0.0000	0.0000	0.9999	0.0000	0.0000	0.0000	0.0000	OLDWP
CHA_DAS5-001251	0.0000	0.0000	0.9919	0.0000	0.0080	0.0000	0.0000	OLDWP

Accession	Q1	Q2	Q3	Q4	Q5	Q6	Q7	Subpop
TAN_DAS5-001205	0.0000	0.0168	0.9736	0.0000	0.0096	0.0000	0.0000	OLDWP
AUT_DAS5-001116	0.0000	0.0273	0.9726	0.0000	0.0000	0.0000	0.0000	OLDWP
AFG_PI-268466	0.0000	0.0000	0.9625	0.0000	0.0298	0.0076	0.0000	OLDWP
IRQ_WSC-7-8	0.0000	0.0072	0.9475	0.0000	0.0185	0.0267	0.0000	OLDWP
FSV_WATKINS-007827	0.0000	0.0000	0.9349	0.0000	0.0000	0.0000	0.0651	OLDWP
NIG_DAS5-003881	0.0000	0.0575	0.9330	0.0000	0.0095	0.0000	0.0000	OLDWP
IRQ_DAS5-003689	0.0000	0.0000	0.9288	0.0185	0.0101	0.0000	0.0425	OLDWP
IRN_WATKINS-007702	0.0000	0.0000	0.9139	0.0000	0.0000	0.0000	0.0860	OLDWP
UZB_DAS5-004240	0.0000	0.0000	0.9128	0.0000	0.0000	0.0000	0.0871	OLDWP
FSV_WATKINS-007789	0.0000	0.0000	0.8972	0.0000	0.0000	0.1027	0.0000	OLDWP
UZB_DAS5-001540	0.0000	0.0000	0.8919	0.0107	0.0000	0.0280	0.0695	OLDWP
AFG_DAS5-003288	0.0000	0.0591	0.8696	0.0000	0.0000	0.0000	0.0713	OLDWP
AFG_WATKINS-007049	0.0000	0.0000	0.8603	0.0000	0.0000	0.0000	0.1397	OLDWP
IRQ_DAS5-001458	0.0000	0.0452	0.8538	0.0000	0.0211	0.0799	0.0000	OLDWP
ISR_PI-94567	0.0000	0.0000	0.8532	0.0000	0.0000	0.0000	0.1468	OLDWP
AFG_DAS5-004583	0.0000	0.0000	0.8518	0.0000	0.0000	0.0000	0.1482	OLDWP
IRN_WATKINS-007708	0.0000	0.0000	0.8456	0.0309	0.0000	0.0299	0.0936	OLDWP
FSV_WATKINS-007547	0.0000	0.0000	0.8327	0.0000	0.0000	0.0000	0.1673	OLDWP
UZB_DAS5-001984	0.0000	0.0000	0.8175	0.0000	0.0000	0.0000	0.1824	OLDWP
AFG_WSC-4-2	0.0000	0.0000	0.8169	0.0000	0.0000	0.0000	0.1831	OLDWP
UZB_PI-9791	0.0000	0.0000	0.8072	0.0000	0.0000	0.0000	0.1928	OLDWP
IRN_WATKINS-007470	0.0238	0.0000	0.8056	0.0000	0.0000	0.1537	0.0169	OLDWP
YEM_DAS5-004253	0.0024	0.0658	0.7913	0.0130	0.0247	0.0025	0.1003	OLDWP
IRN_DAS5-003654	0.0000	0.0000	0.7913	0.0000	0.0000	0.0000	0.2087	OLDWP
IRN_DAS5-003641	0.0000	0.0820	0.7832	0.0000	0.0000	0.1348	0.0000	OLDWP
TUN_DAS5-004072	0.0000	0.1426	0.7789	0.0000	0.0785	0.0000	0.0000	OLDWP
ETH_WSC-7-6	0.0000	0.0612	0.7745	0.0000	0.0427	0.1001	0.0214	OLDWP
FSV_WATKINS-007137	0.0053	0.0000	0.7681	0.0000	0.0000	0.2265	0.0000	OLDWP
ARM_DAS5-003308	0.0430	0.0150	0.7655	0.0000	0.0000	0.1764	0.0000	OLDWP
YEM_DAS5-001244	0.1193	0.0000	0.7642	0.0742	0.0000	0.0000	0.0423	OLDWP
YEM_DAS5-004256	0.0000	0.0705	0.7599	0.0000	0.0388	0.0000	0.1308	OLDWP
PAK_Cltr-15134	0.0000	0.0000	0.7596	0.0000	0.0000	0.0000	0.2403	OLDWP
SUD_WSC-4-5	0.0064	0.0000	0.7454	0.0518	0.0007	0.0000	0.1958	OLDWP
FSV_WATKINS-007548	0.0452	0.0545	0.7452	0.0000	0.0000	0.1550	0.0000	OLDWP
FSV_WATKINS-007194	0.0000	0.1247	0.7452	0.0000	0.0000	0.1301	0.0000	OLDWP
IRN_DAS5-003660	0.0122	0.0000	0.7448	0.0607	0.0000	0.0026	0.1797	OLDWP
IND_WATKINS-007581	0.0000	0.0135	0.7436	0.0000	0.0608	0.0719	0.1102	OLDWP
AFG_DAS5-003279	0.0000	0.0000	0.7330	0.0000	0.0000	0.0000	0.2669	OLDWP
AZE_DAS5-004465	0.0578	0.0284	0.7316	0.0000	0.0000	0.1822	0.0000	OLDWP
AFG_DAS5-004674	0.0000	0.0000	0.7293	0.0000	0.0000	0.0000	0.2707	OLDWP
ETH_DAS5-003524	0.0310	0.1053	0.7262	0.0000	0.0395	0.0980	0.0000	OLDWP

Accession	Q1	Q2	Q3	Q4	Q5	Q6	Q7	Subpop
AFG_WSC-4-9	0.0000	0.0000	0.7213	0.0038	0.0000	0.0000	0.2748	OLDWP
AZE_WATKINS-007784	0.1301	0.0000	0.6740	0.0551	0.0072	0.1179	0.0158	OLDWP
GEO_DAS5-003570	0.0432	0.0278	0.6735	0.0000	0.0000	0.2555	0.0000	OLDWP
HON_DAS5-001079	0.0000	0.1994	0.6590	0.0516	0.0245	0.0000	0.0655	OLDWP
FSV_WATKINS-007191	0.0509	0.0186	0.6437	0.0000	0.0000	0.2867	0.0000	OLDWP
ERI_DAS5-001368	0.0505	0.1365	0.6013	0.0000	0.0305	0.1636	0.0177	OLDWP
IRN_DAS5-003661	0.0000	0.2281	0.5992	0.0000	0.0000	0.1726	0.0000	OLDWP
CHN_WATKINS-007220	0.0000	0.0000	0.5518	0.0000	0.0000	0.0000	0.4481	OLDWP
SAU_DAS5-003980	0.0116	0.3059	0.5269	0.0000	0.0079	0.1441	0.0036	OLDWP
TAJ_DAS5-001916	0.0000	0.0000	0.5225	0.0000	0.0000	0.0000	0.4775	OLDWP
BUR_DAS5-001521	0.0000	0.0000	0.5192	0.0000	0.0000	0.0000	0.4807	OLDWP
ALG_DAS5-004460	0.0000	0.3720	0.5176	0.0000	0.0923	0.0000	0.0181	OLDWP
RUS_Cltr-7635	0.1234	0.0517	0.5162	0.0000	0.0000	0.3086	0.0000	OLDWP
IRA_WSC-7-2	0.0000	0.2847	0.5137	0.0000	0.0000	0.2016	0.0000	OLDWP
ALG_WSC-5-12	0.0000	0.3350	0.4995	0.0000	0.1001	0.0000	0.0654	OLDWP
IND_WATKINS-007401	0.0314	0.0000	0.4853	0.1169	0.0050	0.0913	0.2702	OLDWP
FSV_DAS5-003170	0.0000	0.0000	0.4851	0.1930	0.0000	0.2720	0.0498	OLDWP
POL_DAS5-002871	0.0841	0.0402	0.4581	0.3308	0.0000	0.0868	0.0000	OLDWP
TKM_DAS5-001564	0.0000	0.2256	0.4545	0.0000	0.0000	0.3199	0.0000	OLDWP
SPA_WATKINS-007661	0.0000	0.0916	0.4099	0.3969	0.0000	0.1016	0.0000	OLDWP
IRN_DAS5-003670	0.0000	0.3134	0.4072	0.0000	0.0000	0.2794	0.0000	OLDWP
GEO_WATKINS-007824	0.1198	0.1571	0.4050	0.0000	0.0000	0.3181	0.0000	OLDWP
IRQ_WATKINS-007640	0.0793	0.3177	0.3975	0.0128	0.0564	0.1034	0.0331	OLDWP
ROM_DAS5-002944	0.0000	0.1044	0.3836	0.1723	0.0000	0.3397	0.0000	OLDWP
FSV_DAS5-002492	0.1198	0.2024	0.3804	0.0793	0.0000	0.1444	0.0736	OLDWP
CRO_Cltr-11223	0.1757	0.0272	0.3798	0.0000	0.0000	0.4173	0.0000	OLDWP
GEO_DAS5-003577	0.0000	0.1950	0.3460	0.0877	0.2209	0.0796	0.0708	OLDWP
BUR_DAS5-001007	0.0000	0.0000	0.0000	0.0000	0.0000	0.0000	0.9999	SAS
PAK_DAS5-001341	0.0000	0.0000	0.0000	0.0000	0.0000	0.0000	0.9999	SAS
PAR_DAS5-001524	0.0000	0.0000	0.0000	0.0000	0.0000	0.0000	0.9999	SAS
IND_DAS5-001550	0.0000	0.0000	0.0000	0.0000	0.0000	0.0000	0.9999	SAS
IDN_DAS5-001690	0.0000	0.0000	0.0000	0.0000	0.0000	0.0000	0.9999	SAS
ANG_DAS5-001803	0.0000	0.0000	0.0000	0.0000	0.0000	0.0000	0.9999	SAS
NEP_WSC-4-3	0.0000	0.0000	0.0000	0.0000	0.0000	0.0000	0.9999	SAS
IRQ_DAS5-003690	0.0000	0.0000	0.0000	0.0000	0.0000	0.0000	0.9999	SAS
MWI_PI-61693	0.0000	0.0000	0.0172	0.0000	0.0000	0.0000	0.9827	SAS
NEP_WSC-5-13	0.0000	0.0000	0.0352	0.0000	0.0000	0.0439	0.9209	SAS
JOR_PI-283147	0.0783	0.0699	0.0000	0.0000	0.0000	0.0000	0.8518	SAS
BHU_WSC-4-6	0.0000	0.0000	0.1938	0.0000	0.0000	0.0000	0.8061	SAS
OMA_DAS5-001657	0.0000	0.0000	0.2071	0.0000	0.0000	0.0000	0.7928	SAS
IND_WATKINS-007606	0.0000	0.1247	0.0000	0.0378	0.0000	0.0903	0.7471	SAS

Accession	Q1	Q2	Q3	Q4	Q5	Q6	Q7	Subpop
NEP_DAS5-002811	0.0000	0.0000	0.2590	0.0000	0.0000	0.0000	0.7410	SAS
MLI_DAS5-001100	0.2901	0.0000	0.0000	0.0000	0.0000	0.0000	0.7098	SAS
IND_WSC-8-5	0.3003	0.0000	0.0000	0.0000	0.0000	0.0000	0.6997	SAS
IND_WATKINS-007398	0.0930	0.1014	0.0000	0.0000	0.0000	0.1196	0.6860	SAS
EGY_PI-220431	0.3026	0.0172	0.0000	0.0000	0.0000	0.0000	0.6802	SAS
CHN_DAS5-003455	0.0000	0.0000	0.4530	0.0000	0.0000	0.0000	0.5469	SAS
ROM_WATKINS-007510	0.0000	0.0000	0.4594	0.0000	0.0000	0.0000	0.5406	SAS
IND_WATKINS-007350	0.0150	0.1783	0.0434	0.2262	0.0000	0.0000	0.5370	SAS
UNK_DAS5-004209	0.0000	0.0000	0.4483	0.0169	0.0000	0.0000	0.5347	SAS
PHI_Cltr-4175	0.0000	0.0000	0.4703	0.0000	0.0000	0.0000	0.5296	SAS
CHN_WATKINS-007263	0.0167	0.0006	0.3911	0.0368	0.0278	0.0048	0.5220	SAS
CHN_WATKINS-007210	0.0000	0.0000	0.4893	0.0000	0.0000	0.0000	0.5106	SAS
SUD_DAS5-001470	0.1662	0.0599	0.0000	0.0000	0.2822	0.0000	0.4917	SAS
IND_WATKINS-007395	0.0000	0.0000	0.0000	0.4233	0.0000	0.1137	0.4630	SAS
MEX_DAS5-002004	0.2566	0.1042	0.0000	0.0976	0.1608	0.0000	0.3809	SAS
IND_WATKINS-007383	0.0000	0.0239	0.1142	0.2377	0.0000	0.2513	0.3730	SAS
TUN_WATKINS-007759	0.2244	0.2523	0.0000	0.0360	0.0000	0.1547	0.3326	SAS
BEL_DAS5-003360	0.1005	0.0437	0.0610	0.2134	0.1760	0.1084	0.2970	SAS
MOZ_DAS5-003855	0.9999	0.0000	0.0000	0.0000	0.0000	0.0000	0.0000	WLDMix
SPA_WATKINS-007454	0.9999	0.0000	0.0000	0.0000	0.0000	0.0000	0.0000	WLDMix
BOL_WSC-7-9	0.9999	0.0000	0.0000	0.0000	0.0000	0.0000	0.0000	WLDMix
AUS_WATKINS-007166	0.8826	0.0000	0.0000	0.0000	0.0000	0.0000	0.1174	WLDMix
TUN_DAS5-004078	0.8735	0.0000	0.0000	0.0000	0.0000	0.0000	0.1264	WLDMix
POL_DAS5-002868	0.8385	0.0201	0.1041	0.0000	0.0000	0.0373	0.0000	WLDMix
MOR_DAS5-003838	0.8236	0.0000	0.0000	0.0293	0.0000	0.0000	0.1471	WLDMix
AUS_WATKINS-007162	0.8022	0.0000	0.0000	0.0000	0.0000	0.0000	0.1977	WLDMix
CHN_WATKINS-007274	0.7000	0.0000	0.0000	0.0000	0.0000	0.3000	0.0000	WLDMix
LIB_PI-54431	0.6879	0.0792	0.0000	0.0000	0.0000	0.0000	0.2328	WLDMix
FSV_DAS5-003553	0.6436	0.1405	0.0000	0.2158	0.0000	0.0000	0.0000	WLDMix
GEO_PI-572692	0.6428	0.0000	0.0000	0.0862	0.1176	0.1254	0.0279	WLDMix
YEM_DAS5-004257	0.6381	0.0000	0.0025	0.1663	0.0000	0.0392	0.1538	WLDMix
CHN_PI-70613	0.6296	0.0000	0.0000	0.0000	0.0000	0.3703	0.0000	WLDMix
RUS_DAS5-003961	0.6274	0.0000	0.0000	0.0000	0.0000	0.3726	0.0000	WLDMix
ERI_DAS5-001365	0.6100	0.0119	0.0945	0.0000	0.0679	0.0968	0.1189	WLDMix
SWE_WSC-7-5	0.6001	0.1851	0.0000	0.0000	0.0000	0.0000	0.2148	WLDMix
POL_DAS5-002869	0.5945	0.0000	0.0000	0.0000	0.0000	0.4055	0.0000	WLDMix
COL_DAS5-003469	0.5741	0.0000	0.0000	0.2871	0.0000	0.0000	0.1388	WLDMix
IND_WATKINS-007604	0.5709	0.0000	0.0000	0.0000	0.0000	0.4290	0.0000	WLDMix
SAU_Cltr-15144	0.5469	0.0000	0.0000	0.0000	0.0107	0.0000	0.4424	WLDMix
SPA_WATKINS-007518	0.5434	0.0000	0.0000	0.0000	0.4565	0.0000	0.0000	WLDMix
EGY_DAS5-003502	0.5392	0.0000	0.0000	0.0000	0.0048	0.0000	0.4559	WLDMix

Accession	Q1	Q2	Q3	Q4	Q5	Q6	Q7	Subpop
COL_DAS5-003468	0.5363	0.0000	0.0026	0.3327	0.0147	0.0000	0.1138	WLDMix
Bakhatwa	0.5251	0.0622	0.0082	0.2362	0.0235	0.0000	0.1449	WLDMix
CAN_DAS5-001453	0.5075	0.0000	0.0000	0.0112	0.0000	0.1655	0.3158	WLDMix
PER_WSC-5-7	0.5051	0.0329	0.0000	0.3327	0.0097	0.0000	0.1195	WLDMix
EGY_DAS5-001149	0.5007	0.0000	0.0000	0.0227	0.0142	0.0000	0.4625	WLDMix
SAU_DAS5-003977	0.4934	0.0000	0.0000	0.3364	0.0000	0.0000	0.1701	WLDMix
SWI_WSC-4-4	0.4873	0.0378	0.0000	0.0843	0.1785	0.0000	0.2121	WLDMix
ISR_DAS5-003707	0.4764	0.0593	0.0130	0.1035	0.0000	0.0000	0.3478	WLDMix
FSV_WATKINS-007192	0.4412	0.3363	0.0000	0.0063	0.0000	0.0000	0.2163	WLDMix
ANG_DAS5-001265	0.4367	0.1962	0.0009	0.0559	0.1491	0.0000	0.1611	WLDMix
UNK_DAS5-004200	0.4314	0.0108	0.0029	0.1452	0.0000	0.2831	0.1266	WLDMix
ARM_DAS5-003301	0.4268	0.0000	0.1108	0.2588	0.0375	0.0017	0.1643	WLDMix
BUR_DAS5-003428	0.4240	0.0368	0.0702	0.1941	0.1254	0.0000	0.1496	WLDMix
POR_DAS5-001382	0.4168	0.2887	0.0000	0.0907	0.1511	0.0000	0.0525	WLDMix
JOR_DAS5-003735	0.3967	0.0515	0.0000	0.0000	0.2361	0.1040	0.2116	WLDMix
AUS_WATKINS-007155	0.3933	0.0000	0.0000	0.2319	0.0000	0.3672	0.0076	WLDMix
ETH_DAS5-001163	0.3728	0.0572	0.3050	0.0989	0.0146	0.1012	0.0503	WLDMix
JAP_WSC-4-8	0.3503	0.2009	0.0000	0.1103	0.2057	0.0741	0.0587	WLDMix
IND_DAS5-001047	0.3354	0.0371	0.2174	0.2112	0.0000	0.1989	0.0000	WLDMix
BUR_DAS5-001002	0.3192	0.1279	0.0000	0.1903	0.1932	0.0000	0.1694	WLDMix
ARG_DAS5-004653	0.3178	0.1304	0.0357	0.3352	0.0465	0.0000	0.1344	WLDMix

Accession: the first three letters indicate the United Nations country naming code from where the accession originated followed by accession name.

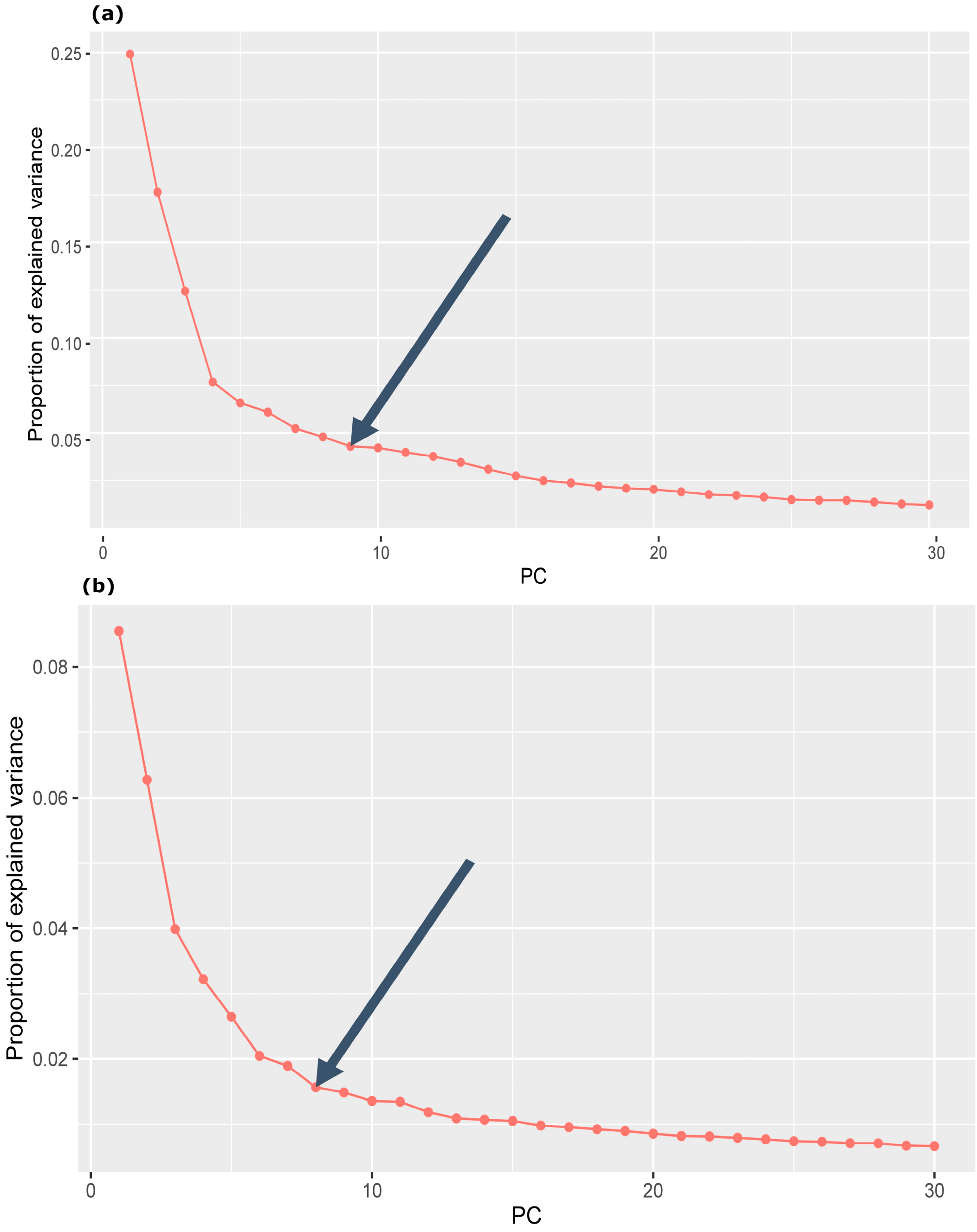

Figure S1. Scree plots generated to estimate the appropriate number of ancestral populations; (a) main population (n=921); (b) landraces (n=347). The arrow indicates the point where the curve starts a horizontal trend. The appropriate number of ancestral populations is the point to the left of the point indicated by the arrow.

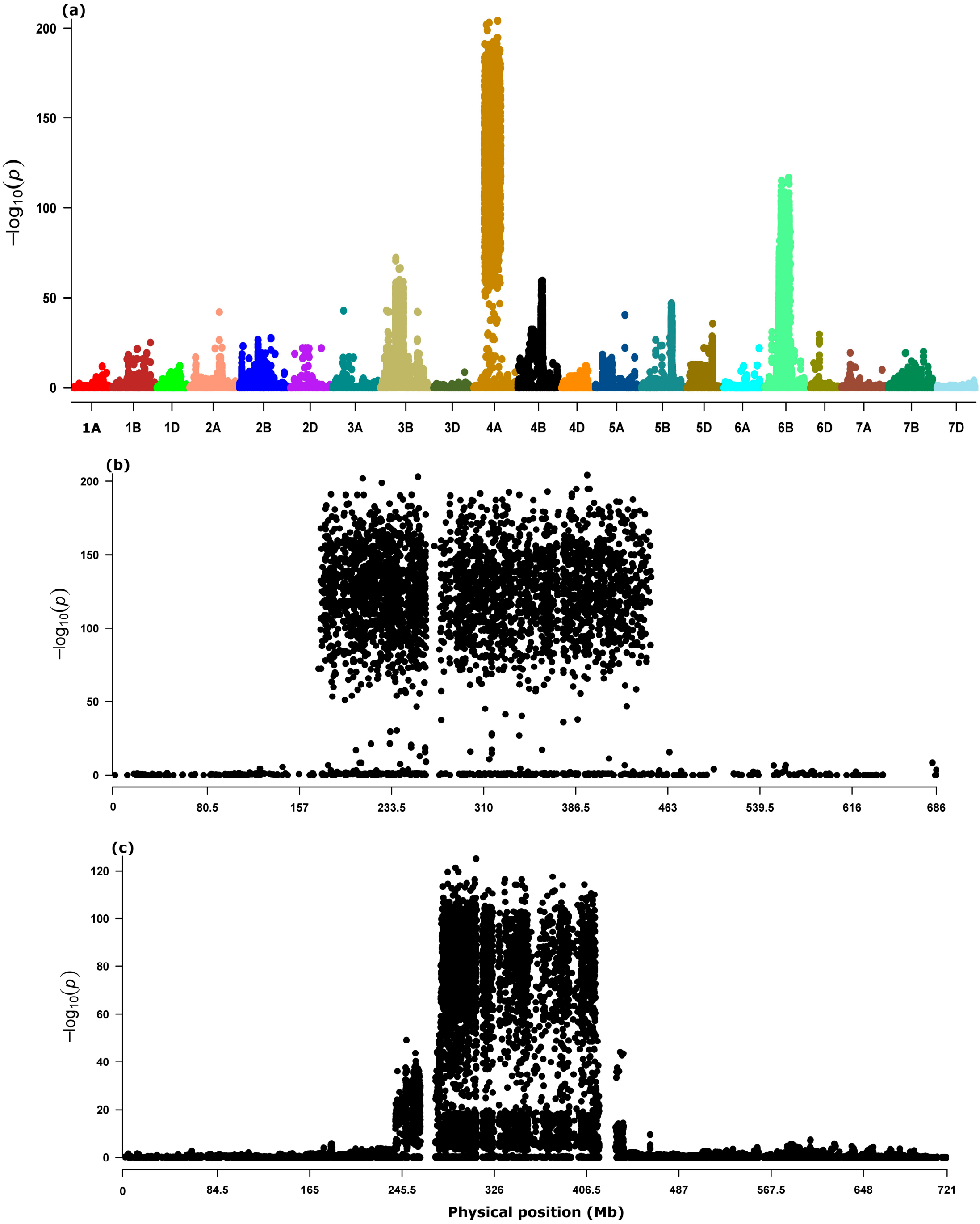

Figure S2. Manhattan plots showing genomic regions indicating past hybridization and/or adaptation signatures generated through pc-based genomes scanning of whole genome resequencing genotype data of hexaploid wheats; (a) Manhattan plot showing entire genome; (b) and (c) Manhattans showing the peak regions on chromosome 4A and 6B respectively.

Table S3. Haplotypes carried by at least two individuals based on 62 highly differentiated SNPs.

Hap[1]	Haplotype sequence	#Hap[2]	Ind[3]	Freq[4]	%[5]	Group[6]
HAP1	ATCGGGTCTTTATGCGTAGGGTGATGGTAGTCCACAAGTTCAGGTTAGTGCCGACGTTCTCA	1120	560	0.608	100.0	G1
HAP2	GATAATGTCCCTCATCCGTAACCCCTAACAATGCGCCTGCTCAACCGAGAAGCTTAGCTCTG	230	115	0.125	100.0	G2
HAP3	GATAATGTCCCTCATCCGTGACCCCTAACAATGCGCCTGCTCAACCGAGAAGCTTAGCTCTG	47	24	0.026	98.4	G2
HAP4	ATCGGGTCTTTATGCGTAGGGTGATGGTCGTCCACAAGTTCAGGTTAGTGCCGACGTTCTCA	11	6	0.006	98.4	G1
HAP5	ATCGGGTCCTTATGCGTAGGGTGATGGTAGTCCACAAGTTCAGGTTAGTGCCGACGTTCTCA	11	6	0.006	98.4	G1
HAP6	ATCGGGTCTTTATGCCTAGGGTGATGGTAGTCCACAAGTTCAGGTTAGTGCCGACGTTCTCA	9	5	0.005	98.4	G1
HAP7	ATCGGGTCTTTATGCGTAGGGTGATGGAAGTCCACAAGTTCAGGTTAGTGCCGACGTTCTCA	9	5	0.005	98.4	G1
HAP8	ATCGGGTCTTTATGTGTAGGGTGATGGTAGTCCACAAGTTCAGGTTAGTGCCGACGTTCTCA	8	4	0.004	98.4	G1
HAP9	ATCGGTTCTTTATGCGTAGGGTGATGGTAGTCCACAAGTTCAGGTTAGTGCCGACGTTCTCA	5	3	0.003	98.4	G1
HAP10	AACGGGTCTTTATGCGTAGGGTGATGGTAGTCCACAAGTTCAGGTTAGTGCCGACGTTCTCA	4	2	0.002	98.4	G1
HAP11	GATAATGTCCCTCATCCGTGGCCCCTAACAATGCGCCTGCTCAACCGAGAAGCTTAGCTCTG	4	2	0.002	96.8	G2
HAP12	GATAATGTCCCTCATCCGTAACCCCTAACAATGCGCCTTCTCAACCGAGAAGCTTAGCTCTG	4	2	0.002	98.4	G2
HAP13	ATCAGGTCTTTATGCGTAGGGTGATGGTAGTCCACAAGTTCAGGTTAGTGCCGACGTTCTCA	4	2	0.002	98.4	G1
HAP14	ATCGGGTCTTTATGCGTAGGGTGATGGTAGTCCCCAAGTTCAGGTTAGTGCCGACGTTCTCA	4	2	0.002	98.4	G1
HAP15	GATAATGTCCCTCATCCGTGACCCCTAACAATGCGCCTGCTCAACCGAGAAGCTTAGCTCTA	4	2	0.002	98.4	G2

[1]Haplotype names; [2]Total number of haplotype detected; [3]The minimum number of individuals carrying the haplotype; [4]Haplotype frequency against the total number of detected haplotypes (n=1842) or individuals carrying the haplotype per the total number of individuals (n=921); [5] Percent sequence similarity to HAP1 or HAP2; [6]Haplotypes falling under G1= Group1 (over 95% similarity with HAP1) or G2= Group2 (over 95% similarity with HAP2).

Table S4. High confidence genes within the elevated 4A region.

GeneID	Gene2	gene_start_query	gene_end_query	Description
TraesCS4A02G151200	1	3.03E+08	302681173	GRF1-interacting factor-like protein
TraesCS4A02G151300	1	3.03E+08	302721227	DNA-directed RNA polymerase I subunit rpa49
TraesCS4A02G151500	1	3.06E+08	306320710	Nuclear factor Y subunit C
TraesCS4A02G151600	1	3.07E+08	307089077	P-loop containing nucleoside triphosphate hydrolases superfamily protein
TraesCS4A02G151700	4	3.08E+08	308058892	Ycf68
TraesCS4A02G151800	1	3.09E+08	309090743	Protein transport protein SEC23
TraesCS4A02G151900	1	3.09E+08	309286089	ATP synthase subunit beta
TraesCS4A02G152000	1	3.09E+08	309286830	ATP synthase epsilon chain
TraesCS4A02G152100	1	3.09E+08	309289160	NAD(P)H-quinone oxidoreductase subunit 3, chloroplastic
TraesCS4A02G152200	1	3.09E+08	309375435	NAD(P)H-quinone oxidoreductase subunit K, chloroplastic
TraesCS4A02G152300	1	3.09E+08	309376016	NAD(P)H-quinone oxidoreductase subunit J, chloroplastic
TraesCS4A02G152400	1	3.09E+08	309446750	ATP synthase subunit c, chloroplastic
TraesCS4A02G152500	1	3.09E+08	309448062	ATP synthase subunit a, chloroplastic
TraesCS4A02G152600	3	3.09E+08	309447586	Cytochrome c-550
TraesCS4A02G152700	1	3.09E+08	309449027	30S ribosomal protein S2
TraesCS4A02G152800	1	3.09E+08	309478441	DNA-directed RNA polymerase subunit beta
TraesCS4A02G152900	1	3.09E+08	309478882	myo-inositol oxygenase 5
TraesCS4A02G153000	2	3.09E+08	309489583	Photosystem II CP43 reaction center protein
TraesCS4A02G153100	2	3.1E+08	309504713	Photosystem II protein D2
TraesCS4A02G153200	2	3.1E+08	309506404	Photosystem II reaction center protein I
TraesCS4A02G153300	5	3.1E+08	309511422	Maturase K
TraesCS4A02G153400	2	3.1E+08	309513620	Photosystem II protein D1
TraesCS4A02G153500	1	3.1E+08	309513944	30S ribosomal protein S19, chloroplastic
TraesCS4A02G153600	1	3.1E+08	309514772	50S ribosomal protein L2
TraesCS4A02G153700	1	3.1E+08	309515445	Protein kinase superfamily protein
TraesCS4A02G153800	1	3.1E+08	309515780	Undecaprenyl-phosphate 4-deoxy-4-formamido-L-arabinose transferase
TraesCS4A02G153900	1	3.1E+08	309516511	S-adenosyl-L-methionine-dependent methyltransferases superfamily protein
TraesCS4A02G154000	1	3.1E+08	310241368	H/ACA ribonucleoprotein complex subunit 1-like protein 1
TraesCS4A02G154100	1	3.1E+08	310245711	Pumilio

GeneID	Gene2	gene_start_query	gene_end_query	Description
TraesCS4A02G154200	2	3.11E+08	310729855	Photosystem II reaction center protein I
TraesCS4A02G154300	2	3.11E+08	310730441	Photosystem II reaction center protein K
TraesCS4A02G154400	5	3.11E+08	310735011	Maturase K
TraesCS4A02G154500	1	3.11E+08	310737447	30S ribosomal protein S19, chloroplastic
TraesCS4A02G154600	1	3.11E+08	310738242	UDP-N-acetylglucosamine 1-carboxyvinyltransferase
TraesCS4A02G154700	1	3.11E+08	310739225	50S ribosomal protein L2
TraesCS4A02G154800	1	3.11E+08	310739247	Ribosomal protein L2
TraesCS4A02G154900	4	3.11E+08	310739978	Protein Ycf2
TraesCS4A02G155000	1	3.11E+08	310742452	4-hydroxy-2-oxovalerate aldolase 2
TraesCS4A02G155100	4	3.11E+08	310748961	Ycf68
TraesCS4A02G155200	1	3.11E+08	310750605	translocator assembly/maintenance protein
TraesCS4A02G155300	1	3.11E+08	310756216	6,7-dimethyl-8-ribityllumazine synthase
TraesCS4A02G155400	1	3.11E+08	310762503	NADH-quinone oxidoreductase subunit H 1
TraesCS4A02G155500	1	3.11E+08	310763661	NAD(P)H-quinone oxidoreductase subunit 6, chloroplastic
TraesCS4A02G155600	1	3.11E+08	310766849	NAD(P)H-quinone oxidoreductase chain 4
TraesCS4A02G155700	3	3.11E+08	310768165	Cytochrome c biogenesis protein CcsA
TraesCS4A02G155800	1	3.11E+08	310776835	30S ribosomal protein S15
TraesCS4A02G155900	1	3.11E+08	310782832	Sec14p-like phosphatidylinositol transfer family protein
TraesCS4A02G156000	4	3.11E+08	310784484	2-keto-3-deoxy-L-rhamnonate aldolase
TraesCS4A02G156100	1	3.11E+08	310789914	30S ribosomal protein S7
TraesCS4A02G156200	1	3.11E+08	310791575	NAD(P)H-quinone oxidoreductase subunit 2, chloroplastic
TraesCS4A02G156300	1	3.12E+08	311664820	ABC transporter ATP-binding protein
TraesCS4A02G156500	1	3.13E+08	312708877	Vacuolar cation/proton exchanger, putative
TraesCS4A02G156600	1	3.13E+08	312883445	RING zinc finger-containing protein
TraesCS4A02G156700	1	3.14E+08	313639691	Villin
TraesCS4A02G156800	1	3.14E+08	313752415	Anaphase spindle elongation protein 1
TraesCS4A02G156900	1	3.15E+08	314697897	Chromophore lyase CpcT/CpeT
TraesCS4A02G157000	1	3.17E+08	317090756	SAP domain-containing protein
TraesCS4A02G157100	1	3.18E+08	318045161	RNA polymerase sigma factor
TraesCS4A02G157200	1	3.2E+08	319844862	GTPase obg

GeneID	Gene2	gene_start_query	gene_end_query	Description
TraesCS4A02G157300	1	3.2E+08	320273940	RNA-binding protein
TraesCS4A02G157400	1	3.21E+08	321184345	Cellulose synthase family protein
TraesCS4A02G157500	1	3.21E+08	321195641	Ubiquitin-like-specific protease ESD4
TraesCS4A02G157600	1	3.22E+08	321905801	Transmembrane protein 184C
TraesCS4A02G157700	1	3.23E+08	323051613	splicing factor-like protein
TraesCS4A02G157800	1	3.24E+08	323785847	PQ-loop repeat family protein / transmembrane family protein
TraesCS4A02G157900	1	3.24E+08	323789209	Oxidoreductase/ transition metal ion binding protein
TraesCS4A02G158000	1	3.24E+08	323854300	Peter Pan-like protein
TraesCS4A02G158100	1	3.25E+08	324708729	Auxin-repressed/dormancy-associated protein
TraesCS4A02G158200	1	3.25E+08	325196452	ATP-dependent RNA helicase, putative
TraesCS4A02G158300	1	3.26E+08	326137945	TRAF type zinc finger domain containing 1
TraesCS4A02G158400	1	3.27E+08	327261042	tRNA-2-methylthio-N(6)-dimethylallyladenosine synthase
TraesCS4A02G158600	1	3.28E+08	327747380	B3 domain-containing protein
TraesCS4A02G158700	1	3.28E+08	327757720	Sister-chromatid cohesion protein 3
TraesCS4A02G158800	1	3.29E+08	329172009	GRF zinc finger family protein, expressed
TraesCS4A02G158900	1	3.29E+08	329188700	ATP synthase epsilon chain
TraesCS4A02G159000	1	3.3E+08	330103702	myosin-binding protein (Protein of unknown function, DUF593)
TraesCS4A02G159100	1	3.32E+08	331632278	Thiol:disulfide interchange protein TxlA
TraesCS4A02G159200	1	3.33E+08	332659130	Pimeloyl-[acyl-carrier protein] methyl ester esterase
TraesCS4A02G159300	1	3.34E+08	334268654	T-complex protein 1 subunit zeta
TraesCS4A02G159400	1	3.39E+08	338749134	Agenet domain containing protein
TraesCS4A02G159500	1	3.4E+08	340508791	Transmembrane protein 87A
TraesCS4A02G159600	1	3.41E+08	340511300	Isopentenyl-diphosphate delta-isomerase
TraesCS4A02G159700	1	3.42E+08	341782585	Cystathionine gamma-synthase
TraesCS4A02G159800	1	3.42E+08	341994093	myo-inositol oxygenase 5
TraesCS4A02G159900	1	3.42E+08	342464186	O-fucosyltransferase family protein
TraesCS4A02G160000	1	3.44E+08	343803198	Tubulin alpha-6 chain
TraesCS4A02G160100	1	3.44E+08	343908352	Plant calmodulin-binding protein-like protein
TraesCS4A02G160200	1	3.44E+08	343937295	Short chain dehydrogenase/reductase family 42E member 1
TraesCS4A02G160300	1	3.44E+08	343936672	tRNA pseudouridine synthase A

GeneID	Gene2	gene_start_query	gene_end_query	Description
TraesCS4A02G160400	1	3.44E+08	344035918	Basic leucine zipper and W2 domain-containing protein 2
TraesCS4A02G160500	1	3.45E+08	344964824	Heavy metal transport/detoxification superfamily protein
TraesCS4A02G160600	1	3.46E+08	345844921	Phosphatidate phosphatase, Lipin
TraesCS4A02G160700	1	3.46E+08	346064702	Transducin/WD-like repeat-protein
TraesCS4A02G160800	1	3.47E+08	346688223	2,3-bisphosphoglycerate-dependent phosphoglycerate mutase
TraesCS4A02G160900	1	3.47E+08	347402111	Chaperone protein DnaJ
TraesCS4A02G161000	1	3.49E+08	348504742	Acetyl-coenzyme A carboxylase carboxyl transferase subunit beta, chloroplastic
TraesCS4A02G161100	1	3.49E+08	349310580	RING/U-box superfamily protein
TraesCS4A02G161200	1	3.5E+08	349887485	Acetyl-coenzyme A carboxylase carboxyl transferase subunit beta, chloroplastic
TraesCS4A02G161300	1	3.5E+08	350173482	ATP synthase subunit 9, mitochondrial
TraesCS4A02G161400	1	3.5E+08	350238804	XH/XS domain-containing protein
TraesCS4A02G161500	1	3.5E+08	350239272	RmlC-like cupins superfamily protein
TraesCS4A02G161600	2	3.5E+08	350244759	Cytochrome b559 subunit alpha
TraesCS4A02G161700	1	3.52E+08	351899145	Speckle-type POZ protein-like protein B
TraesCS4A02G161900	1	3.52E+08	352276765	S-acyltransferase
TraesCS4A02G162000	1	3.52E+08	352513698	E3 ubiquitin-protein ligase
TraesCS4A02G162100	1	3.53E+08	353091693	Stearoyl-[acyl-carrier-protein] 9-desaturase, chloroplastic
TraesCS4A02G162200	1	3.54E+08	353877943	Dentin sialophosphoprotein-related, putative isoform 1
TraesCS4A02G162300	1	3.57E+08	356556917	SNARE
TraesCS4A02G162400	1	3.57E+08	357262996	ATP-dependent RNA helicase
TraesCS4A02G162500	1	3.58E+08	357694116	WRKY transcription factor
TraesCS4A02G162600	1	3.59E+08	358840615	DNA-directed RNA polymerase subunit beta
TraesCS4A02G162700	1	3.59E+08	358873662	1-phosphatidylinositol-3-phosphate 5-kinase
TraesCS4A02G162800	1	3.6E+08	359640296	Ubiquitin system component Cue protein
TraesCS4A02G163000	1	3.62E+08	362113814	Ubiquitin carboxyl-terminal hydrolase family protein, expressed
TraesCS4A02G163100	1	3.63E+08	363122351	Transcription initiation factor TFIID subunit 4b
TraesCS4A02G163200	1	3.64E+08	363713421	S-adenosyl-L-methionine-dependent methyltransferases superfamily protein
TraesCS4A02G163300	1	3.64E+08	363748257	Transcription factor
TraesCS4A02G163400	1	3.66E+08	365658289	Rho GTPase-activating protein
TraesCS4A02G163500	1	3.69E+08	368571931	Mannose-6-phosphate isomerase

GeneID	Gene2	gene_start_query	gene_end_query	Description
TraesCS4A02G163600	1	3.7E+08	369543816	WD repeat-containing protein 1
TraesCS4A02G163700	1	3.72E+08	371796858	Inositol-1-monophosphatase family protein
TraesCS4A02G163800	1	3.74E+08	373809076	C2 calcium/lipid-binding and GRAM domain containing protein
TraesCS4A02G163900	1	3.75E+08	374659814	ATP-binding cassette sub-family G member 5
TraesCS4A02G164000	1	3.76E+08	375733434	Flavin-binding kelch domain F box protein
TraesCS4A02G164100	1	3.77E+08	376728979	RING/FYVE/PHD zinc finger superfamily protein
TraesCS4A02G164200	1	3.78E+08	377596428	GRF zinc finger family protein
TraesCS4A02G164300	1	3.79E+08	379045201	Glycosyltransferase
TraesCS4A02G164400	1	3.79E+08	379469407	3-ketoacyl-CoA synthase
TraesCS4A02G164500	1	3.8E+08	380148176	phosphoglycolate phosphatase
TraesCS4A02G164600	1	3.81E+08	381228055	Vacuolar protein sorting-associated
TraesCS4A02G164700	1	3.83E+08	383180995	Formin-like protein
TraesCS4A02G164800	1	3.84E+08	383547554	Protein SUPPRESSOR OF GENE SILENCING 3
TraesCS4A02G164900	1	3.84E+08	384398821	T-complex protein 1 subunit theta
TraesCS4A02G165000	1	3.86E+08	385774186	Splicing factor u2af large subunit, putative
TraesCS4A02G165100	1	3.86E+08	385781526	Adenylyl-sulfate kinase
TraesCS4A02G165200	1	3.87E+08	387130365	ATP-dependent RNA helicase
TraesCS4A02G165300	1	3.89E+08	389251601	E3 ubiquitin-protein ligase family
TraesCS4A02G165400	1	3.9E+08	389991790	Phosphatidylinositol-4-phosphate 5-kinase 4, putative, expressed
TraesCS4A02G165500	1	3.91E+08	390911142	Protein ENHANCED DISEASE RESISTANCE 2-like
TraesCS4A02G165600	1	3.95E+08	395034817	3-oxoacyl-[acyl-carrier-protein] synthase
TraesCS4A02G165700	1	3.97E+08	396754973	stearoyl-[acyl-carrier-protein] 9-desaturase, chloroplastic
TraesCS4A02G165800	1	3.97E+08	396757269	ADP-L-glycero-D-manno-heptose-6-epimerase
TraesCS4A02G165900	1	3.98E+08	397611165	Carnitinyl-CoA dehydratase
TraesCS4A02G166000	1	3.98E+08	398499003	DNA topoisomerase
TraesCS4A02G166100	1	3.99E+08	398506190	Pathogenesis-related thaumatin-like protein
TraesCS4A02G166200	1	4.01E+08	400760929	Beta-adaptin-like protein
TraesCS4A02G166300	1	4.01E+08	400851800	DNA helicase ROCK-N-ROLLERS
TraesCS4A02G166400	1	4.01E+08	401303756	Transient receptor potential cation channel subfamily V member 4
TraesCS4A02G166500	1	4.03E+08	403387985	Eukaryotic translation initiation factor 3 subunit I

GeneID	Gene2	gene_start_query	gene_end_query	Description
TraesCS4A02G166600	1	4.06E+08	405579589	Zinc finger, PHD-finger
TraesCS4A02G166700	1	4.07E+08	407465049	Senescence-associated protein
TraesCS4A02G166800	1	4.08E+08	407611328	Clustered mitochondria protein homolog
TraesCS4A02G166900	1	4.09E+08	409037882	Alpha-1,4-glucan-protein synthase [UDP-forming] 1
TraesCS4A02G167000	1	4.1E+08	410395633	30S ribosomal protein S5

Table S5. Population pairwise differentiation (PhiPT) of cultivated wheat types and wild relatives.

Pop	TaesClu	TaesCul	TaesInd	TaesLan	TaesMac	TaesSpe	TaesTib	TaesYun	TispIsp	TkarGeo	TmonDom	TmonWil	TpetXin	TturDom	TturDur	TturKho	TturPer	TturPol	TturRiv	TturWil	TuraUra	TvavVav
**TaesClu**		0.004	0.135	0.215	0.119	0.010	0.358	0.165	0.149	0.267	0.000	0.000	0.284	0.012	0.019	0.084	0.088	0.080	0.066	0.014	0.000	0.176
**TaesCul**	0.413		0.170	0.000	0.000	0.000	0.163	0.021	0.217	0.108	0.000	0.000	0.136	0.420	0.348	0.287	0.270	0.288	0.326	0.439	0.000	0.161
**TaesInd**	0.208	0.000		0.120	0.007	0.000	0.174	0.353	0.447	0.304	0.000	0.000	0.434	0.493	0.387	0.489	0.478	0.493	0.481	0.466	0.000	0.499
**TaesLan**	0.000	0.206	0.160		0.008	0.000	0.127	0.231	0.038	0.265	0.000	0.000	0.221	0.000	0.006	0.015	0.022	0.011	0.007	0.000	0.000	0.132
**TaesMac**	0.353	0.883	0.849	0.335		0.438	0.008	0.035	0.002	0.018	0.000	0.000	0.002	0.000	0.000	0.000	0.001	0.000	0.001	0.000	0.000	0.008
**TaesSpe**	0.390	0.826	0.763	0.344	0.000		0.000	0.001	0.000	0.003	0.000	0.000	0.001	0.000	0.000	0.000	0.000	0.000	0.000	0.000	0.000	0.000
**TaesTib**	0.191	0.000	0.004	0.156	0.835	0.755		0.511	0.526	0.553	0.000	0.000	0.482	0.423	0.033	0.471	0.493	0.470	0.483	0.473	0.000	0.453
**TaesYun**	0.000	0.127	0.003	0.000	0.597	0.591	0.000		0.412	0.374	0.000	0.000	0.428	0.130	0.096	0.328	0.344	0.313	0.288	0.144	0.000	0.475
**TispIsp**	0.240	0.010	0.000	0.173	0.841	0.765	0.000	0.030		0.606	0.000	0.000	0.482	0.005	0.000	0.065	0.006	0.051	0.047	0.005	0.000	0.463
**TkarGeo**	0.089	0.000	0.015	0.110	0.815	0.738	0.000	0.000	0.000		0.000	0.001	0.397	0.503	0.279	0.549	0.533	0.541	0.546	0.519	0.000	0.528
**TmonDom**	0.494	0.180	0.227	0.239	0.908	0.844	0.233	0.267	0.229	0.228		0.000	0.000	0.000	0.000	0.000	0.000	0.000	0.000	0.000	0.000	0.000
**TmonWil**	0.508	0.169	0.209	0.243	0.909	0.849	0.218	0.273	0.217	0.208	0.028		0.000	0.000	0.000	0.000	0.000	0.000	0.000	0.000	0.000	0.000
**TpetXin**	0.154	0.000	0.000	0.140	0.831	0.750	0.000	0.000	0.000	0.005	0.231	0.212		0.495	0.226	0.492	0.496	0.501	0.494	0.490	0.000	0.224
**TturDom**	0.487	0.000	0.000	0.238	0.902	0.847	0.003	0.203	0.022	0.000	0.208	0.197	0.000		0.447	0.067	0.481	0.259	0.030	0.041	0.000	0.495
**TturDur**	0.398	0.000	0.005	0.211	0.898	0.816	0.038	0.154	0.038	0.035	0.239	0.221	0.023	0.000		0.002	0.014	0.005	0.001	0.470	0.000	0.182
**TturKho**	0.307	0.000	0.000	0.191	0.861	0.785	0.000	0.076	0.005	0.000	0.216	0.203	0.000	0.007	0.014		0.496	0.493	0.051	0.067	0.000	0.462
**TturPer**	0.298	0.000	0.000	0.188	0.863	0.783	0.000	0.071	0.008	0.000	0.222	0.207	0.000	0.000	0.013	0.000		0.491	0.493	0.478	0.000	0.487
**TturPol**	0.315	0.000	0.000	0.193	0.867	0.789	0.000	0.085	0.006	0.000	0.218	0.204	0.000	0.003	0.011	0.000	0.000		0.347	0.330	0.000	0.488
**TturRiv**	0.338	0.000	0.000	0.199	0.868	0.795	0.000	0.097	0.006	0.000	0.211	0.200	0.000	0.007	0.009	0.003	0.000	0.001		0.044	0.000	0.466
**TturWil**	0.477	0.000	0.000	0.235	0.899	0.844	0.000	0.194	0.019	0.000	0.205	0.192	0.000	0.001	0.000	0.006	0.000	0.001	0.005		0.000	0.470
**TuraUra**	0.531	0.251	0.336	0.247	0.919	0.854	0.337	0.323	0.322	0.350	0.241	0.225	0.342	0.285	0.335	0.306	0.315	0.312	0.297	0.280		0.000
**TvavVav**	0.201	0.000	0.000	0.158	0.844	0.760	0.000	0.000	0.000	0.001	0.232	0.212	0.005	0.000	0.018	0.000	0.000	0.000	0.000	0.000	0.337	

Below diagonal are PhiPT values and above diagonal values indicate Pvalues.

Species: Taes=*T.aestivum*, Tisp=*T. ispahanicum*, Tkar=*T. karamyschevii*, Tmon=*T.monococcum*, Tpet=*T. petropavlovskyi*,Ttur=*T. turgidum*, Tura=*T.urartu*,Tvav=*T.vavilovii*

Wheat types: Clu=Club, Cul=caltivars, Dom=Domesticated, Dur=Durum, Geo=Georgian, Ind=Indian dwarf, isp= Ispahanicum, Kho= Khorasan, Lan=Landraces(bread wheat),Mac=Macha, Per=Persian, Pol=Polish, Riv=Rivet, Spe=Spelt, Tib=Tibetan Semi-wild, Ura=Urartu, Vav=Vavilovii, Wil-Wild, Xin= Xinjiang, Yun=Yunan

Table S6. Population pairwise differentiation based on maximum likelihood statistical significance (G-test) of cultivated wheat types and wild relatives.

Pop	TaesClu	TaesCul	TaesInd	TaesLan	TaesMac	TaesSpe	TaesTib	TaesYun	TispIsp	TkarGeo	TmonDom	TmonWil	TpetXin	TturDom	TturDur	TturKho	TturPer	TturPol	TturRiv	TturWil	TuraUra	TvavVav
**TaesClu**		***	***	1	***	***	***	1		***	***	***	***	***	***	***	***	***	***	***	***	***
**TaesCul**	50227		1	***	***	***	1	***	1	1	1	***	1	***	1		1	1	1	***	***	1.0
**TaesInd**	23509	103		***	***	***	1	1	1	0.4	***	***	1	1	1	1	0.9	0.8	1	1	***	1.0
**TaesLan**	419	13089	47175		***	***	***	***	***	***	***	***	***	***	***	***	***	***	***	***	***	***
**TaesMac**	41577	131036	108836	79278		1	***	***	***	***	***	***	***	***	***	***	***	***	***	***	***	***
**TaesSpe**	80028	131224	130935	130786	3159		***	***	***	***	***	***	***	***	***	***	***	***	***	***	***	***
**TaesTib**	23484	103	0	47145	108781	130897		1	1	1	***	***	1	1	0.9	1	0.8	0.7	1	1	***	1
**TaesYun**	4599	19429	7127	12182	67211	117426	7115		***	1	***	***	1	***	***	***	***	***	***	***	***	1
**TispIsp**	37879	4223	3	65187	126651	131177	17	17540		1	***	***	1	1	0.7	1	0.9	0.7	1	1	***	1
**TkarGeo**	20096	14	6	28568	83738	120118	6	6590	9		***	***	0.3	1	1	1	0.6	0.5	1	1	***	0.5
**TmonDom**	74509	9440	2422	131187	128837	128915	2450	36691	2515	2415		***	***	***	***	***	***	***	***	***	***	***
**TmonWil**	78057	16615	2245	131207	129038	129088	2275	38247	2386	2242	488		***	***	***	***	***	***	***	***	***	***
**TpetXin**	22945	88	0	36496	99602	129439	0	7038	1	5	2418	2239		1	1	1	1	0.9	1	1	***	0.8
**TturDom**	80063	15558	4	131221	131210	131224	35	38026	92	15	2518	2524	0		1		1	1	1	1	***	1.0
**TturDur**	54589	6195	10	121333	130965	131214	27	25610	68	10	2479	2375	7	61		0.7	0.8	0.8	0.6	1	***	0.9
**TturKho**	47108	5411	10	92375	130278	131207	27	21972	56	15	2496	2375	7	89	53		1	1	1	1	***	1.0
**TturPer**	44338	5031	6	83393	129642	131204	23	20656	51	12	2457	2333	4	40	24	21		0	1	1	***	0.7
**TturPol**	47161	5427	8	92420	130216	131208	22	22019	57	13	2464	2345	5	45	22	16	20		1	1	***	0.6
**TturRiv**	52173	5962	17.0	111852	130873	131213	37	24420	73	10	2520	2407	13	99	63	55	32	35		0.9	***	1.0
**TturWil**	78805	15146	4.7	131215	131215	131224	35	37416	88	14	2535	2527	1	135	74	98	51	57	121		***	1.0
**TuraUra**	77480	17307	3345	131198	127962	128062	3374	38608	3447	3350	2488	2437	3340	3472	3415	3432	3388	3397	3450	3488		***
**TvavVav**	23500	107	2	47168	109004	130910	2	7123	7	8	2422	2251	2	8	15	14	10	13	17	13	3349	

Below diagonal are Chi square values and above diagonal values indicate significance, ***indicates strongly significant, and other numeric values indicate Pvalues.

Species: Taes=*T.aestivum*, Tisp=*T. ispahanicum*, Tkar=*T. karamyschevii*, Tmon=*T.monococcum*, Tpet=*T. petropavlovskyi*,Ttur=*T. turgidum*, Tura=*T.urartu*,Tvav=*T.vavilovii*

Wheat types: Clu=Club, Cul=caltivars, Dom=Domesticated, Dur=Durum, Geo=Georgian, Ind=Indian dwarf, isp= Ispahanicum, Kho= Khorasan, Lan=Landraces(bread wheat),Mac=Macha, Per=Persian, Pol=Polish, Riv=Rivet, Spe=Spelt, Tib=Tibetan Semi-wild, Ura=Ura, Vav=Vavilovii, Wil-Wild, Xin= Xinjiang, Yun=Yunan

Table S7. Ancestral coefficients (Q-matrix).

Accession	Q1	Q2	Q3	Q4	Q5
Taes_Clu_TW001	0.995507	0.004193	1.00E-04	1.00E-04	1.00E-04
Taes_Clu_TW002	0.973649	1.00E-04	1.00E-04	1.00E-04	0.026052
Taes_Clu_TW003	1.00E-04	1.00E-04	0.956833	0.042867	1.00E-04
Taes_Clu_TW004	1.00E-04	0.001501	0.960286	0.038013	1.00E-04
Taes_Clu_TW005	0.983518	0.013907	1.00E-04	1.00E-04	0.002375
Taes_Cul_AMN	0.961704	0.037996	1.00E-04	1.00E-04	1.00E-04
Taes_Cul_BJBH	0.995767	1.00E-04	1.00E-04	1.00E-04	0.003933
Taes_Cul_CAN-C1	0.001622	0.0015	0.996679	1.00E-04	1.00E-04
Taes_Cul_CHL-C1	0.995306	0.004394	1.00E-04	1.00E-04	1.00E-04
Taes_Cul_CZ6406	0.998084	0.001616	1.00E-04	1.00E-04	1.00E-04
Taes_Cul_FMSY	0.997238	0.002462	1.00E-04	1.00E-04	1.00E-04
Taes_Cul_FRA-C1	0.996272	0.003429	1.00E-04	1.00E-04	1.00E-04
Taes_Cul_GYWLL	0.97556	0.021722	1.00E-04	1.00E-04	0.002518
Taes_Cul_HNSH	0.996235	1.00E-04	1.00E-04	0.003465	1.00E-04
Taes_Cul_ITA-C1	0.982413	0.005597	1.00E-04	1.00E-04	0.011791
Taes_Cul_JCYLYL	0.9996	1.00E-04	1.00E-04	1.00E-04	1.00E-04
Taes_Cul_JM8H	0.960817	0.029932	1.00E-04	1.00E-04	0.009051
Taes_Cul_KFEH	0.969403	0.024458	1.00E-04	1.00E-04	0.005939
Taes_Cul_KFSH	0.971964	0.025234	1.00E-04	1.00E-04	0.002602
Taes_Cul_KXSH	0.983018	0.014856	1.00E-04	1.00E-04	0.001926
Taes_Cul_NLD-C1	0.981779	0.006296	1.00E-04	1.00E-04	0.011725
Taes_Cul_NXWH	0.9996	1.00E-04	1.00E-04	1.00E-04	1.00E-04
Taes_Cul_PYEQ	0.984559	0.001606	0.00124	1.00E-04	0.012495
Taes_Cul_USA-C1	0.997843	1.00E-04	1.00E-04	1.00E-04	0.001857
Taes_Cul_UZB-C1	0.9996	1.00E-04	1.00E-04	1.00E-04	1.00E-04
Taes_Cul_XYLH	0.9996	1.00E-04	1.00E-04	1.00E-04	1.00E-04
Taes_Cul_YASY	0.972921	0.025002	1.00E-04	1.00E-04	0.001877
Taes_Cul_YNSW	0.986309	0.012442	1.00E-04	1.00E-04	0.00105
Taes_Cul_ZDSH	0.998705	0.000995	1.00E-04	1.00E-04	1.00E-04
Taes_Cul_ZYJWLQ	0.984319	1.00E-04	1.00E-04	1.00E-04	0.015381
Taes_Ind_TW025	0.989669	1.00E-04	1.00E-04	1.00E-04	0.010031
Taes_Ind_TW026	0.940115	0.030157	1.00E-04	1.00E-04	0.029528
Taes_Ind_TW027	0.972526	0.00361	1.00E-04	1.00E-04	0.023664
Taes_Ind_TW028	0.981167	0.016113	1.00E-04	1.00E-04	0.00252
Taes_Ind_TW029	0.982635	0.014706	1.00E-04	1.00E-04	0.002459
Taes_Lan_AFG-L1	0.997366	0.002334	1.00E-04	1.00E-04	1.00E-04
Taes_Lan_AFG-L2	0.991988	1.00E-04	1.00E-04	1.00E-04	0.007712
Taes_Lan_AFG-L3	1.00E-04	1.00E-04	0.9996	1.00E-04	1.00E-04
Taes_Lan_AUT-L1	0.001063	1.00E-04	0.998637	1.00E-04	1.00E-04
Taes_Lan_BDHHM	0.986652	1.00E-04	1.00E-04	1.00E-04	0.013049
Taes_Lan_BIH-L1	1.00E-04	1.00E-04	0.465945	0.533755	1.00E-04
Taes_Lan_BMZ	0.980366	0.019334	1.00E-04	1.00E-04	1.00E-04

Accession	Q1	Q2	Q3	Q4	Q5
Taes_Lan_BQM	1.00E-04	1.00E-04	0.9996	1.00E-04	1.00E-04
Taes_Lan_BRA-L1	0.961788	0.037912	1.00E-04	1.00E-04	1.00E-04
Taes_Lan_BYM	0.378873	1.00E-04	1.00E-04	0.620827	1.00E-04
Taes_Lan_CHE-L1	0.000393	1.00E-04	0.999307	1.00E-04	1.00E-04
Taes_Lan_CSB	0.983022	0.004137	1.00E-04	1.00E-04	0.012641
Taes_Lan_CYHM	1.00E-04	1.00E-04	0.995118	1.00E-04	0.004583
Taes_Lan_DEU-L1	1.00E-04	0.00266	0.99704	1.00E-04	1.00E-04
Taes_Lan_DHM	0.960394	0.039306	1.00E-04	1.00E-04	1.00E-04
Taes_Lan_DKM	0.971709	0.025614	1.00E-04	1.00E-04	0.002476
Taes_Lan_DXHKYM	0.987591	0.005095	1.00E-04	1.00E-04	0.007114
Taes_Lan_DYH	0.980991	0.003794	1.00E-04	1.00E-04	0.015015
Taes_Lan_ESP-L1	1.00E-04	1.00E-04	0.9996	1.00E-04	1.00E-04
Taes_Lan_FIN-L1	1.00E-04	1.00E-04	0.998808	1.00E-04	0.000892
Taes_Lan_GBR-L1	0.987106	1.00E-04	1.00E-04	1.00E-04	0.012594
Taes_Lan_GEO-L1	1.00E-04	1.00E-04	0.9996	1.00E-04	1.00E-04
Taes_Lan_GRC-L1	1.00E-04	1.00E-04	0.9996	1.00E-04	1.00E-04
Taes_Lan_HCM	0.9996	1.00E-04	1.00E-04	1.00E-04	1.00E-04
Taes_Lan_HLM	0.9996	1.00E-04	1.00E-04	1.00E-04	1.00E-04
Taes_Lan_HM	0.930007	0.060861	1.00E-04	1.00E-04	0.008933
Taes_Lan_HMM	0.978662	0.007496	1.00E-04	1.00E-04	0.013641
Taes_Lan_HRV-L1	0.964184	0.001545	1.00E-04	1.00E-04	0.034071
Taes_Lan_HTZ	0.9996	1.00E-04	1.00E-04	1.00E-04	1.00E-04
Taes_Lan_HUN-L1	0.990634	1.00E-04	1.00E-04	1.00E-04	0.009066
Taes_Lan_HZB	0.977796	0.007459	1.00E-04	1.00E-04	0.014546
Taes_Lan_IND-L1	0.991753	1.00E-04	1.00E-04	1.00E-04	0.007947
Taes_Lan_IND-L2	0.96682	0.003952	1.00E-04	1.00E-04	0.029027
Taes_Lan_IRN-L1	1.00E-04	1.00E-04	0.9996	1.00E-04	1.00E-04
Taes_Lan_IRN-L2	1.00E-04	1.00E-04	0.9996	1.00E-04	1.00E-04
Taes_Lan_IRN-L3	1.00E-04	1.00E-04	0.9996	1.00E-04	1.00E-04
Taes_Lan_IRN-L4	0.005544	1.00E-04	0.994156	1.00E-04	1.00E-04
Taes_Lan_IRQ-L1	0.975451	1.00E-04	1.00E-04	1.00E-04	0.024249
Taes_Lan_IRQ-L2	1.00E-04	1.00E-04	0.99739	1.00E-04	0.00231
Taes_Lan_IRQ-L3	1.00E-04	1.00E-04	0.9996	1.00E-04	1.00E-04
Taes_Lan_IRQ-L4	1.00E-04	1.00E-04	0.9996	1.00E-04	1.00E-04
Taes_Lan_ITA-L1	0.992479	1.00E-04	0.000386	1.00E-04	0.006935
Taes_Lan_ITA-L2	0.991334	0.008366	1.00E-04	1.00E-04	1.00E-04
Taes_Lan_JiaHM	1.00E-04	1.00E-04	0.106521	0.893179	1.00E-04
Taes_Lan_JinHM	0.951961	0.01582	1.00E-04	1.00E-04	0.032019
Taes_Lan_JM	1.00E-04	1.00E-04	0.9996	1.00E-04	1.00E-04
Taes_Lan_JPN-L1	1.00E-04	1.00E-04	0.998121	1.00E-04	0.001579
Taes_Lan_KAZ-L1	0.99634	0.003098	0.000361	1.00E-04	1.00E-04
Taes_Lan_KSBP	1.00E-04	1.00E-04	0.989924	0.009776	1.00E-04
Taes_Lan_LGD	1.00E-04	1.00E-04	0.690759	0.308941	1.00E-04

Accession	Q1	Q2	Q3	Q4	Q5
Taes_Lan_LLX	0.990916	1.00E-04	1.00E-04	1.00E-04	0.008784
Taes_Lan_LM	1.00E-04	1.00E-04	1.00E-04	0.9996	1.00E-04
Taes_Lan_LTT	0.000154	1.00E-04	0.999546	1.00E-04	1.00E-04
Taes_Lan_LXZXM	0.982905	0.013349	1.00E-04	1.00E-04	0.003545
Taes_Lan_LZT	0.995772	0.003766	1.00E-04	1.00E-04	0.000263
Taes_Lan_MEX-L1	1.00E-04	1.00E-04	0.9996	1.00E-04	1.00E-04
Taes_Lan_MKD-L1	1.00E-04	1.00E-04	1.00E-04	0.9996	1.00E-04
Taes_Lan_MXM	1.00E-04	1.00E-04	0.9996	1.00E-04	1.00E-04
Taes_Lan_NLD-L1	0.9996	1.00E-04	1.00E-04	1.00E-04	1.00E-04
Taes_Lan_PAK-L1	0.990516	1.00E-04	1.00E-04	1.00E-04	0.009184
Taes_Lan_PAK-L2	0.972799	0.026901	1.00E-04	1.00E-04	1.00E-04
Taes_Lan_PAK-L3	0.9996	1.00E-04	1.00E-04	1.00E-04	1.00E-04
Taes_Lan_PRT-L1	1.00E-04	1.00E-04	0.9996	1.00E-04	1.00E-04
Taes_Lan_QCM	0.985372	0.009439	1.00E-04	1.00E-04	0.00499
Taes_Lan_ROM-L1	0.973659	1.00E-04	1.00E-04	1.00E-04	0.026041
Taes_Lan_RUS-L1	1.00E-04	1.00E-04	0.9996	1.00E-04	1.00E-04
Taes_Lan_SLZ	0.995083	0.004617	1.00E-04	1.00E-04	1.00E-04
Taes_Lan_SM	0.979389	0.005992	1.00E-04	1.00E-04	0.014419
Taes_Lan_SRB-L1	0.984091	1.00E-04	1.00E-04	1.00E-04	0.015609
Taes_Lan_SRM4H	0.996587	0.000908	1.00E-04	0.002305	1.00E-04
Taes_Lan_SWE-L1	0.990946	0.008754	1.00E-04	1.00E-04	1.00E-04
Taes_Lan_SYR-L1	1.00E-04	1.00E-04	0.9996	1.00E-04	1.00E-04
Taes_Lan_SYR-L2	1.00E-04	1.00E-04	0.997374	1.00E-04	0.002326
Taes_Lan_TJBXM	0.982435	0.009465	1.00E-04	0.002957	0.005043
Taes_Lan_TJK-L1	0.969933	0.009223	1.00E-04	1.00E-04	0.020645
Taes_Lan_TMM	0.979281	0.020419	1.00E-04	1.00E-04	1.00E-04
Taes_Lan_TUR-L1	1.00E-04	1.00E-04	0.9996	1.00E-04	1.00E-04
Taes_Lan_TUR-L2	1.00E-04	1.00E-04	0.998996	1.00E-04	0.000704
Taes_Lan_TUR-L3	1.00E-04	1.00E-04	0.9996	1.00E-04	1.00E-04
Taes_Lan_TUR-L4	1.00E-04	0.000681	0.99902	1.00E-04	1.00E-04
Taes_Lan_TW050	0.981832	0.006528	1.00E-04	1.00E-04	0.011439
Taes_Lan_TW051	0.983443	1.00E-04	1.00E-04	1.00E-04	0.016257
Taes_Lan_TW052	1.00E-04	0.0004	0.961332	0.038068	1.00E-04
Taes_Lan_TW053	0.981877	0.003198	1.00E-04	1.00E-04	0.014725
Taes_Lan_TW054	0.982704	0.016997	1.00E-04	1.00E-04	1.00E-04
Taes_Lan_UKR-L1	0.000605	1.00E-04	0.999095	1.00E-04	1.00E-04
Taes_Lan_UKR-L2	0.993873	0.005828	1.00E-04	1.00E-04	1.00E-04
Taes_Lan_URY-L1	0.947541	0.052159	1.00E-04	1.00E-04	1.00E-04
Taes_Lan_URY-L2	0.9996	1.00E-04	1.00E-04	1.00E-04	1.00E-04
Taes_Lan_UZB-L1	0.9996	1.00E-04	1.00E-04	1.00E-04	1.00E-04
Taes_Lan_WYM	0.971474	0.020956	1.00E-04	1.00E-04	0.00737
Taes_Lan_XFS	0.973656	1.00E-04	1.00E-04	1.00E-04	0.026045
Taes_Lan_XHP	0.9996	1.00E-04	1.00E-04	1.00E-04	1.00E-04

Accession	Q1	Q2	Q3	Q4	Q5
Taes_Lan_YM	1.00E-04	1.00E-04	0.9996	1.00E-04	1.00E-04
Taes_Lan_YMBF	0.995422	0.004278	1.00E-04	1.00E-04	1.00E-04
Taes_Lan_YZM	0.953329	0.037941	1.00E-04	1.00E-04	0.008531
Taes_Lan_ZJH	0.992829	0.006871	1.00E-04	1.00E-04	1.00E-04
Taes_Lan_ZP	0.015176	1.00E-04	1.00E-04	0.984524	1.00E-04
Taes_Lan_ZWT	1.00E-04	1.00E-04	0.9996	1.00E-04	1.00E-04
Taes_Mac_TW006	1.00E-04	1.00E-04	0.939737	0.057041	0.003022
Taes_Mac_TW007	0.002576	1.00E-04	0.972987	0.024236	1.00E-04
Taes_Mac_TW008	1.00E-04	0.003631	0.921667	0.074502	1.00E-04
Taes_Mac_TW009	1.00E-04	1.00E-04	0.971812	0.027888	1.00E-04
Taes_Mac_TW010	1.00E-04	1.00E-04	0.938401	0.061299	1.00E-04
Taes_Spe_TW011	1.00E-04	0.004735	0.95929	0.035775	1.00E-04
Taes_Spe_TW012	1.00E-04	0.003795	0.877805	0.1182	1.00E-04
Taes_Spe_TW013	1.00E-04	1.00E-04	0.921988	0.069599	0.008213
Taes_Spe_TW014	1.00E-04	1.00E-04	0.950462	0.041114	0.008224
Taes_Spe_TW015	0.002344	1.00E-04	0.977109	0.020346	1.00E-04
Taes_Spe_TW016	0.000311	1.00E-04	0.972883	0.026606	1.00E-04
Taes_Spe_TW017	1.00E-04	1.00E-04	0.93189	0.065453	0.002457
Taes_Spe_TW018	0.004381	1.00E-04	0.977911	0.017508	1.00E-04
Taes_Spe_TW019	1.00E-04	1.00E-04	0.922641	0.074384	0.002775
Taes_Spe_TW020	1.00E-04	1.00E-04	0.946182	0.052116	0.001502
Taes_Spe_TW021	1.00E-04	1.00E-04	0.92605	0.07365	1.00E-04
Taes_Spe_TW022	1.00E-04	1.00E-04	0.906181	0.091956	0.001664
Taes_Spe_TW023	0.004107	1.00E-04	0.877798	0.117896	1.00E-04
Taes_Spe_TW024	1.00E-04	1.00E-04	0.973347	0.026353	1.00E-04
Taes_Tib_TW035	0.959673	0.040027	1.00E-04	1.00E-04	1.00E-04
Taes_Tib_TW036	0.92828	0.033415	1.00E-04	1.00E-04	0.038105
Taes_Tib_TW037	0.982345	0.017355	1.00E-04	1.00E-04	1.00E-04
Taes_Tib_TW038	0.966927	0.028519	1.00E-04	1.00E-04	0.004354
Taes_Tib_TW039	0.995202	0.002557	1.00E-04	1.00E-04	0.002042
Taes_Yun_TW030	0.9996	1.00E-04	1.00E-04	1.00E-04	1.00E-04
Taes_Yun_TW031	1.00E-04	1.00E-04	0.959449	0.033131	0.00722
Taes_Yun_TW032	0.989512	1.00E-04	1.00E-04	1.00E-04	0.010188
Taes_Yun_TW033	0.98595	0.000265	1.00E-04	1.00E-04	0.013586
Taes_Yun_TW034	0.981663	0.004232	1.00E-04	1.00E-04	0.013905
Tisp_Isp_B103	0.996122	0.003578	1.00E-04	1.00E-04	1.00E-04
Tisp_Isp_B104	0.996992	0.002708	1.00E-04	1.00E-04	1.00E-04
Tisp_Isp_B105	0.992049	0.004719	0.000828	1.00E-04	0.002305
Tisp_Isp_B106	0.984704	0.014996	1.00E-04	1.00E-04	1.00E-04
Tisp_Isp_B107	0.98675	0.001328	1.00E-04	1.00E-04	0.011722
Tisp_Isp_B108	0.974054	0.025646	1.00E-04	1.00E-04	1.00E-04
Tisp_Isp_B109	0.986175	1.00E-04	1.00E-04	1.00E-04	0.013525
Tkar_Geo_B110	0.982631	1.00E-04	1.00E-04	1.00E-04	0.017069

Accession	Q1	Q2	Q3	Q4	Q5
Tkar_Geo_B111	0.971499	0.02395	1.00E-04	1.00E-04	0.004352
Tkar_Geo_B112	0.999593	1.00E-04	1.00E-04	1.00E-04	0.000107
Tmon_Dom_A031	1.00E-04	0.998694	0.001006	1.00E-04	1.00E-04
Tmon_Dom_A032	0.003278	0.996422	1.00E-04	1.00E-04	1.00E-04
Tmon_Dom_A033	1.00E-04	0.9996	1.00E-04	1.00E-04	1.00E-04
Tmon_Dom_A034	1.00E-04	0.998707	0.000993	1.00E-04	1.00E-04
Tmon_Dom_A036	1.00E-04	0.987474	0.001187	1.00E-04	0.011139
Tmon_Dom_A037	0.005455	0.988708	1.00E-04	1.00E-04	0.005637
Tmon_Dom_A038	1.00E-04	0.9996	1.00E-04	1.00E-04	1.00E-04
Tmon_Dom_A040	1.00E-04	0.9996	1.00E-04	1.00E-04	1.00E-04
Tmon_Dom_A041	1.00E-04	0.9996	1.00E-04	1.00E-04	1.00E-04
Tmon_Dom_A042	0.020241	0.97946	1.00E-04	1.00E-04	1.00E-04
Tmon_Dom_A044	1.00E-04	0.9996	1.00E-04	1.00E-04	1.00E-04
Tmon_Dom_A045	1.00E-04	0.9996	1.00E-04	1.00E-04	1.00E-04
Tmon_Dom_A046	0.024894	0.974807	1.00E-04	1.00E-04	1.00E-04
Tmon_Dom_A047	1.00E-04	0.998792	0.000909	1.00E-04	1.00E-04
Tmon_Dom_A048	0.017755	0.981945	1.00E-04	1.00E-04	1.00E-04
Tmon_Dom_A050	0.012896	0.966965	1.00E-04	1.00E-04	0.019939
Tmon_Dom_A051	1.00E-04	0.978892	1.00E-04	1.00E-04	0.020809
Tmon_Dom_A053	1.00E-04	0.9996	1.00E-04	1.00E-04	1.00E-04
Tmon_Dom_A054	1.00E-04	0.985187	1.00E-04	1.00E-04	0.014513
Tmon_Dom_A055	0.029214	0.958322	1.00E-04	1.00E-04	0.012265
Tmon_Dom_A056	0.013959	0.985741	1.00E-04	1.00E-04	1.00E-04
Tmon_Dom_A057	0.004918	0.994782	1.00E-04	1.00E-04	1.00E-04
Tmon_Dom_A059	0.016893	0.962514	1.00E-04	1.00E-04	0.020393
Tmon_Dom_A060	1.00E-04	0.9996	1.00E-04	1.00E-04	1.00E-04
Tmon_Wil_A001	0.027526	0.962836	1.00E-04	1.00E-04	0.009438
Tmon_Wil_A002	0.013404	0.963633	1.00E-04	1.00E-04	0.022763
Tmon_Wil_A003	0.002902	0.996193	1.00E-04	1.00E-04	0.000705
Tmon_Wil_A004	0.025867	0.948669	1.00E-04	1.00E-04	0.025264
Tmon_Wil_A005	0.003113	0.985687	1.00E-04	1.00E-04	0.010999
Tmon_Wil_A006	0.034675	0.962589	0.002536	1.00E-04	1.00E-04
Tmon_Wil_A007	0.004629	0.9743	1.00E-04	1.00E-04	0.020871
Tmon_Wil_A008	0.017125	0.982575	1.00E-04	1.00E-04	1.00E-04
Tmon_Wil_A009	0.023952	0.952801	1.00E-04	1.00E-04	0.023047
Tmon_Wil_A010	0.033335	0.964323	1.00E-04	1.00E-04	0.002143
Tmon_Wil_A011	0.034445	0.937379	1.00E-04	1.00E-04	0.027976
Tmon_Wil_A012	0.022059	0.950941	1.00E-04	1.00E-04	0.026801
Tmon_Wil_A013	0.049591	0.89572	1.00E-04	1.00E-04	0.054489
Tmon_Wil_A014	0.012085	0.958168	1.00E-04	1.00E-04	0.029547
Tmon_Wil_A015	0.058863	0.940837	1.00E-04	1.00E-04	1.00E-04
Tmon_Wil_A017	0.03261	0.942697	1.00E-04	1.00E-04	0.024493
Tmon_Wil_A018	0.040294	0.935035	1.00E-04	1.00E-04	0.024471

Accession	Q1	Q2	Q3	Q4	Q5
Tmon_Wil_A019	0.079884	0.913315	1.00E-04	1.00E-04	0.006602
Tmon_Wil_A021	0.020665	0.957341	1.00E-04	1.00E-04	0.021794
Tmon_Wil_A022	0.035011	0.93602	1.00E-04	1.00E-04	0.028769
Tmon_Wil_A023	0.031119	0.954153	1.00E-04	1.00E-04	0.014528
Tmon_Wil_A024	0.029411	0.954473	1.00E-04	1.00E-04	0.015916
Tmon_Wil_A026	0.026393	0.969915	1.00E-04	1.00E-04	0.003492
Tmon_Wil_A027	0.010991	0.97976	1.00E-04	1.00E-04	0.00905
Tmon_Wil_A028	0.026905	0.961354	1.00E-04	1.00E-04	0.011541
Tmon_Wil_A029	0.072762	0.926938	1.00E-04	1.00E-04	1.00E-04
Tmon_Wil_A030	0.057358	0.93212	1.00E-04	1.00E-04	0.010322
Tpet_Xin_TW040	0.991187	1.00E-04	1.00E-04	1.00E-04	0.008513
Tpet_Xin_TW041	0.998882	1.00E-04	0.000819	1.00E-04	1.00E-04
Tpet_Xin_TW043	0.980232	0.000235	0.000811	1.00E-04	0.018622
Tpet_Xin_TW044	0.989129	0.010571	1.00E-04	1.00E-04	1.00E-04
Tsin_Dom_A061	1.00E-04	0.9996	1.00E-04	1.00E-04	1.00E-04
Ttur_Dom_B063	0.982452	0.00247	0.000279	1.00E-04	0.014699
Ttur_Dom_B064	0.98289	0.014869	1.00E-04	1.00E-04	0.002041
Ttur_Dom_B065	0.970914	0.022282	1.00E-04	1.00E-04	0.006604
Ttur_Dom_B066	0.99583	0.00387	1.00E-04	1.00E-04	1.00E-04
Ttur_Dom_B067	0.967253	1.00E-04	0.000317	1.00E-04	0.032229
Ttur_Dom_B068	0.99558	0.00412	1.00E-04	1.00E-04	1.00E-04
Ttur_Dom_B069	0.994621	0.005079	1.00E-04	1.00E-04	1.00E-04
Ttur_Dom_B070	0.980109	0.003136	1.00E-04	1.00E-04	0.016555
Ttur_Dom_B071	0.98733	0.01144	0.001029	1.00E-04	1.00E-04
Ttur_Dom_B072	0.964275	0.002638	1.00E-04	1.00E-04	0.032888
Ttur_Dom_B073	0.9996	1.00E-04	1.00E-04	1.00E-04	1.00E-04
Ttur_Dom_B074	0.983779	0.013953	1.00E-04	1.00E-04	0.002068
Ttur_Dom_B075	0.997119	0.001757	1.00E-04	1.00E-04	0.000924
Ttur_Dom_B076	0.97176	0.024969	1.00E-04	1.00E-04	0.003071
Ttur_Dom_B077	0.968959	0.024324	1.00E-04	1.00E-04	0.006517
Ttur_Dom_B078	0.9996	1.00E-04	1.00E-04	1.00E-04	1.00E-04
Ttur_Dom_B079	0.974956	0.008815	1.00E-04	1.00E-04	0.016029
Ttur_Dom_B080	0.961429	0.038271	1.00E-04	1.00E-04	1.00E-04
Ttur_Dom_B081	0.992041	1.00E-04	1.00E-04	1.00E-04	0.007659
Ttur_Dom_B082	0.995652	0.004048	1.00E-04	1.00E-04	1.00E-04
Ttur_Dom_B083	0.994447	0.005254	1.00E-04	1.00E-04	1.00E-04
Ttur_Dom_B084	0.990911	1.00E-04	1.00E-04	1.00E-04	0.008789
Ttur_Dom_B085	0.968938	0.021988	1.00E-04	1.00E-04	0.008874
Ttur_Dom_B086	0.996372	0.003328	1.00E-04	1.00E-04	1.00E-04
Ttur_Dom_B087	0.9749	0.01682	1.00E-04	1.00E-04	0.00808
Ttur_Dom_B088	0.97835	1.00E-04	1.00E-04	1.00E-04	0.02135
Ttur_Dom_B089	0.990843	1.00E-04	1.00E-04	1.00E-04	0.008857
Ttur_Dom_B090	0.982888	0.016812	1.00E-04	1.00E-04	1.00E-04

Accession	Q1	Q2	Q3	Q4	Q5
Ttur_Dom_B091	0.960145	0.039556	1.00E-04	1.00E-04	1.00E-04
Ttur_Dur_B113	0.984633	0.012581	1.00E-04	1.00E-04	0.002586
Ttur_Dur_B114	0.989893	1.00E-04	1.00E-04	1.00E-04	0.009807
Ttur_Dur_B115	0.997222	1.00E-04	1.00E-04	1.00E-04	0.002479
Ttur_Dur_B116	0.997236	0.002464	1.00E-04	1.00E-04	1.00E-04
Ttur_Dur_B117	0.9996	1.00E-04	1.00E-04	1.00E-04	1.00E-04
Ttur_Dur_B118	0.988921	1.00E-04	1.00E-04	1.00E-04	0.010779
Ttur_Dur_B119	0.9996	1.00E-04	1.00E-04	1.00E-04	1.00E-04
Ttur_Dur_B120	0.9996	1.00E-04	1.00E-04	1.00E-04	1.00E-04
Ttur_Dur_B121	0.996579	0.003121	1.00E-04	1.00E-04	1.00E-04
Ttur_Dur_B122	0.979999	0.018699	1.00E-04	1.00E-04	0.001102
Ttur_Dur_B123	0.970303	0.025746	1.00E-04	1.00E-04	0.003751
Ttur_Dur_B124	0.99619	0.00351	1.00E-04	1.00E-04	1.00E-04
Ttur_Dur_B125	0.98318	1.00E-04	1.00E-04	1.00E-04	0.01652
Ttur_Kho_B053	0.9996	1.00E-04	1.00E-04	1.00E-04	1.00E-04
Ttur_Kho_B054	0.976323	1.00E-04	1.00E-04	1.00E-04	0.023377
Ttur_Kho_B055	0.97119	1.00E-04	1.00E-04	1.00E-04	0.02851
Ttur_Kho_B056	0.978799	0.020901	1.00E-04	1.00E-04	1.00E-04
Ttur_Kho_B057	0.983374	0.016326	1.00E-04	1.00E-04	1.00E-04
Ttur_Kho_B058	0.95575	0.028884	1.00E-04	1.00E-04	0.015166
Ttur_Kho_B059	0.980984	1.00E-04	1.00E-04	1.00E-04	0.018716
Ttur_Kho_B060	0.989518	1.00E-04	1.00E-04	1.00E-04	0.010183
Ttur_Kho_B061	0.967559	0.01538	1.00E-04	1.00E-04	0.01686
Ttur_Kho_B062	0.9996	1.00E-04	1.00E-04	1.00E-04	1.00E-04
Ttur_Per_B093	0.970124	0.021098	1.00E-04	1.00E-04	0.008577
Ttur_Per_B094	0.971676	0.028024	1.00E-04	1.00E-04	1.00E-04
Ttur_Per_B096	0.989858	1.00E-04	1.00E-04	1.00E-04	0.009842
Ttur_Per_B097	0.969416	0.025612	1.00E-04	1.00E-04	0.004773
Ttur_Per_B098	0.998767	1.00E-04	0.000934	1.00E-04	1.00E-04
Ttur_Per_B099	0.949799	0.049901	1.00E-04	1.00E-04	1.00E-04
Ttur_Per_B100	0.995932	1.00E-04	1.00E-04	1.00E-04	0.003768
Ttur_Per_B101	0.9996	1.00E-04	1.00E-04	1.00E-04	1.00E-04
Ttur_Per_B102	0.992665	0.007035	1.00E-04	1.00E-04	1.00E-04
Ttur_Pol_B013	0.988527	0.011173	1.00E-04	1.00E-04	1.00E-04
Ttur_Pol_B014	0.990705	1.00E-04	1.00E-04	1.00E-04	0.008995
Ttur_Pol_B015	0.974559	0.020833	1.00E-04	1.00E-04	0.004409
Ttur_Pol_B016	0.982077	0.003625	1.00E-04	1.00E-04	0.014099
Ttur_Pol_B017	0.983069	0.013218	1.00E-04	1.00E-04	0.003513
Ttur_Pol_B018	0.9996	1.00E-04	1.00E-04	1.00E-04	1.00E-04
Ttur_Pol_B019	0.989798	1.00E-04	1.00E-04	1.00E-04	0.009902
Ttur_Pol_B020	0.992426	0.007274	1.00E-04	1.00E-04	1.00E-04
Ttur_Pol_B021	0.983219	0.016481	1.00E-04	1.00E-04	1.00E-04
Ttur_Pol_B022	0.935314	0.05027	1.00E-04	1.00E-04	0.014216

Accession	Q1	Q2	Q3	Q4	Q5
Ttur_Riv_B001	0.984293	1.00E-04	1.00E-04	1.00E-04	0.015407
Ttur_Riv_B002	0.98481	0.014891	1.00E-04	1.00E-04	1.00E-04
Ttur_Riv_B003	0.977016	1.00E-04	1.00E-04	1.00E-04	0.022684
Ttur_Riv_B004	0.986678	0.012841	1.00E-04	1.00E-04	0.000281
Ttur_Riv_B005	0.980302	0.011691	1.00E-04	1.00E-04	0.007807
Ttur_Riv_B006	0.982318	1.00E-04	1.00E-04	1.00E-04	0.017382
Ttur_Riv_B007	0.967358	1.00E-04	1.00E-04	1.00E-04	0.032342
Ttur_Riv_B008	0.9996	1.00E-04	1.00E-04	1.00E-04	1.00E-04
Ttur_Riv_B009	0.990794	0.008906	1.00E-04	1.00E-04	1.00E-04
Ttur_Riv_B010	0.995251	0.003283	1.00E-04	1.00E-04	0.001267
Ttur_Riv_B011	0.974079	1.00E-04	1.00E-04	1.00E-04	0.025621
Ttur_Riv_B012	0.9996	1.00E-04	1.00E-04	1.00E-04	1.00E-04
Ttur_Ukn_B051	0.994232	1.00E-04	1.00E-04	1.00E-04	0.005468
Ttur_Ukn_B092	0.078034	0.424871	0.008544	1.00E-04	0.488451
Ttur_Unk_B043	0.103943	0.439693	0.00839	1.00E-04	0.447873
Ttur_Wil_B023	0.971045	0.026263	1.00E-04	1.00E-04	0.002493
Ttur_Wil_B024	0.993799	0.005901	1.00E-04	1.00E-04	1.00E-04
Ttur_Wil_B025	0.978355	0.021345	1.00E-04	1.00E-04	1.00E-04
Ttur_Wil_B026	0.974297	1.00E-04	1.00E-04	1.00E-04	0.025403
Ttur_Wil_B027	0.947971	0.051729	1.00E-04	1.00E-04	1.00E-04
Ttur_Wil_B028	0.988897	0.000944	1.00E-04	1.00E-04	0.009959
Ttur_Wil_B029	0.970351	0.009309	0.000822	1.00E-04	0.019418
Ttur_Wil_B030	0.980072	0.006319	1.00E-04	1.00E-04	0.013409
Ttur_Wil_B031	0.9996	1.00E-04	1.00E-04	1.00E-04	1.00E-04
Ttur_Wil_B032	0.995306	0.004394	1.00E-04	1.00E-04	1.00E-04
Ttur_Wil_B033	0.958861	0.03288	1.00E-04	1.00E-04	0.008059
Ttur_Wil_B034	0.990624	1.00E-04	1.00E-04	1.00E-04	0.009076
Ttur_Wil_B035	0.970769	0.028001	1.00E-04	1.00E-04	0.00103
Ttur_Wil_B036	0.997684	0.001997	1.00E-04	1.00E-04	0.000118
Ttur_Wil_B037	0.977014	0.022687	1.00E-04	1.00E-04	1.00E-04
Ttur_Wil_B038	0.973744	1.00E-04	1.00E-04	1.00E-04	0.025956
Ttur_Wil_B039	0.997694	0.002006	1.00E-04	1.00E-04	1.00E-04
Ttur_Wil_B040	0.944151	0.033647	1.00E-04	1.00E-04	0.022003
Ttur_Wil_B041	0.9996	1.00E-04	1.00E-04	1.00E-04	1.00E-04
Ttur_Wil_B042	0.954877	0.021511	1.00E-04	1.00E-04	0.023413
Ttur_Wil_B044	0.976268	0.023433	1.00E-04	1.00E-04	1.00E-04
Ttur_Wil_B045	0.995316	0.004384	1.00E-04	1.00E-04	1.00E-04
Ttur_Wil_B046	0.987125	0.012575	1.00E-04	1.00E-04	1.00E-04
Ttur_Wil_B047	0.985658	0.014042	1.00E-04	1.00E-04	1.00E-04
Ttur_Wil_B048	0.982613	0.015308	1.00E-04	1.00E-04	0.001879
Ttur_Wil_B049	0.9996	1.00E-04	1.00E-04	1.00E-04	1.00E-04
Ttur_Wil_B050	0.997515	0.001974	1.00E-04	1.00E-04	0.000311
Ttur_Wil_B052	0.991286	1.00E-04	1.00E-04	1.00E-04	0.008414

Accession	Q1	Q2	Q3	Q4	Q5
Tunk_Unk_TW042	0.974752	0.024948	1.00E-04	1.00E-04	1.00E-04
Tura_Ura_A025	0.01797	1.00E-04	1.00E-04	1.00E-04	0.98173
Tura_Ura_A062	0.00718	0.017577	1.00E-04	1.00E-04	0.975043
Tura_Ura_A064	1.00E-04	1.00E-04	1.00E-04	1.00E-04	0.9996
Tura_Ura_A065	1.00E-04	1.00E-04	1.00E-04	1.00E-04	0.9996
Tura_Ura_A066	0.029411	1.00E-04	1.00E-04	1.00E-04	0.970289
Tura_Ura_A067	1.00E-04	1.00E-04	1.00E-04	1.00E-04	0.9996
Tura_Ura_A068	0.00861	1.00E-04	1.00E-04	1.00E-04	0.991091
Tura_Ura_A069	0.003623	0.002717	1.00E-04	1.00E-04	0.99346
Tura_Ura_A070	1.00E-04	1.00E-04	1.00E-04	1.00E-04	0.9996
Tura_Ura_A071	0.004882	1.00E-04	1.00E-04	1.00E-04	0.994818
Tura_Ura_A072	1.00E-04	1.00E-04	1.00E-04	1.00E-04	0.9996
Tura_Ura_A073	1.00E-04	0.020332	1.00E-04	1.00E-04	0.979369
Tura_Ura_A074	1.00E-04	0.013597	1.00E-04	1.00E-04	0.986103
Tura_Ura_A076	0.024891	1.00E-04	1.00E-04	1.00E-04	0.974809
Tura_Ura_A077	0.003313	0.039628	1.00E-04	1.00E-04	0.956859
Tura_Ura_A078	1.00E-04	1.00E-04	1.00E-04	1.00E-04	0.9996
Tura_Ura_A079	0.027816	0.009345	1.00E-04	1.00E-04	0.962639
Tura_Ura_A080	1.00E-04	1.00E-04	1.00E-04	1.00E-04	0.9996
Tura_Ura_A081	1.00E-04	1.00E-04	1.00E-04	1.00E-04	0.9996
Tura_Ura_A082	0.013127	0.017059	1.00E-04	1.00E-04	0.969614
Tura_Ura_A083	0.007784	0.020157	1.00E-04	1.00E-04	0.97186
Tura_Ura_A085	1.00E-04	0.009944	1.00E-04	1.00E-04	0.989757
Tura_Ura_A086	0.011417	1.00E-04	1.00E-04	1.00E-04	0.988283
Tura_Ura_A087	0.007305	1.00E-04	1.00E-04	1.00E-04	0.992395
Tura_Ura_A088	1.00E-04	0.026232	1.00E-04	1.00E-04	0.973468
Tura_Ura_A091	1.00E-04	1.00E-04	1.00E-04	1.00E-04	0.9996
Tura_Wil_A090	1.00E-04	0.991556	1.00E-04	1.00E-04	0.008144
Tvav_Vav_TW045	0.947152	0.044647	1.00E-04	1.00E-04	0.008001
Tvav_Vav_TW046	0.994552	0.005148	1.00E-04	1.00E-04	1.00E-04
Tvav_Vav_TW047	0.996771	0.002929	1.00E-04	1.00E-04	1.00E-04
Tvav_Vav_TW048	0.990972	1.00E-04	1.00E-04	1.00E-04	0.008728
Tvav_Vav_TW049	0.98434	0.01074	1.00E-04	1.00E-04	0.004721

Each color represents group of accessions sharing over 50% of their ancestral proportion. Unshaded accessions are those with higher level of admixture harboring less than 50% ancestral proportion of any of the groups.

International Journal of
Molecular Sciences

MDPI

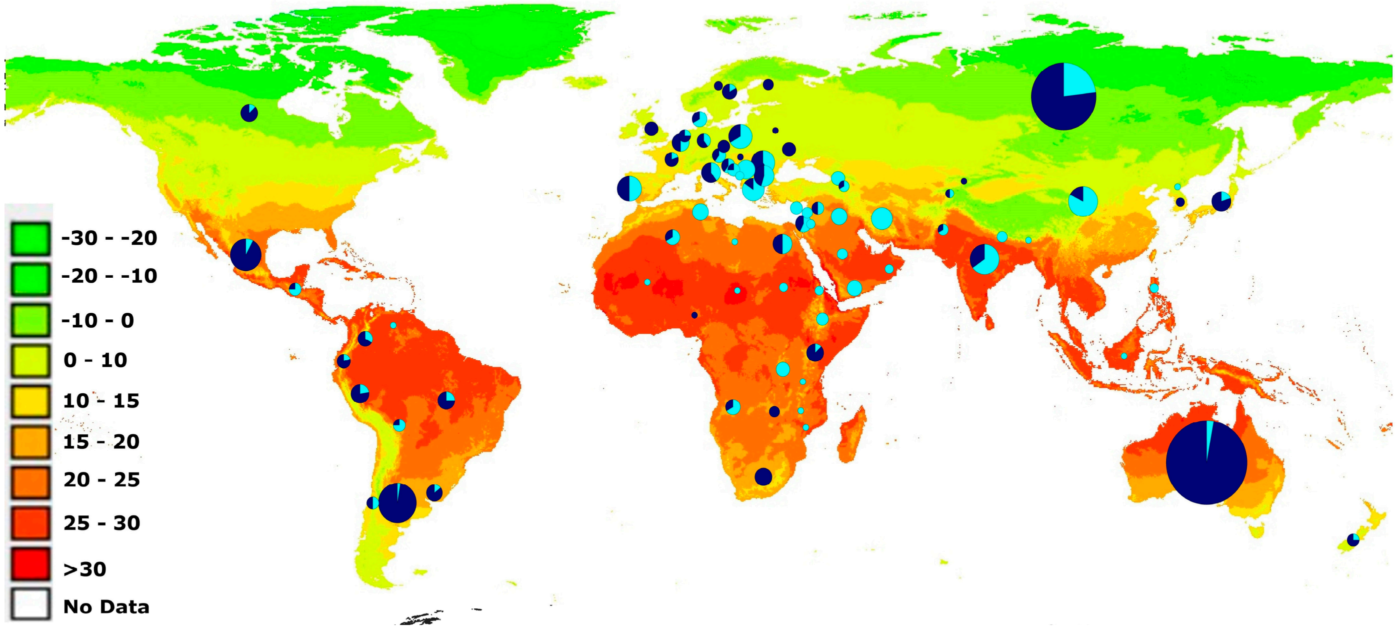

Figure S3. Geographic origin of the accessions in exome (113K) dataset mapped against annual temperature (°C) distribution. Pie diagrams show the cultivars in dark blue and the landraces in light blue. The size of the circles is proportional with the number of accessions.
